# Expression characteristics and their functional role of IGFBP gene family in pan-cancer

**DOI:** 10.1186/s12885-023-10832-3

**Published:** 2023-04-24

**Authors:** Yingnan Liu, Shixuan Shen, Ziwei Yan, Lirong Yan, Hanxi Ding, Ang Wang, Qian Xu, Liping Sun, Yuan Yuan

**Affiliations:** 1 Tumor Etiology and Screening Department of Cancer Institute and General Surgery, The First Hospital of China Medical University, No. 155 North Nanjing Street, Heping District, Shenyang, 110001 Liaoning People’s Republic of China; 2 Key Laboratory of Cancer Etiology and Prevention in Liaoning Education Department, The First Hospital of China Medical University, Shenyang, 110001 China; 3 Key Laboratory of GI Cancer Etiology and Prevention in Liaoning Province, The First Hospital of China Medical University, Shenyang, 110001 China

**Keywords:** IGFBP, Pan-cancer, Expression, Prognosis, Cancer-related pathway, Tumor immune microenvironment, Immune infiltration, Mutation

## Abstract

**Background:**

Insulin-like growth factor binding proteins (IGFBPs) are critical regulators of the biological activities of insulin-like growth factors. The IGFBP family plays diverse roles in different types of cancer, which we still lack comprehensive and pleiotropic understandings so far.

**Methods:**

Multi-source and multi-dimensional data, extracted from The Cancer Genome Atlas (TCGA), Oncomine, Cancer Cell Line Encyclopedia (CCLE), and the Human Protein Atlas (HPA) was used for bioinformatics analysis by R language. Immunohistochemistry and qRT-PCR were performed to validate the results of the database analysis results. Bibliometrics and literature review were used for summarizing the research progress of IGFBPs in the field of tumor.

**Results:**

The members of IGFBP gene family are differentially expressed in various cancer types. IGFBPs expression can affect prognosis of different cancers. The expression of IGFBPs expression is associated with multiple signal transduction pathways. The expression of IGFBPs is significantly correlated with tumor mutational burden, microsatellite instability, tumor stemness and tumor immune microenvironment. The qRT-PCR experiments verified the lower expression of IGFBP2 and IGFBP6 in gastric cancer and the lower expression of IGFBP6 in colorectal cancer. Immunohistochemistry validated a marked downregulation of IGFBP2 protein in gastric cancer tissues. The keywords co-occurrence analysis of IGFBP related publications in cancer showed relative research have been more concentrating on the potential of IGFBPs as tumor diagnostic and prognostic markers and developing cancer therapies.

**Conclusions:**

These findings provide frontier trend of IGFBPs related research and new clues for identifying novel therapeutic targets for various cancers.

**Supplementary Information:**

The online version contains supplementary material available at 10.1186/s12885-023-10832-3.

## Background

As we know, tumors are often exhibited their characteristics by both homogeneity and heterogeneity. For example, tumors from different organs could share the same molecular signatures [1], while tumors from the same organs may have different molecular characteristics [2]. Therefore, a comprehensive pan-cancer analysis for certain molecules across multiple tumor types is of great significance. Analysis for pan-cancer can identify the differences and similarities of the key biological processes among various cancer types from different anatomical site [3] and provide clues for developing new combination and targeted therapies for cancer.

Insulin-like growth factor binding proteins (IGFBPs) are flexible endocrine and autocrine/paracrine regulators of the bioactivity of Insulin-like growth factor (IGF) [4]. The members of the IGFBP family include IGFBP1, IGFBP 2, IGFBP3, IGFBP4, IGFBP5, IGFBP6, IGFBP7 and IGFBPL1. They have crucial functions such as transcriptional regulation and induction of apoptosis [5], and play important roles in human cancers. For example, IGFBP1 and IGFBP2 are closely related to human insulin sensitivity [6]; IGFBP2 may inhibit the effective ways of tumor growth and metastasis [7]. IGFBP3 blocks the hyaluronic acid CD44 signaling pathway through a p53-dependent mechanism [8]. IGFBP4 can increase the bioavailability of IGF and stimulate IGF-mediated growth [9]. IGFBP5 has the function of reducing neovascularization [10]. IGFBP6 is a relatively specific inhibitor of IGF-II, playing crucial roles in inhibiting cancer cell migration [11]. IGFBP7 has the function of stimulating the production of prostacyclin (PGI2) and stimulating cell adhesion [12, 13]. The inactivation of IGFBPL1 is associated with the pathogenesis of breast cancer [14]; moreover, IGFBPL1 inhibits the growth of cancer cell by inhibiting the signaling pathway PI3K-AKT in esophageal cancer [15]. However, previous research on the relationship between IGFBPs and human tumors usually focused on a single gene in the most common cancers, such as breast cancer, lung cancer, and gastric cancer, etc. The family member IGFBP4, IGFBP5, IGFBPL1 has been less studied in relation to cancer compared with other members.

This study utilized a set of multi-source and multi-dimensional data to characterize the expression profiles of IGFBP gene family, the interaction between IGFBPs, and its relationship with cancer-related pathways, prognosis, Tumor Mutational Burden (TMB), Microsatellite Instability (MSI), tumor stemness, tumor immune microenvironment, immune cell infiltration, comorbidity factors, and gene alteration characteristics of pan-cancer. The results of the database analysis results using immunohistochemistry and qRT-PCR. We intend to discover the potential of IGFBPs as new biomarkers for cancer diagnosis or prognosis as well as therapeutic target, and shed new light on relevant mechanism. Further, bibliometrics and literature review were used for summarizing the research progress of IGFBP family.

## Materials and methods

### Data collection from multi-source databases

We extracted the expression and gene alteration data of IGFBPs in 33 types of cancer (Additional file 1: Table S1) from TCGA (http://cancergenome.nih.gov/). Besides, the survival data and tumor stemness characteristics (based on DNA methylation and RNA expression) were downloaded from UCSC Xena (https://xenabrowser.net/). The Oncomine database (https://www.oncomine.org/) was used to verify the expression level of IGFBPs between different cancer and normal tissues. The expression and gene alteration data for IGFBPs in different cancer cell lines was downloaded from CCLE database (https://portals.broadinstitute.org/ccle), 325 cell lines in 8 tumors were included in the analysis. The HPA database (https://www.proteinatlas.org/) was used to collect the immunohistochemical staining data of IGFBP family in 20 types of tumors.

### Expression profiling analysis of IGFBPs at multidimensional levels

#### Expression profiles of IGFBPs at mRNA level

The gene expression data from TCGA was analyzed using the R package “Deseq2” [16] to determine the differentially expressed IGFBPs, *P* < 0.05 indicated a statistically significant difference. The Oncomine database was used to validate the differential genes applying the following thresholds: |fold change|> 2, *P* < 0.05 and gene rank = top 10%.

#### Expression profiles of IGFBPs at cell level

The expression profile of IGFBPs in various cancer cell lines were calculated using the Kruskal–Wallis rank test, differences were considered significant when *P* < 0.05.

#### Expression profiles of IGFBPs at protein level

We obtained data on immunohistochemistry of IGFBP family from the HPA database, which has information on 20 different cancer types. According to the intensity and proportion of staining, the protein expression levels of IGFBPs were grouped into high, medium, low and not detected.

#### Comorbidity factors associated with IGFBPs expression in pan-cancer

Using the Kruskal–Wallis rank sum test, we further investigated the comorbidity factors (age and gender) associated with IGFBPs expression in pan-cancer.

### Correlation between IGFBPs expression and prognosis in pan-cancer

According to the median expression levels of IGFBPs, all the cancer samples were classified into two groups, and the overall survival was compared using the log-rank test.

### Correlation between IGFBPs expression and hallmark pathways and interaction between IGFBPs

Gene set variation analysis (GSVA) provides an algorithm to estimate the variation of pathway activity [17]. In the present study, the Pearson correlation coefficients were calculated to find out the hallmark pathways related to the expression of IGFBPs, significantly correlating pathways were selected using | r |> 0.3 and *P* < 0.05.

To investigate the correlation and interaction of IGFBPs expression, we calculated the Pearson’s correlation coefficient of IGFBPs expression using TCGA data. R package “ggplot2” [18] was used for visualization. Furthermore, the GENEMANIA (http://genemania.org/) and STRING (https://string-db.org/) databases were applied to predict the network to explore the potential interaction relationships of IGFBPs. Cytoscape 3.9.1 was used for visualization.

### Correlation between IGFBPs expression and multiple molecular features

#### Correlation between IGFBPs expression and TMB as well as MSI

TMB and MSI are regarded as indicators for immune checkpoint inhibitor activity [19, 20]. Thus, we calculated the Spearman coefficients between IGFBPs expression levels and TMB as well as MSI scores obtained from TCGA. Correlations were considered as significant when | r |> 0.2 and *P* < 0.05.

#### Correlation between IGFBPs expression and tumor stemness score

Tumor stemness score RNAss and DNAss obtained through RNA sequencing data and DNA methylation data respectively were used to measure the degree of oncogenic dedifferentiation [21]. Spearman coefficients between IGFBPs expression and stemness scores were calculated. *P* < 0.05 was statistically significant.

#### Correlation between IGFBPs expression and tumor immune microenvironment

The ESTIMATE scores for each patient from TCGA were calculated by the “estimate” R package [22] to reflect the amount of immune and stromal components at tumor immune microenvironment in pan-cancer tissues, while the estimate score was used to forecast tumor purity. We performed Spearman’s correlation analysis between IGFBPs expression and the ESTIMATE immune/ stromal/ estimate scores. A difference of *P* < 0.05 was considered significant.

We utilized data from the CIBERSORT [23] platform to perform Spearman correlation analysis between the expression of IGFBPs and infiltration levels for 22 types of immune cells in pan-cancer. Correlations were considered as significant when | r |> 0.3 and *P* < 0.05.

### Correlation between IGFBPs expression and its genetic variation

The gene mutation data of IGFBPs in various cancer tissues and cells was downloaded from TCGA and CCLE databases respectively, while information for IGFBPs copy number variation (CNV) frequencies was extracted from TCGA database. The mutation and CNV proportion were indicated by the mutation frequencies and deletion or amplification CNV rates. Besides, to determine the impact of mutations and CNVs on IGFBPs expression, the Mann–Whitney U test was performed.

### Verification of IGFBPs expression at mRNA and protein levels

#### Sample source

This study was approved by the Ethics Approval Committee of the First Hospital of China Medical University, and informed consent was signed by the participants. In total, 67 pairs of gastric cancer and adjacent tissues, as well as 44 pairs of colorectal cancer and adjacent tissues were used to extract total RNA for qRT-PCR experiments. We further retrospectively collected gastric cancer tissue specimens for immunohistochemical staining. A total of 18 gastric cancer and 20 adjacent normal specimens were detected. Tissue specimens were embedded in paraffin following fixation in 10% formalin.

#### Validation of the mRNA expression levels of IGFBPs by qRT-PCR

RNA was extracted and reversed to cDNA; real-time quantitative PCR (qRT-PCR) was performed to detect the relative mRNA levels of IGFBPs in a 20μL reaction system. The fold change was determined via the method of 2 − ΔΔCt [ΔΔCt = (ΔCt of IGFBPs) − (ΔCt of β-actin)]. The test results were analyzed using the Wilkson test of the non-parametric test in SPSS software, and *P* < 0.05 was considered statistically significant. The primer sequences of IGFBPs and β-actin were showed in Additional file 1: Table S2.

#### Validation of the protein expression levels of IGFBPs by immunohistochemistry

Immunohistochemistry (IHC) staining was used to detect the protein expression of IGFBP2 in gastric cancer tissues. The paraffin-embedded tissue specimens were cut into 4 μm-thick sections, which were then deparaffinized, rehydrated with gradient ethanol, and incubated in EDTA. Endogenous peroxidase was blocked using 3% hydrogen peroxide. 10% normal goat serum was used to destroy tissue collagen for reducing non-specific binding. The rabbit monoclonal antibody to IGFBP2 (ab188200, 1:1000, Abcam, UK) was used as the primary antibody, and the samples were incubated for 1 h at room temperature. After washing with PBS, biotin-labeled secondary antibodies and streptavidin–horseradish peroxidase were added to the samples, respectively, and incubated at room temperature for 10 min each time. The samples were then dyed with DAB, dehydrated and fixed with resin.

Finally, the stained tissue sections were observed by experienced pathologists under the microscope. uses the widely accepted HSCORE (histological score) system to semi-quantitatively evaluate the protein expression level in the tissues [24, 25]. The formula of HSCORE is: HSCORE = ∑Pi (i + 1), where i is the staining intensity of the cell (0: negative, 1: weak, 2: moderate, and 3: strong), and Pi is the percentage of cells at each intensity level (0–100%). The results were analyzed by SPSS software, Mann–Whitney U and Wilcoxon matched pairs signed-rank test were performed for non-parametric tests. *P* < 0.05 was considered statistically significant.

### Bibliometrics and literature review

Publications on research related to IGFBPs in cancer were searched in the Web of Science Core Collection for bibliometric analysis and literature review with the following search strategy: Topic search (TS) = (Insulin-like growth factor binding protein) and TS = (cancer). The dates of publication were restricted from Jan 1st, 2000 to Dec 31st, 2021, type of publication was limited to articles, and the language was restricted to English. Keywords co-occurrence analysis was performed using VOSviewer (version 1.6.18) [26].

## Results

### The expression profile of IGFBPs in pan-cancer

#### IGFBPs expression profile at mRNA level

**Fig. 1:**
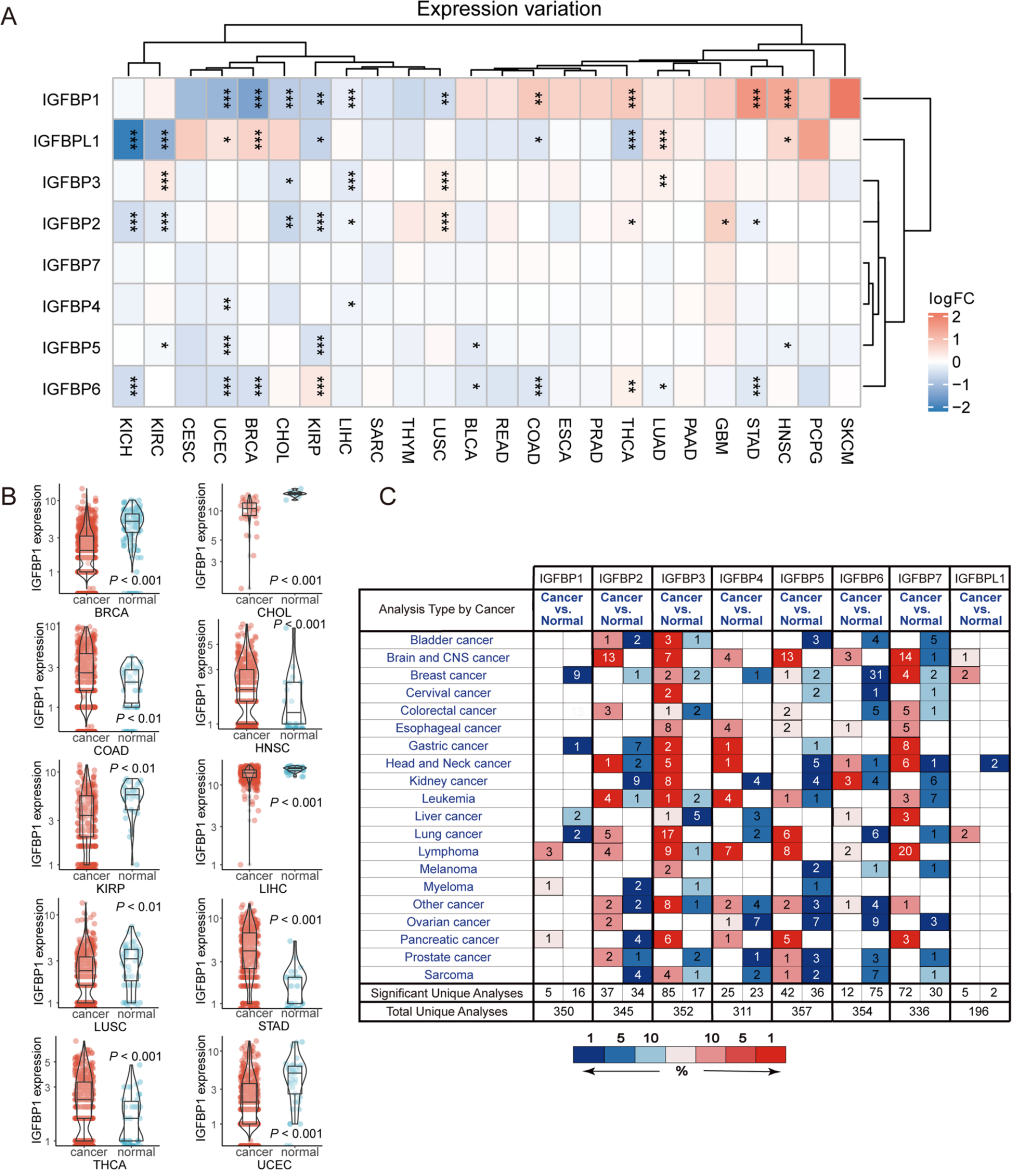
Expression profile of IGFBPs across different cancer types. **A** Expression of IGFBPs in different cancer and normal samples. The color in heatmap represents the log2 fold change value between cancer and normal. The blue color represents the low expression in cancer, whereas the red color represents the high expression in cancer. The * sign represents degree of statistical significance, * *P* < 0.05, ** *P* < 0.01, *** *P* < 0.001. **B** Expression of IGFBP1 in 10 types of cancers between cancer and normal tissues. **C** Expression of IGFBPs in various cancers from Oncomine database. The blue color represents the low expression, whereas the red color represents the high expression

We used the count data of 33 types of tumors covered by TCGA to analyze the expression levels of the IGFBP family in general. The results showed that the expression of IGFBPs were different in multiple cancers (Table 1, Fig. 1A). We selected IGFBP1, which had differential expression in most cancers and larger fold changes to further visualize its expression in 10 types of cancers and normal tissues, and presented in the form of mean ± standard deviation (Fig. 1B).

**Table 1:** Differential expression of IGFBPs at mRNA level in pan-cancer

Gene	CancerType	Differential expression	Gene	CancerType	Differential expression
IGFBP1	BRCA	down-regulated	IGFBP4	UCEC	down-regulated
IGFBP1	CHOL	down-regulated	IGFBP5	BLCA	down-regulated
IGFBP1	KIRP	down-regulated	IGFBP5	HNSC	down-regulated
IGFBP1	LIHC	down-regulated	IGFBP5	KIRC	down-regulated
IGFBP1	LUSC	down-regulated	IGFBP5	KIRP	down-regulated
IGFBP1	UCEC	down-regulated	IGFBP5	UCEC	down-regulated
IGFBP1	COAD	up-regulated	IGFBP6	BLCA	down-regulated
IGFBP1	HNSC	up-regulated	IGFBP6	BRCA	down-regulated
IGFBP1	STAD	up-regulated	IGFBP6	COAD	down-regulated
IGFBP1	THCA	up-regulated	IGFBP6	KICH	down-regulated
IGFBP2	CHOL	down-regulated	IGFBP6	LUAD	down-regulated
IGFBP2	KICH	down-regulated	IGFBP6	STAD	down-regulated
IGFBP2	KIRC	down-regulated	IGFBP6	UCEC	down-regulated
IGFBP2	KIRP	down-regulated	IGFBP6	KIRP	up-regulated
IGFBP2	LIHC	down-regulated	IGFBP6	THCA	up-regulated
IGFBP2	STAD	down-regulated	IGFBPL1	COAD	down-regulated
IGFBP2	GBM	up-regulated	IGFBPL1	KICH	down-regulated
IGFBP2	LUSC	up-regulated	IGFBPL1	KIRC	down-regulated
IGFBP2	THCA	up-regulated	IGFBPL1	KIRP	down-regulated
IGFBP3	CHOL	down-regulated	IGFBPL1	THCA	down-regulated
IGFBP3	LIHC	down-regulated	IGFBPL1	BRCA	up-regulated
IGFBP3	KIRC	up-regulated	IGFBPL1	HNSC	up-regulated
IGFBP3	LUAD	up-regulated	IGFBPL1	LUAD	up-regulated
IGFBP3	LUSC	up-regulated	IGFBPL1	UCEC	up-regulated
IGFBP4	LIHC	down-regulated

The Oncomine database was used to verify the findings from TCGA (Fig. 1C). The results indicated that, consistent with our TCGA findings, the IGFBP genes were differentially expressed in various cancer types.

#### IGFBPs expression profile at cell level

**Fig. 2:**
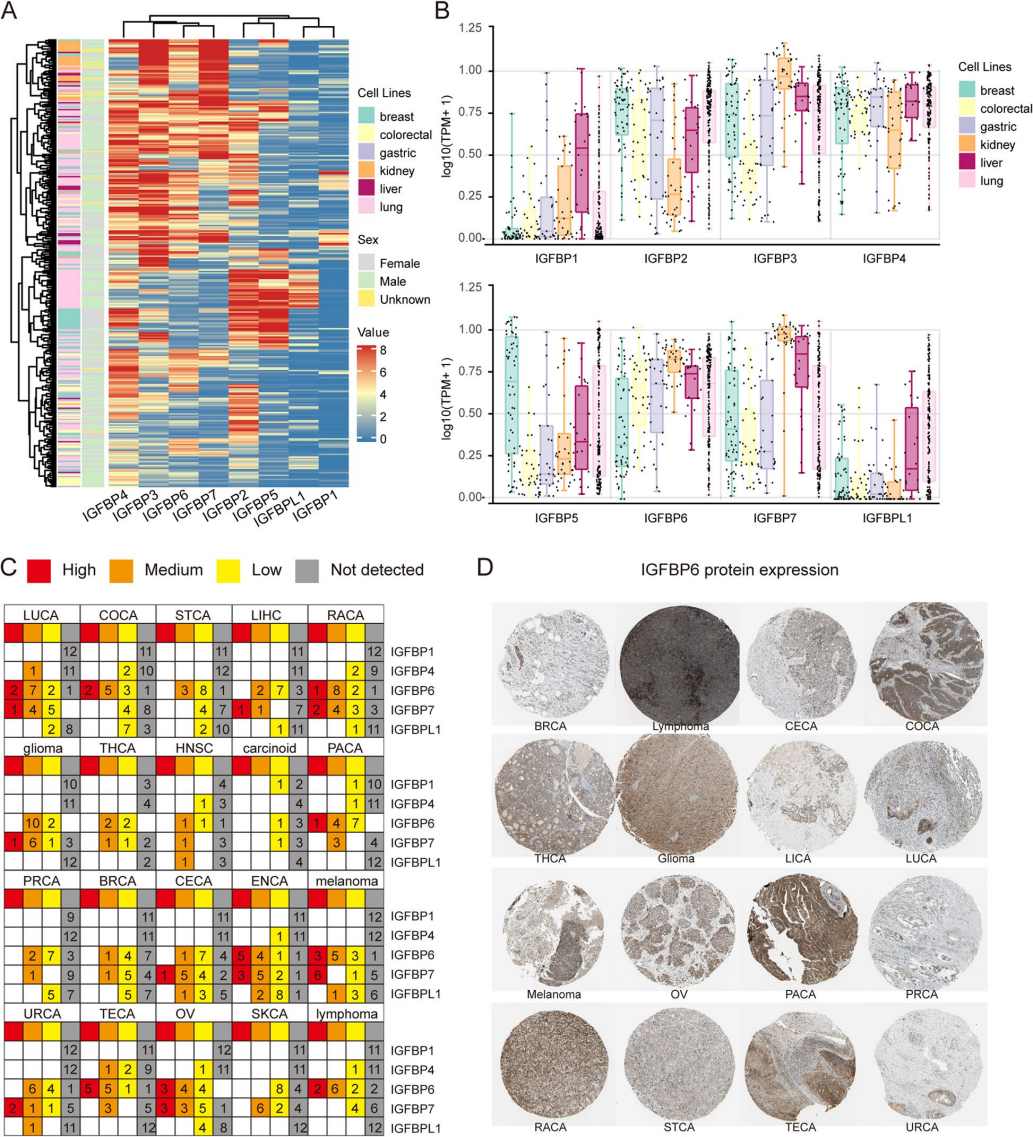
The expression profile of IGFBPs at cell and protein level. **A** Expression heatmap of IGFBPs in 6 cancer cell lines (breast, colorectal, gastric, kidney, liver and lung) from CCLE database. The blue color represents the low expression, whereas the red color represents the high expression. **B** Boxplot of IGFBPs expression in 6 cancer cell lines. **C** IGFBPs protein expression across various cancer types. The expression of each gene in each cancer was divided into four groups of high expression (red color), medium expression (orange color), low expression (yellow color) and not detected (grey color). **D** IGFBP6 protein expression in 16 cancer types based on immunohischemistry staining results from 'The Human Protein Atlas' database

The results of CCLE analysis showed different IGFBPs exhibited distinct expression levels in different cancer cell lines (Fig. 2A). Among them, IGFBP2, IGFBP3, IGFBP4, IGFBP6 and IGFBP7 showed higher expression levels, especially in gastric, liver and lung cancer cell lines; conversely, IGFBP1, IGFBP5 and IGFBPL1 showed relatively lower expression, particularly in colorectal, gastric and kidney cancer cell lines (Fig. 2B).

#### IGFBPs expression profile at the protein level

We collected the protein expression information of IGFBP1, IGFBP4, IGFBP6, IGFBP7, and IGFBPL1 from the HPA database. The results indicated that the protein expression of IGFBP6 and IGFBP7 could be detectable in all 20 types of tumor tissues (Additional file 1: Table S3). By contrast, IGFBP1 and IGFBP4 protein showed medium or low expression in several tumors such as pancreatic cancer and colorectal cancer (Fig. 2C). In ENCA and OV, the protein expression of IGFBPs was the most. In addition, Fig. 2D displayed representative immunohistochemical pictures depicting the IGFBP6 protein expression in different cancer tissues.

#### Comorbidity factors associated with IGFBPs expression in pan-cancer

**Fig. 3:**
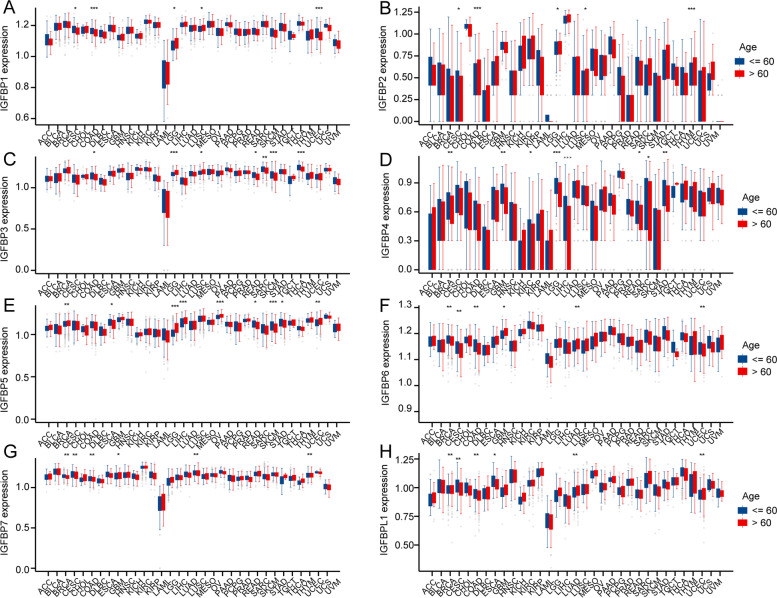
Correlation between IGFBPs expression and age of patients in pan-cancer. Blue bars represent younger age (< = 60), while red bars represent older age (> 60). * *P* < 0.05, ** *P* < 0.01, *** *P* < 0.001

**Fig. 4:**
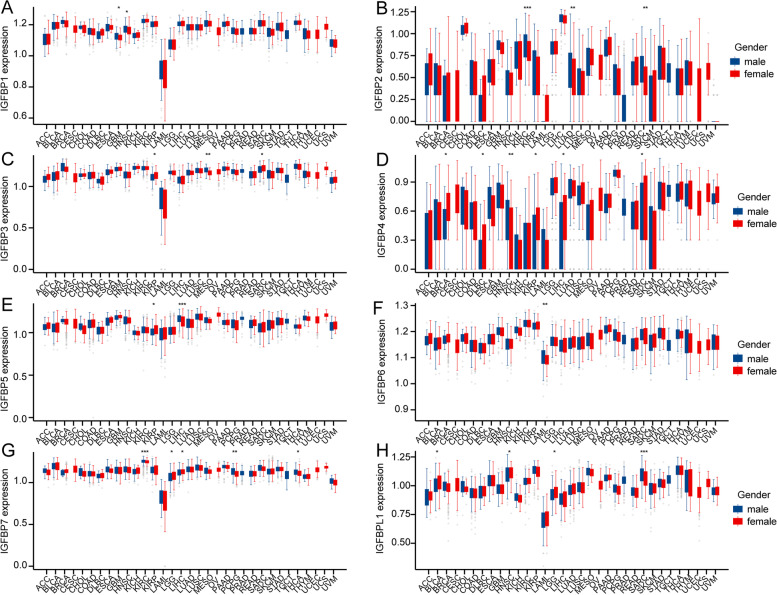
Correlation between IGFBPs expression and gender of patients in pan-cancer. Blue bars represent male, while red bars represent female. * *P* < 0.05, ** *P* < 0.01, *** *P* < 0.001

The correlation between IGFBPs expression and age or gender of patients were investigated to reflect the comorbidity factors in pan-cancer. The results showed that the expression trend of different IGFBPs in different cancers varies with age and gender. For example, IGFBP1 was highly expressed among elder patients in LGG, LUSC, while lowly expressed in CESC, COAD and UCEC (Fig. 3). And as is shown in Fig. 4, IGFBP1 was highly expressed in GBM and HNSC among male patients.

### Correlation between IGFBPs expression and prognosis in pan-cancer

**Fig. 5:**
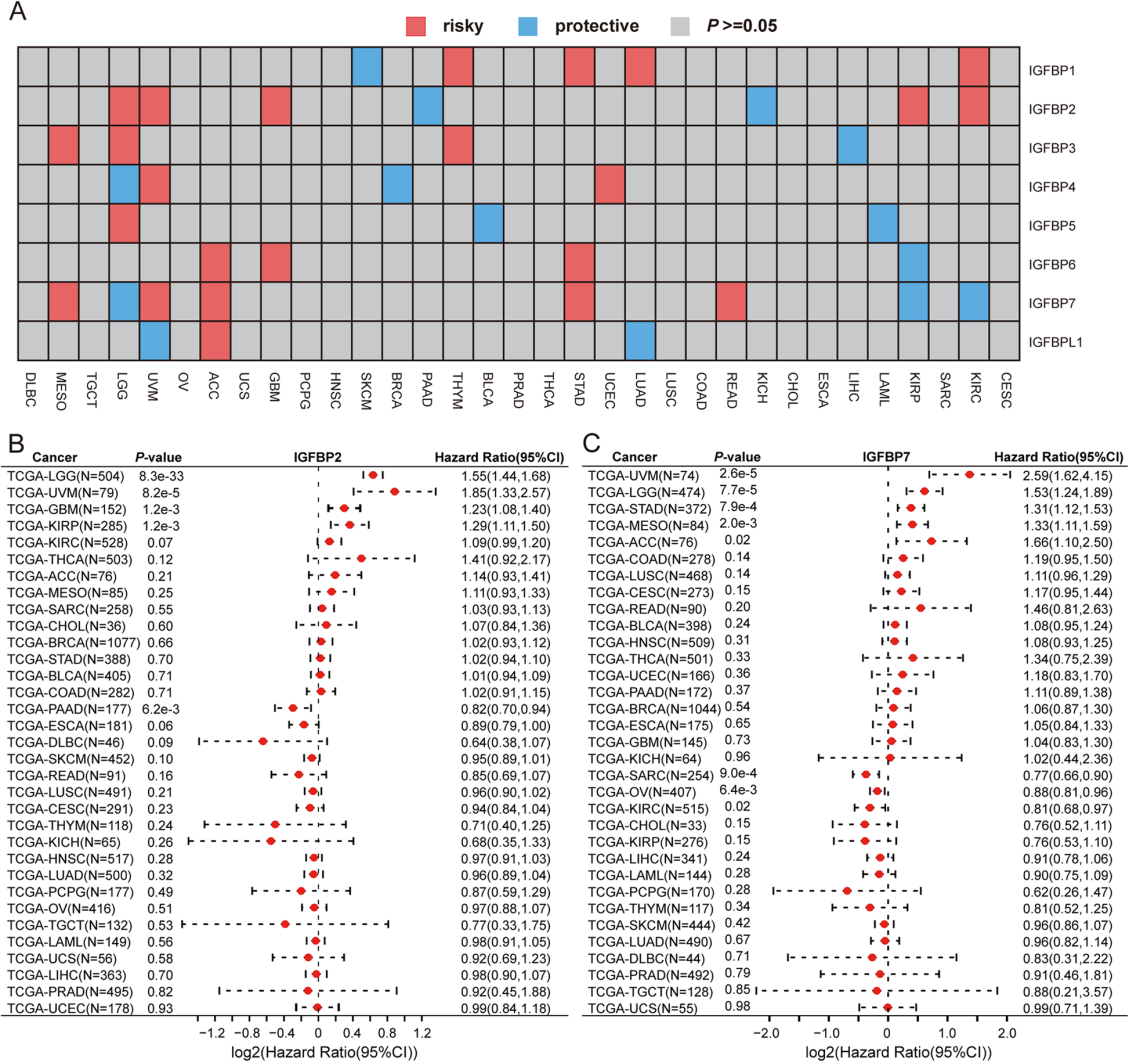
Prognostic significance of IGFBP genes. **A** Correlation between IGFBPs expression and survival of different cancers. Red color represents high risk of death whereas blue color represents low risk of death. **B**, **C** Forest plot for the prognostic analysis of IGFBP2 and IGFBP7 across 33 cancer types

The prognostic values of IGFBPs in pan-cancer were analyzed using the log-rank test. The results indicated that IGFBPs had different prognostic effects in 19 cancer types (Table 2, Fig. 5A and Additional file 2: Fig. S1). For example, in LGG, the expression of IGFBP2, IGFBP3 and IGFBP5 were related to poor prognosis, but the expression of IGFBP4 and IGFBP7 were related to good prognosis; and in UVM, the expression of IGFBP2, IGFBP4 and IGFBP7 were related to poor prognosis, but the expression of IGFBPL1 was related to good prognosis. In addition, IGFBP2 and IGFBP7 showed different prognostic roles in multiple cancer types, we therefore created forest plots to show specific predictive effects in various types of cancer (Fig. 5B and C).

**Table 2:** The association between IGFBPs expression and overall survival in pan-cancer

Gene	Cancer Type	Role	Gene	Cancer Type	Role
IGFBP1	SKCM	protective	IGFBP5	BLCA	protective
IGFBP1	KIRC	risky	IGFBP5	LAML	protective
IGFBP1	LUAD	risky	IGFBP5	LGG	risky
IGFBP1	STAD	risky	IGFBP5	UCEC	risky
IGFBP1	THYM	risky	IGFBP6	KIRP	protective
IGFBP2	KICH	protective	IGFBP6	ACC	risky
IGFBP2	PAAD	protective	IGFBP6	GBM	risky
IGFBP2	GBM	risky	IGFBP6	STAD	risky
IGFBP2	KIRC	risky	IGFBP7	KIRC	protective
IGFBP2	KIRP	risky	IGFBP7	KIRP	protective
IGFBP2	LGG	risky	IGFBP7	LGG	protective
IGFBP2	UVM	risky	IGFBP7	ACC	risky
IGFBP3	LIHC	protective	IGFBP7	MESO	risky
IGFBP3	LGG	risky	IGFBP7	READ	risky
IGFBP3	MESO	risky	IGFBP7	STAD	risky
IGFBP3	THYM	risky	IGFBP7	UVM	risky
IGFBP4	BRCA	protective	IGFBPL1	LUAD	protective
IGFBP4	LGG	protective	IGFBPL1	UVM	protective
IGFBP4	UVM	risky	IGFBPL1	ACC	risky

### Correlation between IGFBPs expression and cancer-related pathways and interaction between IGFBPs in pan-cancer

**Fig. 6:**
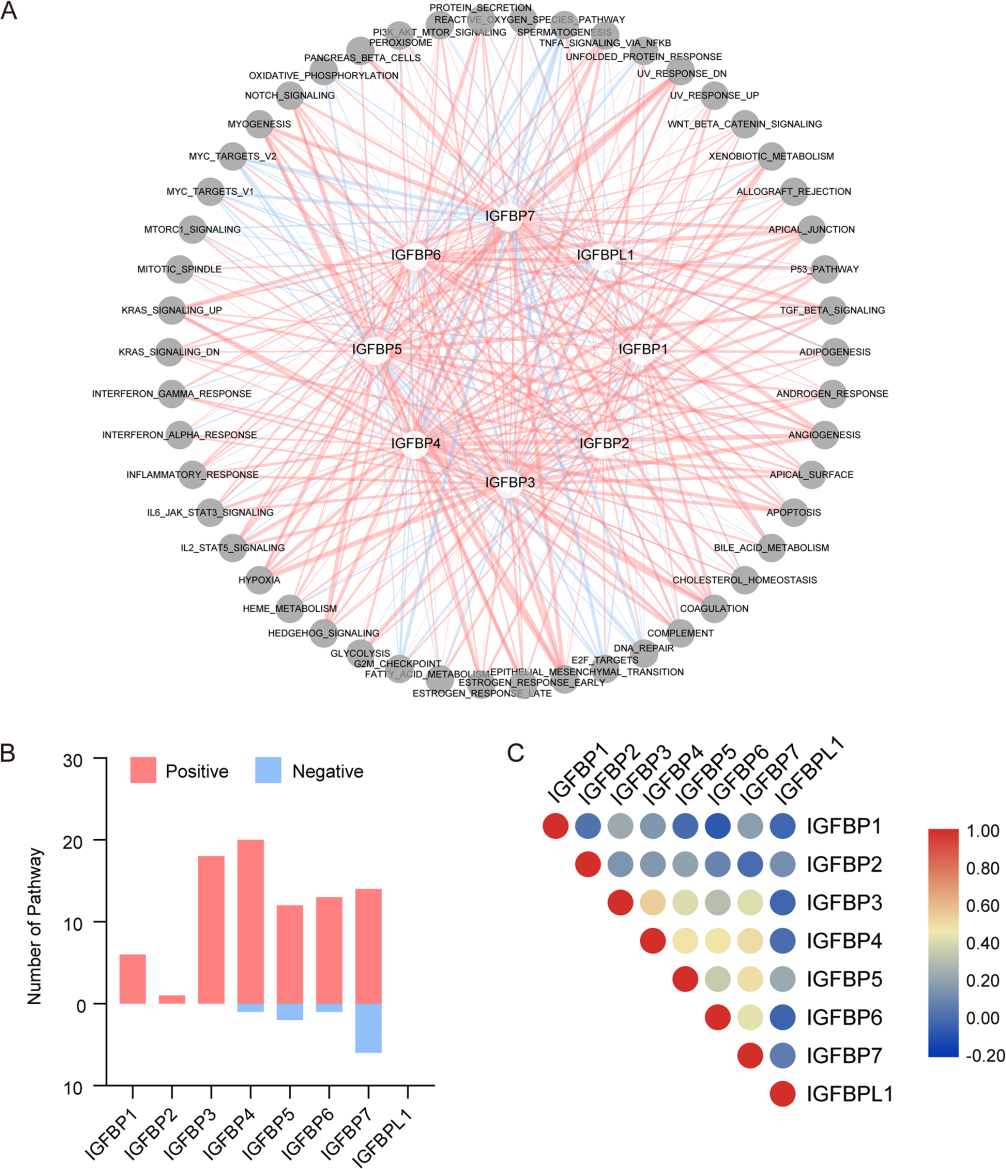
Association of IGFBPs expression with cancer-related pathways. **A** Network displaying the correlation between IGFBPs expression and cancer-related pathways. The white nodes represent IGFBPs and grey nodes represent pathways. The red edges represent activated pathways and blue edges represent inhibited pathways. **B** The number of significantly correlated pathways in each individual IGFBPs. Red color represents activated pathways and blue color represents inhibited pathways. **C** Correlation between the expression of different IGFBPs. Red color represents positive correlation and blue color represents negative correlation

We examined and illustrated the relationship between IGFBPs and hallmark pathways in order to clarify the molecular implications of IGFBPs in carcinogenesis. The results indicated that the expression of IGFBPs, especially IGFBP4 and IGFBP7, was significantly correlated with the suppression or activation of a variety of cancer pathways (Additional file 1: Table S4, Fig. 6A).

Among them, IGFBP1 mainly contributed to bile acid metabolism and xenobiotic metabolism pathways; IGFBP3 mainly participated in the angiogenesis, epithelial-mesenchymal transition, glycolysis, and hypoxia pathways; IGFBP4 mainly participated in angiogenesis, apoptosis, epithelial-mesenchymal transition, hypoxia, K-RAS signaling up, TGF-β signaling and ultraviolet response down pathways; IGFBP5 was mainly involved in angiogenesis, epithelial-mesenchymal transition, Hedgehog signaling, TGF-β signaling and ultraviolet response down pathways; IGFBP6 was mainly involved in the epithelial-mesenchymal transition pathway; IGFBP7 mainly participated in angiogenesis, E2F targets, epithelial-mesenchymal transition, Hedgehog signaling, K-RAS signaling up, MYC-targets-V2, TGF-β signaling and ultraviolet response down pathways. However, the expression of IGFBP2 and IGFBPL1 was not found to be significantly correlated with these signaling pathways. After summarizing the number of pathways that IGFBPs participated in, we found that IGFBPs activated much more pathways than they inhibited (Fig. 6B). In addition, IGFBPs may play synergistic roles in various cancer-related pathways because a significant correlation was detected (Fig. 6C), such as IGFBP3-IGFBP4(*r* = 0.54), IGFBP4-IGFBP7(*r* = 0.50) and IGFBP5-IGFBP7(*r* = 0.49).

**Fig. 7:**
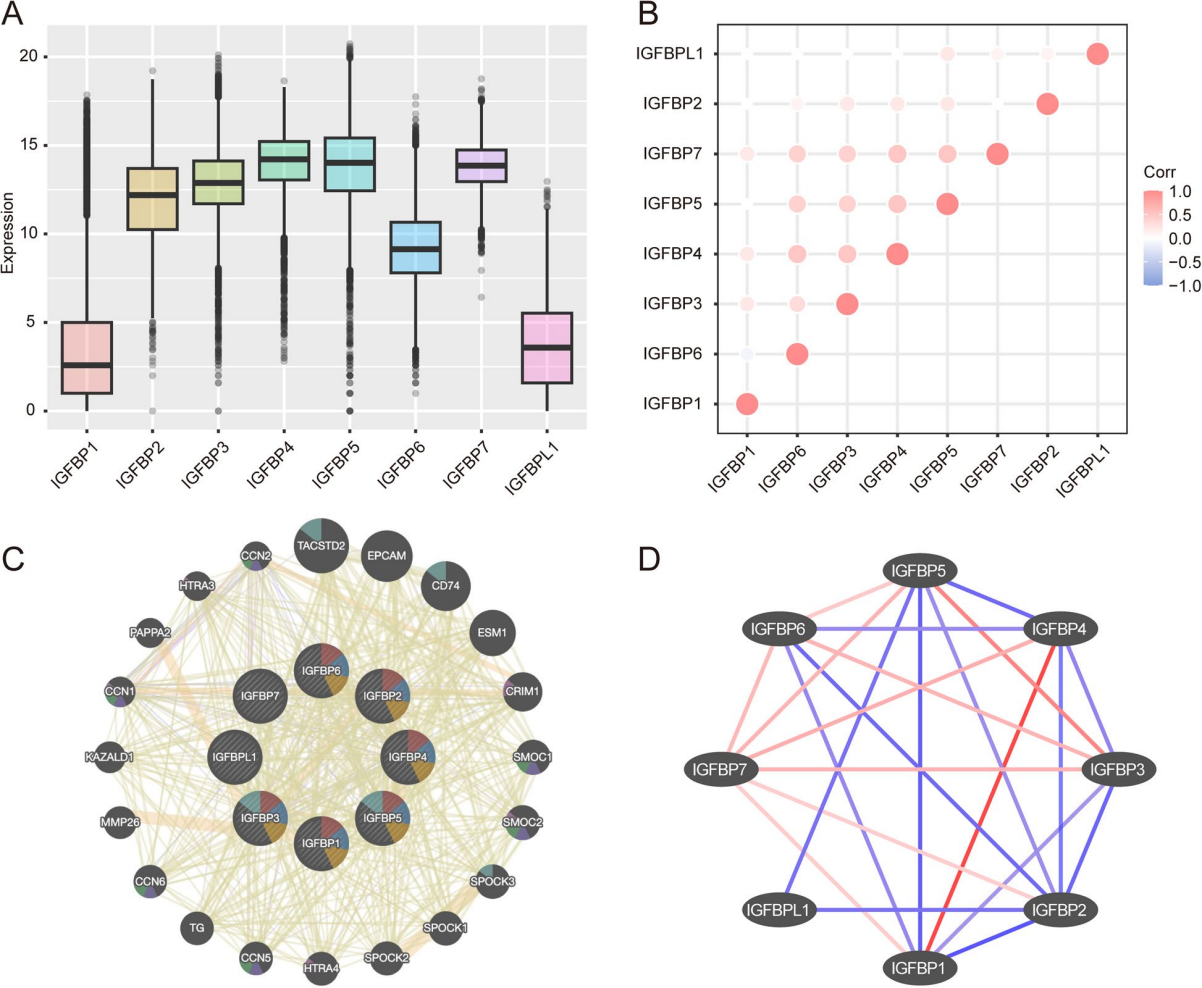
The correlation and interaction analysis among IGFBPs. **A** IGFBPs expression in all cancer patients in TCGA. **B** The correlation among IGFBPs expression in all 33 types of cancer from TCGA. Red color represents positive correlation and blue color represents negative color (*P* < 0.05). **C** Interaction network of IGFBPs from GENEMANIA. **D** Protein–protein interaction network of IGFBPs from STRING. Red color represents high degree score and blue color represents low degree score

**Fig. 8:**
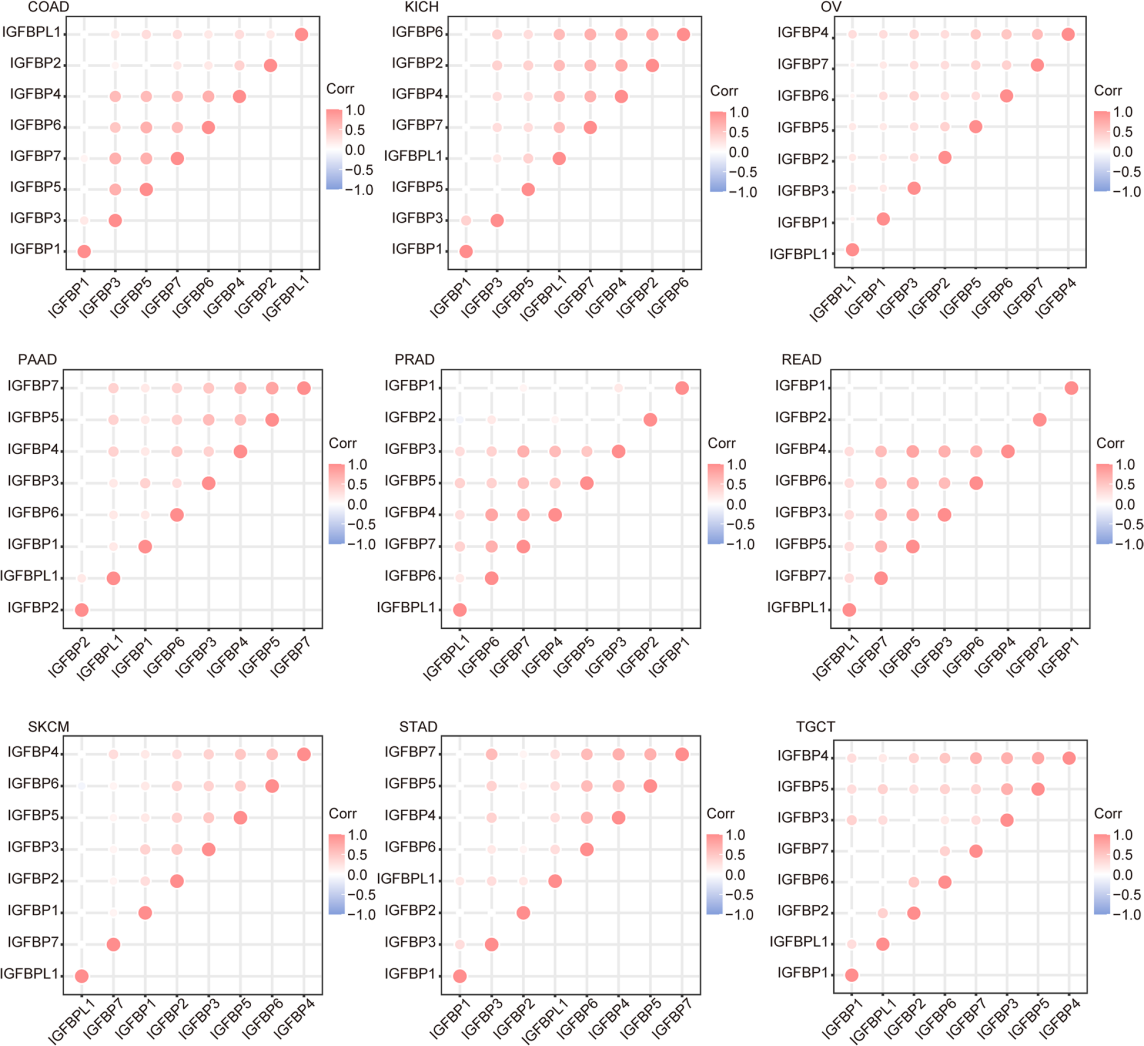
The correlation among IGFBPs expression in different 9 types of cancer from TCGA

We used the data from TCGA to analyze the correlation of IGFBPs expression in all cancer patients and 33 different cancers respectively. Figure 7A and B demonstrated the overall expression level and correlation of IGFBPs in pan-cancer. The results showed that, on the whole, the correlation between IGFBP3-6 was strong and mostly positive, such as IGFBP4-IGFBP7 (*r* = 0.50), IGFBP5-IGFBP7 (*r *= 0.50), while the correlation between IGFBP1, IGFBP2 or IGFBPL1 and other IGFBPs was poor (Fig. 7B). In addition, IGFBPs often showed positive correlation in different cancers (Fig. 8 and Additional file 2: Fig. S2). The interaction diagram established by GENEMANIA and STRING showed that there were extensive interactions between IGFBPs, especially IGFBP3, IGFBP5 and IGFBP7, while the interaction between IGFBPL1 and other IGFBPs was relatively poor (Fig. 7C and D).

### Correlation between IGFBPs expression and multiple molecular features in pan-cancer

#### Correlation between IGFBPs expression and TMB in pan-cancer

**Fig. 9:**
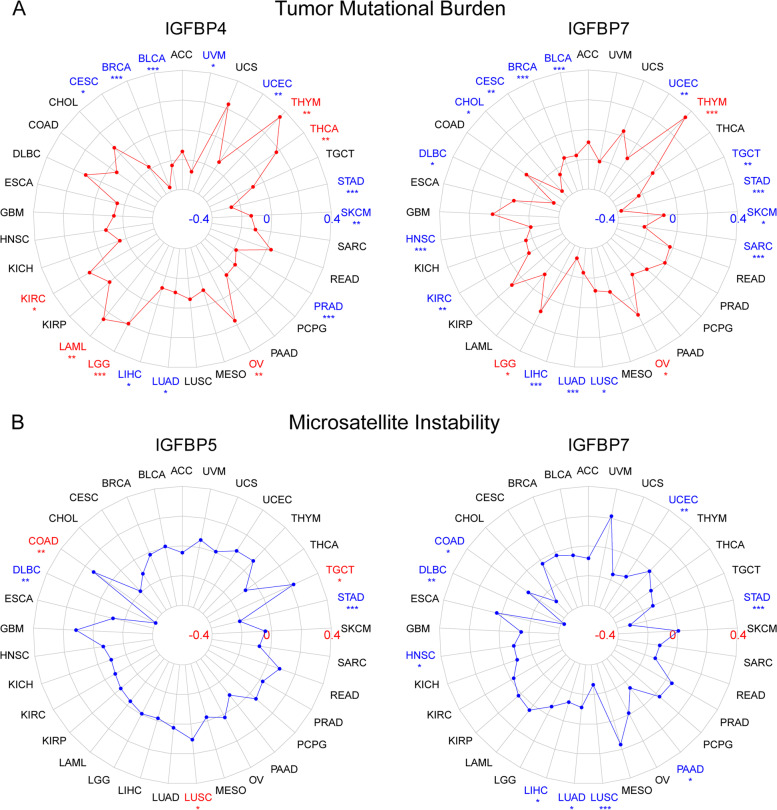
Correlation between IGFBPs expression and (**A**) tumor mutational burden (TMB) as well as (**B**) microsatellite instability (MSI) in pan-cancer. * *P* < 0.05, ** *P* < 0.01, *** *P* < 0.001

The TMB phenomenon, which is used to describe the mutation number involved in cancer cells, has emerged as a potential cancer prognosis biomarker in multiple tumor types. In the present study, the relationship between IGFBPs expression and TMB was investigated in 33 cancer types. Overall, except for IGFBP1, the expression of other IGFBPs showed significantly association with TMB in multiple cancers, especially IGFBP5 and IGFBP7, which were significantly correlated with TMB in 10 types of cancers, respectively, including LIHC, STAD, THYM, CESC, etc. Furthermore, IGFBPs were more negatively correlated with TMB in different cancers (Fig. 9A and Additional file 2: Fig. S3A).

#### Correlation between IGFBPs expression and MSI in pan-cancer

We also analyzed the association between IGFBPs expression and MSI in 33 cancer types. The results showed that, except for IGFBP1 and IGFBP2, the expression of other IGFBP genes was significantly correlated with MSI in several cancer types, and the vast majority also showed negative correlation. For example, IGFBP5 was negatively associated with MSI in TGCT, STAD as well as DLBC, and IGFBP7 was also negatively correlated with MSI in LUSC, STAD as well as DLBC (Fig. 9B and Additional file 2: Fig. S3B).

#### Correlation between IGFBPs expression and tumor stemness score in pan-cancer

**Fig. 10:**
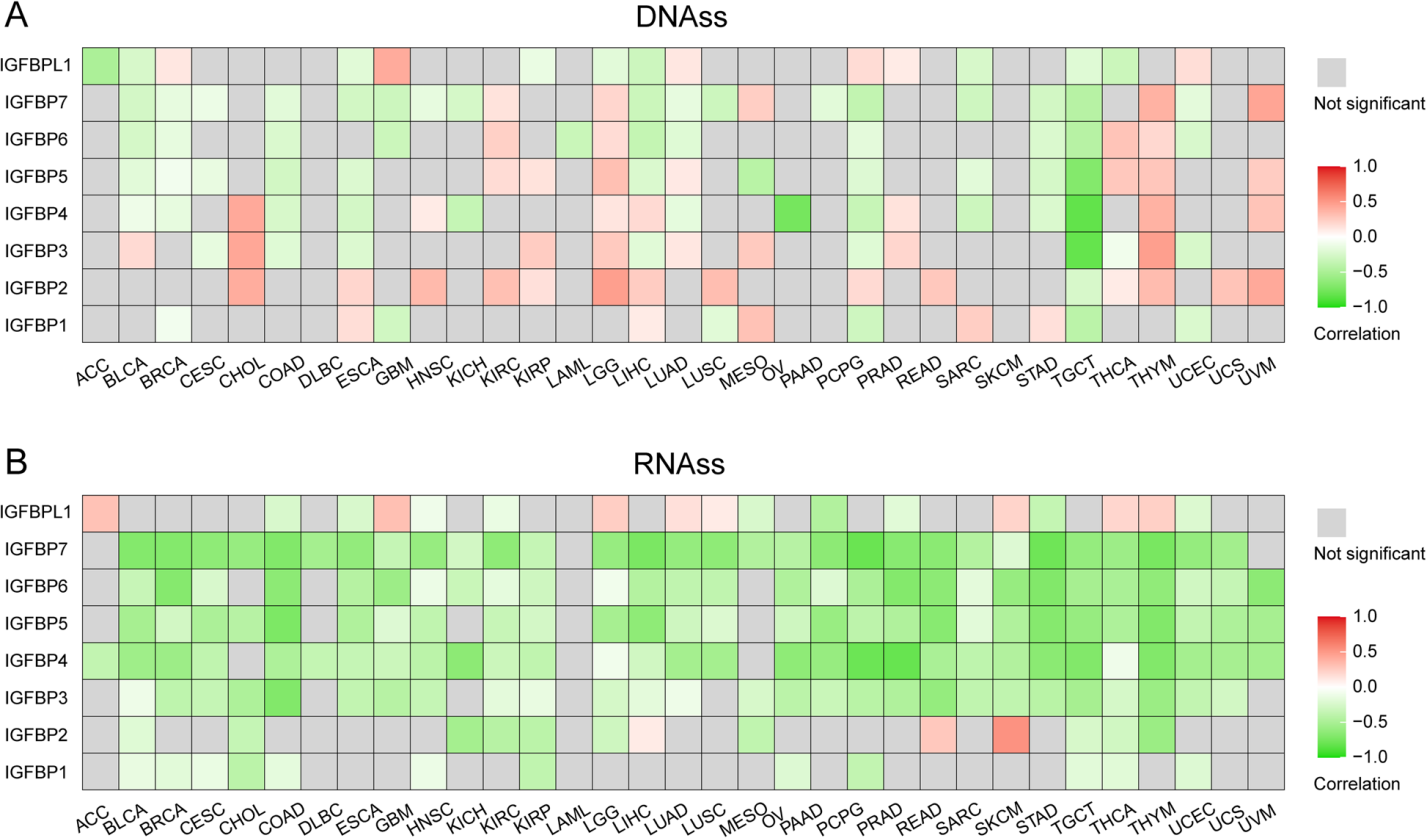
Correlation between IGFBPs expression and stemness score in pan-cancer. **A** Correlation between IGFBPs expression and DNAss. **B** Correlation between IGFBPs expression and RNAss. The red color represents positive correlations, whereas the green color represents negative correlations. The depth of the color represents the strength of the relationship. * *P* < 0.05, ** *P* < 0.01, *** *P* < 0.001. DNAss, DNA stemness score; RNAss, RNA stemness score

To evaluate the impact of IGFBPs on tumor stemness, we explored the relationship between IGFBPs expression and tumor stemness scores in 33 cancer types. The results showed that the expression of most IGFBPs was negatively associated with the DNAss (Fig. 10A) and RNAss (Fig. 10B). Especially for the RNAss, nearly all the correlations were negative in the majority of cancer types.

#### Correlation between IGFBPs expression and tumor immune microenvironment in pan-cancer

**Fig. 11:**
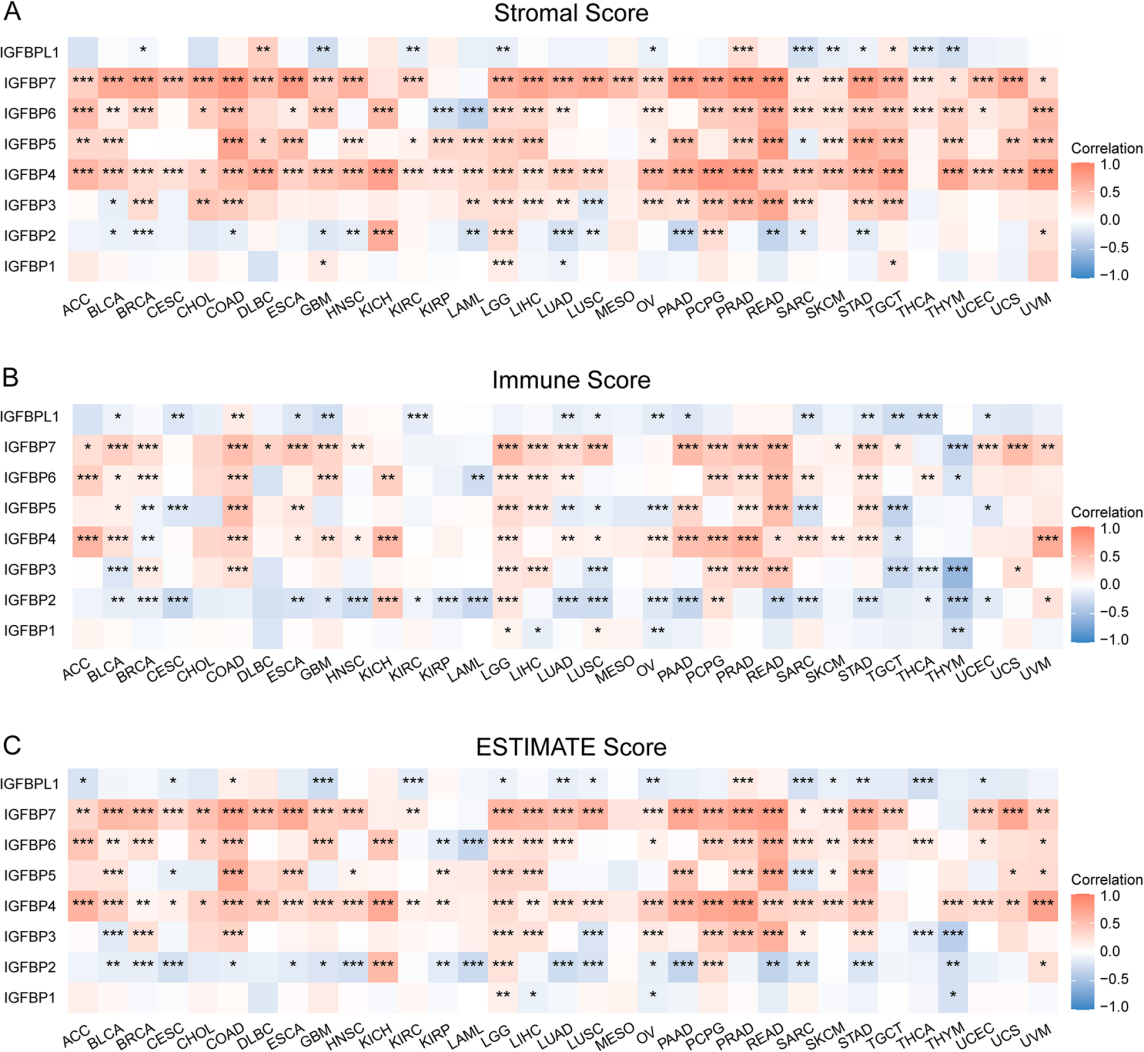
Correlation between IGFBPs expression and tumor immune microenvironment in pan-cancer. **A** Correlation between IGFBPs expression and stromal scores. **B** Correlation between IGFBPs expression and immune scores. **C** Correlation between IGFBPs expression and ESTIMATE scores. The red color represents positive correlations, whereas the blue color represents negative correlations. The depth of the color represents the strength of the relationship. * *P* < 0.05, ** *P* < 0.01, *** *P* < 0.001

We analyzed the correlation between the expression of IGFBPs and tumor immune microenvironment in 33 cancer types through the ESTIMATE algorithm. The results indicated that IGFBP family showed significantly negative or positive correlations with stromal (Fig. 11A), immune (Fig. 11B) and ESTIMATE (Fig. 11C) scores in pan-cancer. And for the majority of the cancers, IGFBP3, IGFBP4, IGFBP5, IGFBP6 and IGFBP7 were significantly positively associated with stromal, immune as well as ESTIMATE scores including COAD, PCPG, PRAD, READ and STAD. While for IGFBP1, IGFBP2 and IGFBPL1, there were more negative correlations than negative.

#### Correlation between IGFBPs expression and immune cell infiltration in pan-cancer

**Fig. 12:**
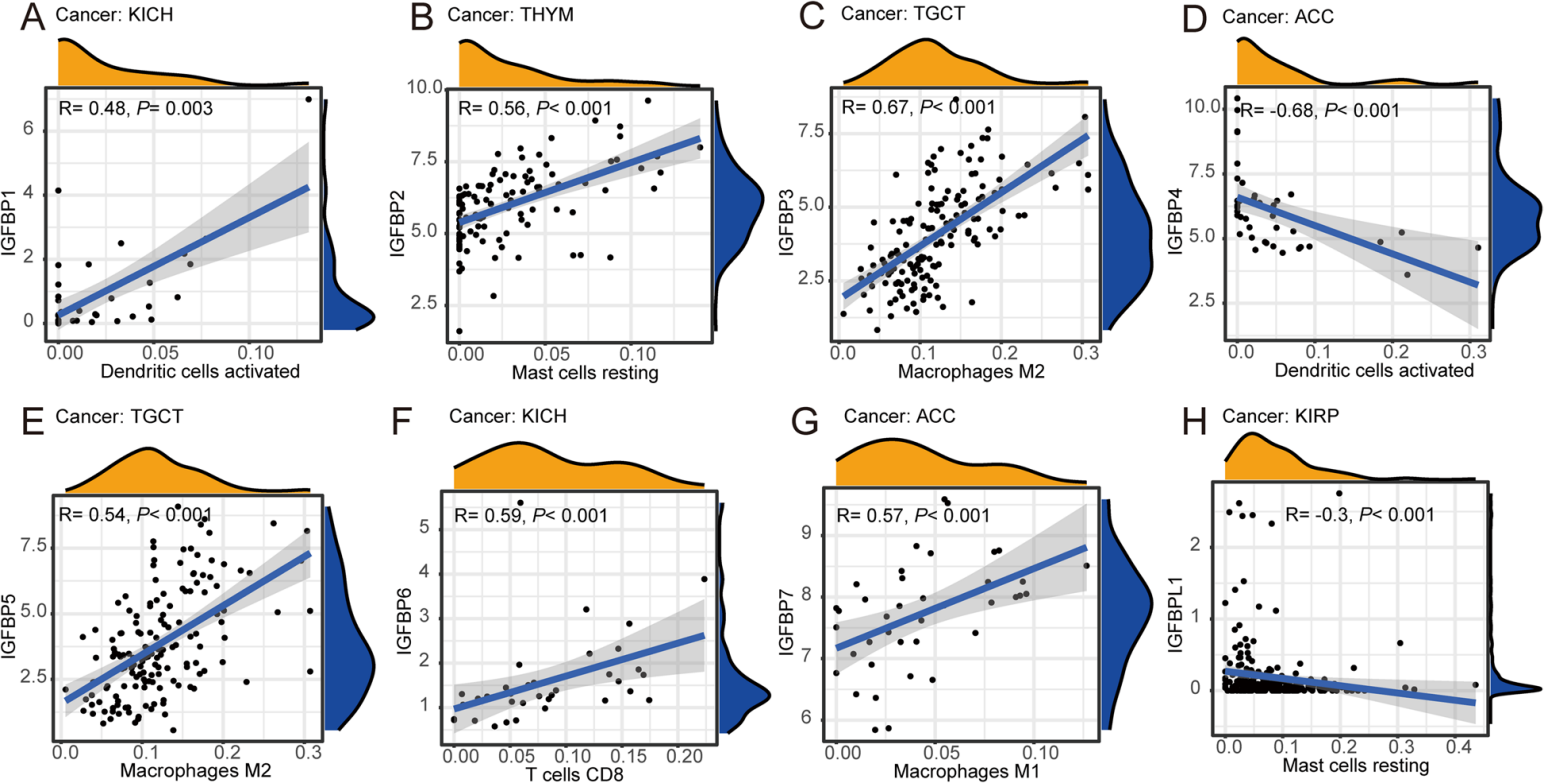
Correlation between IGFBPs expression and immune cell infiltration. **A** Correlation between IGFBP1 expression and Dendritic cells activated infiltration level in KICH. **B** Correlation between IGFBP2 expression and Mast cells resting cell infiltration level in THYM. **C** Correlation between IGFBP3 expression and Macrophages M2 cell infiltration level in TGCT. **D** Correlation between IGFBP4 expression and Dendritic cells activated cell infiltration level in ACC. **E** Correlation between IGFBP5 expression and Macrophages M2 cell infiltration level in TGCT. **F** Correlation between IGFBP6 expression and T cells CD8 cell infiltration level in KICH. **G** Correlation between IGFBP7 expression and Macrophages M1 cell infiltration level in ACC. **H** Correlation between IGFBPL1 expression and Mast cells resting cell infiltration level in KIRP

We also analyzed the association between IGFBPs expression and 22 types of immune cell infiltration levels in 33 cancer types. The results indicated that in 26 kinds of cancers (including ACC, renal chromophobe cell carcinoma, thymic carcinoma, uterine SARC, and CHOL, etc.), the expression of IGFBPs was significantly associated with different immune cell infiltration, such as resting mast cells and macrophages M2 cells and naïve B cells (Table 3 and Additional file 1: Table S5). Figure 12 shows the correlation between IGFBPs expression and infiltration levels of specific immune cells in various cancer types.

**Table 3:** Correlation between IGFBPs expression and immune cell infiltration in Pan-cancer

Gene	Cancer Type	Cell Type	Correlation Coefficient	*P* value
IGFBP1	CHOL	T cells CD8	-0.46	0.007
IGFBP1	ACC	T cells CD4 memory resting	-0.44	0.004
IGFBP1	CHOL	Plasma cells	-0.42	0.015
IGFBP1	UCS	Neutrophils	0.41	0.009
IGFBP1	KICH	Dendritic cells activated	0.48	0.003
IGFBP2	UVM	Plasma cells	-0.64	< 0.001
IGFBP2	CHOL	NK cells activated	-0.53	0.002
IGFBP2	THYM	Mast cells resting	0.56	< 0.001
IGFBP2	MESO	Mast cells resting	-0.42	< 0.001
IGFBP2	KICH	Macrophages M2	0.44	0.008
IGFBP2	LGG	Macrophages M1	0.4	< 0.001
IGFBP2	KICH	Macrophages M0	0.51	0.002
IGFBP2	DLBC	Macrophages M0	0.46	0.001
IGFBP2	CHOL	B cells naive	0.4	0.021
IGFBP2	UVM	B cells naive	-0.43	0.012
IGFBP2	CHOL	B cells memory	-0.42	0.016
IGFBP3	TGCT	T cells CD4 memory activated	-0.45	< 0.001
IGFBP3	CHOL	NK cells activated	-0.48	0.005
IGFBP3	UCS	Neutrophils	0.51	< 0.001
IGFBP3	TGCT	Macrophages M2	0.67	< 0.001
IGFBP3	THYM	Macrophages M1	0.48	< 0.001
IGFBP3	ACC	B cells naive	-0.4	0.010
IGFBP3	TGCT	B cells naive	-0.65	< 0.001
IGFBP6	CHOL	T cells CD4 memory activated	0.4	0.023
IGFBP6	UVM	T cells CD4 memory activated	-0.4	0.020
IGFBP7	PCPG	Mast cells resting	0.4	< 0.001
IGFBP7	UCS	Macrophages M0	-0.4	0.012
IGFBP7	UVM	B cells naive	-0.4	0.020

### Genetic variation characteristics of IGFBPs in pan-cancer

**Fig. 13:**
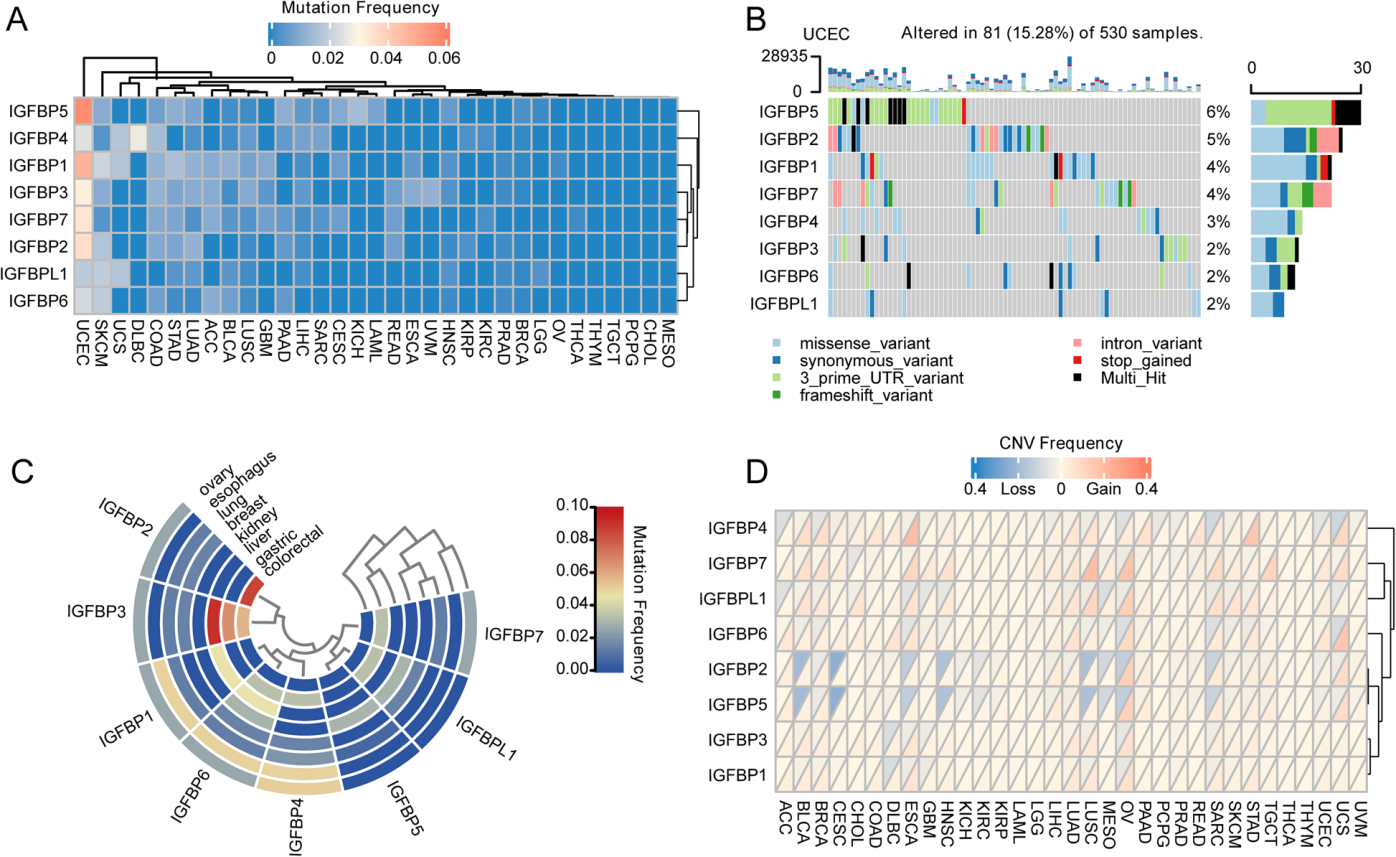
Genetic alternations of IGFBPs in pan-cancer. **A** Mutation frequency of IGFBPs in different cancers. Red color represents high mutation frequency whereas blue color represents low mutation frequency. **B** Oncoplot for IGFBPs in UCEC. IGFBP5 shows the most frequent mutation in UCEC. **C** Mutation frequency of IGFBPs in different cancer cell lines (colorectal, gastric, liver, kidney, breast, lung, esophagus and ovary) from CCLE database. Red color represents high mutation frequency whereas blue color represents low mutation frequency. **D** The copy number variations frequency of IGFBPs in different cancers. The upper part of each grid shows the deletion frequency, and the bottom part shows the amplification frequency

We explored the mutation frequency of the IGFBP family in 33 cancer types (Fig. 13A). The overall average mutation rate of IGFBPs was 0–5.7%, the mutation frequency of IGFBPs was less than 2% in most tumors except for UCEC. IGFBP1 and IGFBP5 showed relatively higher mutation frequency, while IGFBPL1 showed the lowest mutation frequency. UCEC exhibited frequent mutations of all IGFBPs genes, the mutational details were visualized via a waterfall plot (Fig. 13B). In contrast, no IGFBPs mutations were found in CHOL, THYM and PCPG. And the mutation types involved in case of the genetic variation of IGFBPs in all cancers were displayed in Additional file 2: Fig. S4. Furthermore, we also summarized the mutational status of IGFBPs in eight cancer cell lines from CCLE (Fig. 13C). The results revealed that IGFBPs mutations were more likely to occur in gastric, lung, and ovarian cancer cell lines.

Furthermore, the copy number variations (CNVs) of IGFBPs in pan-cancer was also analyzed, the results showed that the IGFBP family had a low degree of CNVs in various cancers (Fig. 13D). Relatively speaking, IGFBP4, IGFBP7 and IGFBPL1 showed more amplifications of copy number in multiple cancer types. While IGFBP2 and IGFBP5 had higher frequencies of copy number deletions.

### Correlation between IGFBPs expression and IGFBPs variation in pan-cancer

The correlation between IGFBPs expression and IGFBPs mutation, CNVs was analyzed to investigate whether the IGFBPs variation modulated their expression levels. The results showed that the expression of IGFBP3 was modulated by its mutation in UCEC, and the expression of IGFBP6 was regulated by its mutation in SKCM. Additionally, we found that CNVs of IGFBPs could affect their expression in multiple cancer types (Table 4). For instance, in BRCA and OV, the increased expression of IGFBP1 and IGFBP2 was correlated with its increased copy number. The copy number variations of other IGFBPs also have different impacts on their expression in corresponding cancers, the details were displayed in Additional file 1: Table S6.

**Table 4:** Correlation between IGFBPs CNV and IGFBPs expression

Gene	Cancer type	CNV	n	Expression median value	*P* value
IGFBP1	BRCA	DEL	19	0(0–1.585)	0.045
		GAIN	34	2(1–2.896)	
		No Change	1037	1.585(0–2.585)	
	OV	DEL	18	2.5(1.146–3.387)	0.045
		GAIN	30	3.522(2.08–4.985)	
		No Change	329	3.459(2.322–4.807)	
IGFBP2	BRCA	DEL	48	11.881(10.332–12.928)	0.003
		GAIN	35	12.49(11.017–13.376)	
		No Change	1007	12.726(11.625–13.491)	
	CESC	DEL	71	11.064(9.844–12.181)	< 0.001
		GAIN	3	8.43(8.36–12.257)	
		No Change	220	12.548(10.626–13.705)	
	OV	DEL	55	14.78(14.242–15.876)	0.011
		GAIN	52	15.349(14.951–16.69)	
		No Change	270	15.583(14.609–16.21)	
	PAAD	DEL	3	10.011(9.76–11.867)	0.016
		GAIN	6	13.997(13.391–14.811)	
		No Change	168	12.227(11.325–13.006)	
	PRAD	DEL	9	12.611(12.287–12.931)	< 0.001
		GAIN	5	13.896(12.477–14.945)	
		No Change	482	13.81(13.258–14.313)	
	UCEC	DEL	15	13.56(12.422–14.836)	0.007
		GAIN	9	15.199(15.088–15.404)	
		No Change	516	13.28(11.9–14.435)	

### Verification of IGFBPs expression at mRNA and protein levels

#### Validation of the mRNA expression levels of IGFBPs by qRT-PCR

**Fig. 14:**
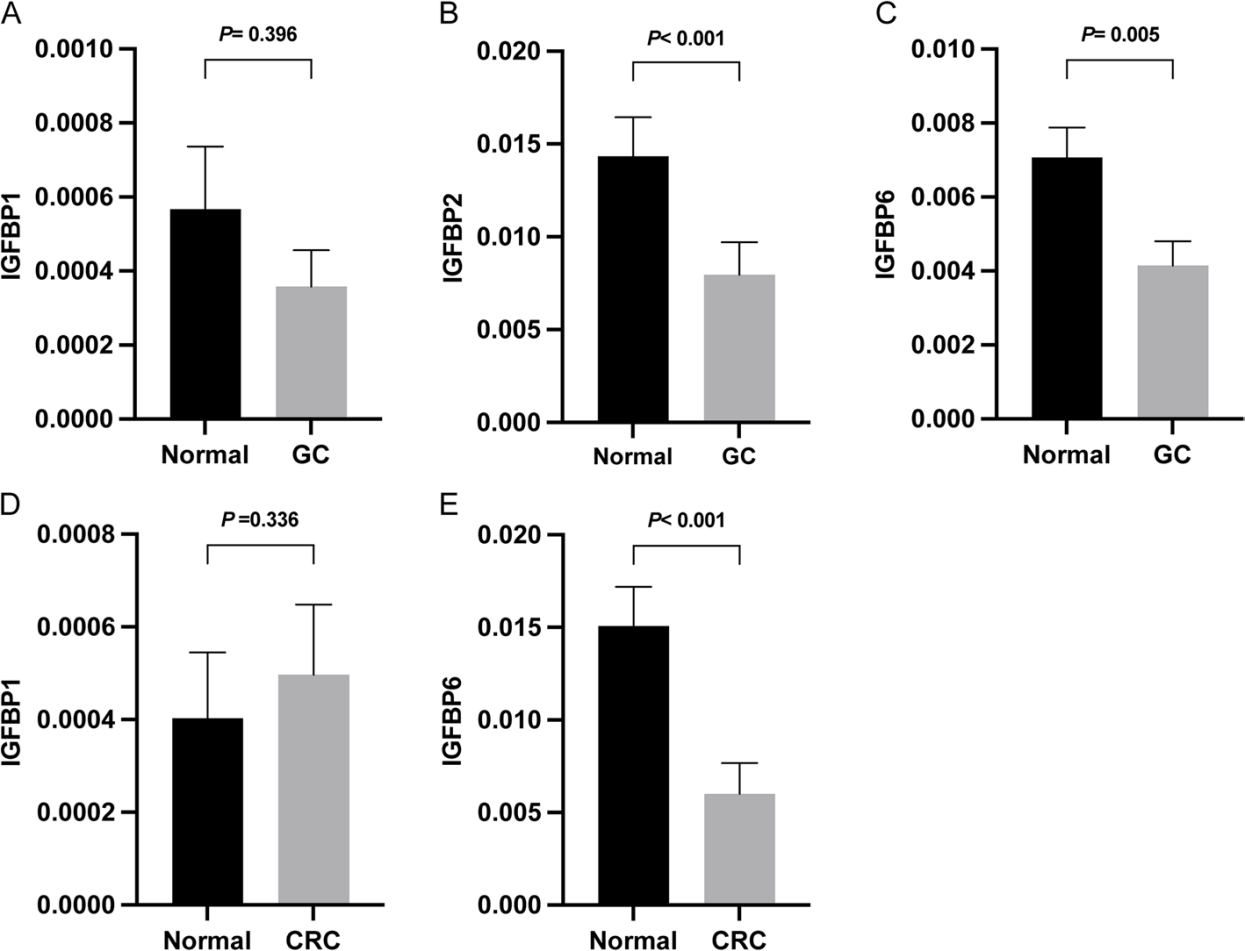
Validation of the mRNA expression levels of IGFBPs using the qRT-PCR experiments. *P* < 0.05 was considered statistically significant. **A** The relative expression of IGFBP1 in gastric cancer. **B** The relative expression of IGFBP2 in gastric cancer. **C** The relative expression of IGFBP6 in gastric cancer. **D** The relative expression of IGFBP1 in colorectal cancer. **E** The relative expression of IGFBP6 in colorectal cancer

In order to verify the analysis results of TCGA at the mRNA level, we selected the differentially expressed IGFBPs in some tumor tissues and then performed qRT-PCR experiments to verify the expression of IGFBP1, IGFBP2, and IGFBP6 in 67 pairs of gastric cancer and adjacent tissues, as well as 44 pairs of colorectal cancer and adjacent tissues. The results indicated that, consistent with the results of TCGA analysis, the expression of IGFBP2 (*P* < 0.001) and IGFBP6 (*P* = 0.005) in gastric cancer tissues was lower than that in normal gastric tissues; the expression of IGFBP6 (*P* < 0.001) in colorectal cancer tissues was also lower than that in normal colorectal tissues (Fig. 14). Besides, the verification results of IGFBP1 in gastric cancer and colorectal cancer did not reach statistical difference, but the expression trend in colorectal cancer was consistent with the analysis results of TCGA.

#### Validation of the protein expression levels of IGFBPs by immunohistochemistry

**Fig. 15:**
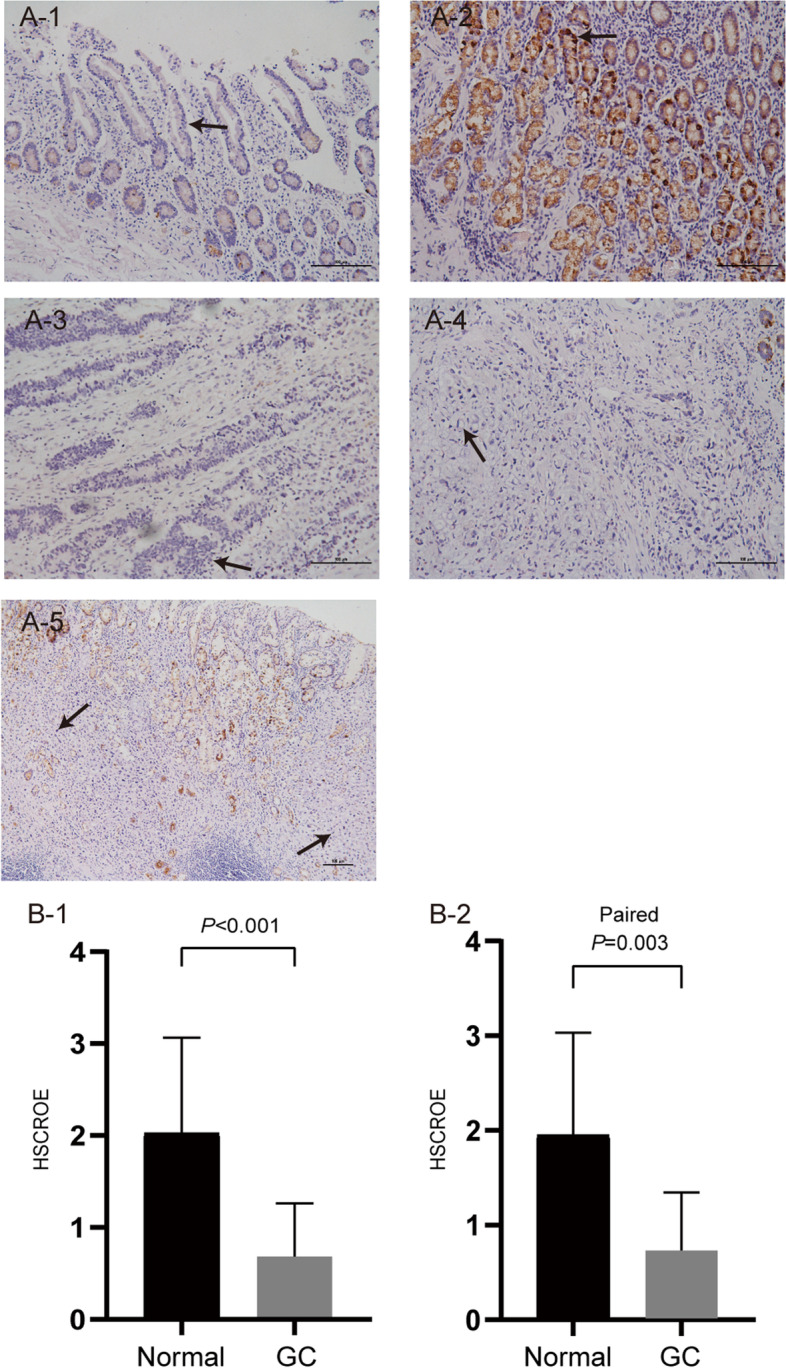
The expression levels of IGFBP2 protein in gastric cancer tissues and adjacent normal tissues. **A** The expression levels of IGFBP2 protein in gastric tissues by IHC staining: (A-1) Gastric pit tissue (200x), the arrow indicates the negative staining of IGFBP2 protein in gastric pit cell. (A-2) Normal gastric antrum tissue (200x), the arrow indicates the positive staining of IGFBP2 protein in gastric antrum cell. (A-3) Moderately differentiated GC tissue (200x), the arrow indicates the negative staining of IGFBP2 protein in GC cell. (A-4) Gastric signet ring cells carcinoma (200x), the arrow indicates the negative staining of IGFBP2 protein in signet ring cell. (A-5) GC tissue and adjacent normal gastric tissue (100x), the solid arrows indicate the negative staining of IGFBP2 protein in GC cells, and the dotted arrow indicates the positive staining of IGFBP2 protein in normal gastric cell. **B** Statistical quantification of the HSCORE of IGFBP2 staining in the indicated tissues: (B-1) The protein expression level of IGFBP2 in adjacent normal gastric tissues was significantly higher than that in GC tissues. (B-2) In the paired GC tissues and adjacent normal tissues, the protein expression of IGFBP2 in the normal tissues was significantly higher. GC, gastric cancer; HSCORE, histological score

Based on the results of the qPCR verification and considering the lack of IGFBP2 protein expression data in the Human Protein Atlas (HPA), we performed IHC staining to verify the protein expression of IGFBP2 in gastric cancer tissues. The results showed that IGFBP2 had no obvious staining in the pits of the normal stomach (Fig. 15A-1) but was labeled dark brown in the normal gastric antrum glands (Fig. 15A-2). Besides, there was no obvious positive staining on the moderately differentiated adenocarcinoma (Fig. 15A-3) and signet ring cell carcinoma (Fig. 15A-4) of the stomach. Figure 15A-5 shows the staining of continuous gastric cancer tissues and adjacent normal tissues. From a quantitative point of view, IGFBP2 protein expression was lower in gastric cancer tissues compared with in normal tissues (*P* < 0.001) (Fig. 15B-1). The expression of IGFBP2 in matched normal tissues is higher than that in gastric cancer tissues (Fig. 15B-2).

### Frontier trend analysis of IGFBP related research from 2000 to 2021

**Fig. 16:**
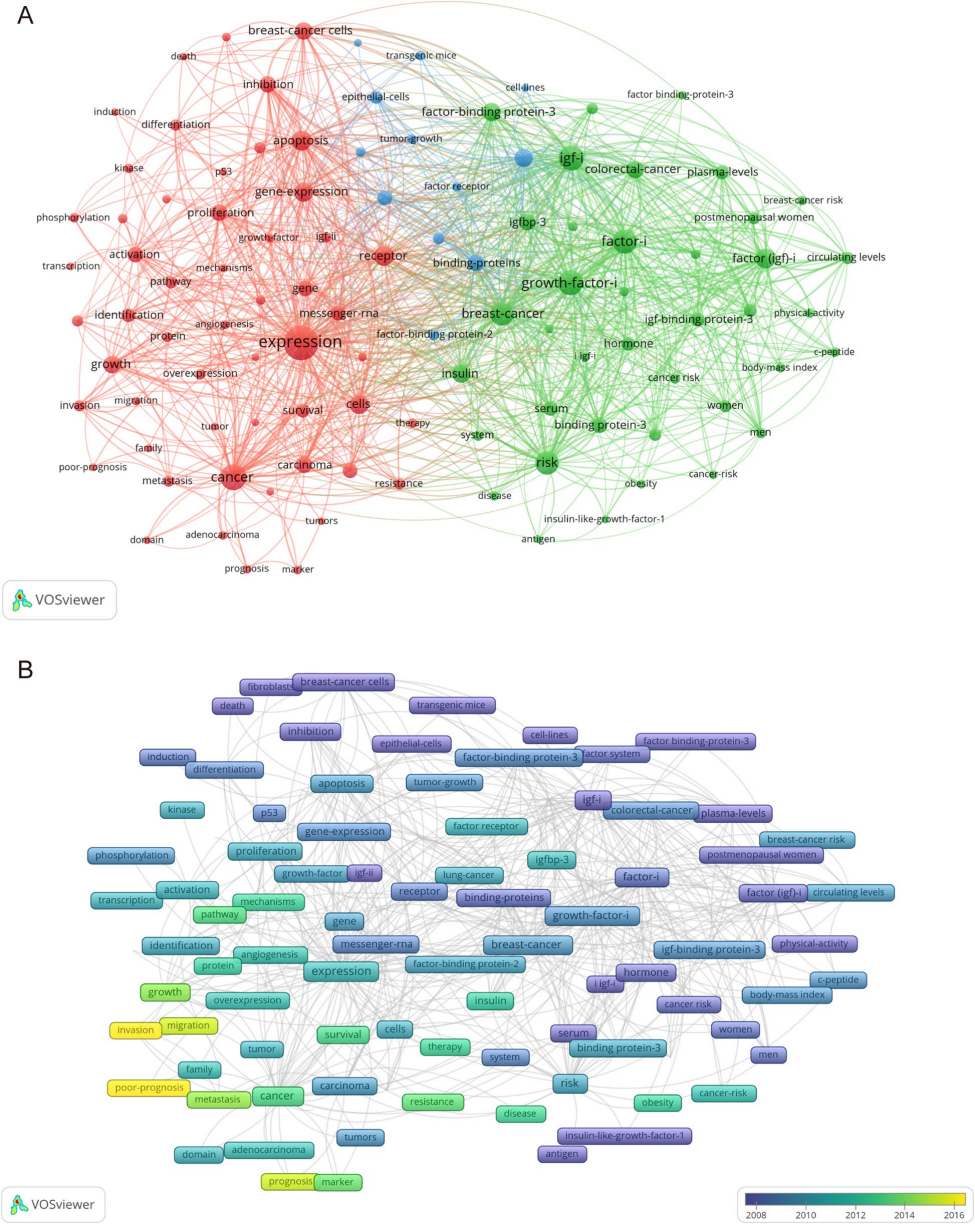
Keywords co-occurrence analysis. **A** The co-occurrence network visualization map of the top 104 keywords. The visualization map of publications for 104 keywords forming 3 collaborating clusters (nodes with the same color); a node represents a keyword, the size of the node represents the number of publications, a link shows collaboration, and the distance and the thickness of the link between nodes show the relative strength of the relation. **B** The co-occurrence overlay visualization map of the top 104 keywords. The color of node represents the average publication year of the keywords, and the blue to yellow represents the average publication year from early to late

From 2000 to 2021, 3543 studies fulfilled the search criteria in total. Overall, the publication of related researches peaked at 224 articles in the year 2007 and then leveled off, while the annual citation showed an increasing trend, reaching a peak of 9514 times in year 2021. Co-occurrence analysis of keywords showed that a total of 104 keywords appeared more than 40 times (Table 5). All the keywords were divided into 3 clusters, with the following occurring more frequently: expression, risk, apoptosis, proliferation, survival, growth, breast cancer, prostate cancer, colorectal cancer, lung cancer, etc. The above keywords appeared in the center of each cluster, and had higher weights in terms of size and strength of relationship compared with other keywords (Fig. 16A). The most recent keywords of IGFBP cancer-related studies were as follows: invasion, poor-prognosis, migration, prognosis, biomarker, target, survival, and therapy, etc. (Fig. 16B).

**Table 5:** Clusters of the top 104 keywords

Cluster	Keywords	Counts	Rank	Cluster	Keywords	Counts	Rank
1	expression	758	1	2	risk	356	7
1	cancer	434	2	2	factor (igf)-i	261	10
1	gene-expression	245	11	2	insulin	216	14
1	cells	235	12	2	colorectal-cancer	190	18
1	growth	194	17	2	igfbp-3	174	22
1	proliferation	185	19	2	binding protein-3	162	25
1	carcinoma	172	23	2	serum	141	29
1	gene	164	24	2	igf-binding protein-3	133	31
1	activation	155	26	2	hormone	128	33
1	messenger-rna	153	27	2	plasma-levels	128	35
1	progression	138	30	2	serum-levels	110	36
1	survival	130	32	2	association	102	38
1	identification	128	34	2	postmenopausal women	97	40
1	pathway	100	39	2	women	97	41
1	lung-cancer	90	43	2	circulating levels	95	42
1	metastasis	89	44	2	factor system	83	49
1	differentiation	88	45	2	men	79	52
1	resistance	88	46	2	system	75	53
1	invasion	86	47	2	cancer risk	70	56
1	binding	85	48	2	colon-cancer	67	59
1	cancer cells	81	50	2	body-mass index	66	60
1	igf-ii	79	51	2	c-peptide	65	61
1	overexpression	74	54	2	disease	64	62
1	protein	71	55	2	physical-activity	64	64
1	phosphorylation	64	63	2	breast-cancer risk	61	66
1	down-regulation	60	68	2	factor-binding-proteins	58	72
1	kinase	60	69	2	prostate-cancer risk	58	73
1	therapy	60	70	2	i igf-i	57	75
1	in-vitro	59	71	2	cancer-risk	56	76
1	cancer-cells	57	74	2	obesity	54	79
1	cell-proliferation	56	77	2	factor binding-protein-3	52	81
1	adenocarcinoma	54	78	2	estrogen	50	83
1	marker	53	80	2	antigen	47	88
1	growth-factor	52	82	2	insulin-like-growth-factor-1	43	95
1	angiogenesis	49	84	3	receptor	266	8
1	migration	49	86	3	apoptosis	261	9
1	prognosis	48	87	3	prostate-cancer	220	13
1	family	45	91	3	factor-binding protein-3	215	15
1	tumors	45	92	3	breast-cancer cells	197	16
1	mechanisms	44	94	3	binding-proteins	182	20
1	proteins	43	96	3	inhibition	175	21
1	tumor	43	97	3	factor-i receptor	142	28
1	domain	41	98	3	epithelial-cells	108	37
1	growth-factor-ii	41	99	3	factor receptor	68	57
1	poor-prognosis	41	100	3	tumor-growth	68	58
1	transcription	41	101	3	transgenic mice	63	65
1	hepatocellular-carcinoma	40	103	3	signaling pathways	61	67
1	p53	40	104	3	fibroblasts	49	85
2	growth-factor-i	425	3	3	induction	46	89
2	igf-i	410	4	3	death	45	90
2	factor-i	397	5	3	cell-lines	44	93
2	breast-cancer	370	6	3	carcinoma cells	40	102

## Discussion

IGF is one of the important somatomedins in humans, which plays an important role in regulating the proliferation, differentiation and apoptosis of tissue cells, the growth and development of the body and the development of tumors [27]. The IGF system is commonly found in human circulation and various target tissues, and its main components include IGF ligands (IGF-I, IGF-II and insulin), receptors (IGF-IR, IGF-IIR and insulin receptor) and IGFBPs [28]. The IGFBPs mainly include IGFBP1-6 with high affinity for IGF and IGFBP7 and IGFBPL1 with low binding to IGF. In addition to the function of binding IGF, each IGFBP has its own unique role. In the human circulation, IGFBPs function primarily by binding to IGF-I/II to form complexes, thereby prolonging the half-life of IGFs and regulating their clearance, as well as controlling the transport of IGFs in plasma and the diffusion or efflux of IGFs in the vasculature [29]. In tissues, IGFBPs can enhance the effect of IGFs by directly interacting with cell surface or extracellular matrix to localize IGFs near their receptors and regulate their binding to receptors, or they can inhibit the action of IGFs by limiting their receptor access [30]. In recent years, it has been found that IGFBPs can act outside tumor cells, in the cytoplasm or in the nucleus, and play different functions on the proliferation and apoptosis of tumor cells under different conditions [5]. Several studies have found that circulating IGFBPs concentrations or tissue mRNA expression levels are associated with the risk or prognosis of certain cancers. The special properties of IGFBPs make them closely related to the complex signaling pathways and various molecular features of tumors. The role of IGFBPs in the carcinogenesis and development of tumors is increasingly attracting attention.

In the present study, we utilized multi-source data to characterize the expression profiles of IGFBP gene family in pan-cancer, and its relationship with prognosis, cancer-related pathways, TMB, MSI, tumor stemness, tumor immune microenvironment, comorbidity factors, etc. The results of the database analysis were validated using immunohistochemistry and qRT-PCR. Our study indicates that IGFBPs are differentially expressed in multiple cancers and affect diverse tumor prognosis. The expression of IGFBPs is correlated with multiple cancer hallmark pathways and molecular features of tumors. On this basis, we conducted a comprehensive bibliometric analysis and literature review on the role of IGFBP family in the occurrence and development of pan-cancer. As far as we know, our study is the first “quantitative analysis” based systematic review supported by bioinformatics and bibliometrics to provide a pan-cancer wide characterization about IGFBP gene family in tumorigenesis and progression. These findings provide new clues for identifying novel biomarkers and therapeutic targets for cancer diagnosis and treatment.

### IGFBP gene family was differentially expressed in various cancers, which makes IGFBPs available as new cancer biomarkers and therapeutic targets

The current study extracted multi-level data from TCGA, Oncomine, HPA, and CCLE to analyze the expression profiles of IGFBPs at the mRNA, protein, and cell levels in 33 tumors. Our examination of the TCGA data and verification using the Oncomine database revealed that the expression of IGFBPs at the mRNA level varies in multiple cancer types. In stomach adenocarcinoma, IGFBP1 is upregulated, but IGFBP2 and IGFBP6 are downregulated. In COAD, IGFBP1 is significantly highly expressed, while the expression of IGFBP6 is low. In LIHC, the expression of IGFBP1, IGFBP2, IGFBP3, IGFBP4 are all low. Besides, the qRT-PCR experiment confirmed the low expression of IGFBP2 and IGFBP6 in gastric cancer and the low expression of IGFBP6 in colorectal cancer. According to HPA immunohistochemistry findings, IGFBP staining intensity in 20 types of tumor tissues was primarily low or medium at the protein expression level. Besides, the IHC staining further confirmed the low expression of IGFBP2 protein in gastric cancer tissues. Furthermore, according to the results from CCLE analysis, IGFBP2, IGFBP3, IGFBP4 and IGFBP6 are highly expressed in most cancer cell lines at the cell level. Previous studies have been conducted to explore the relationships between IGFBP family and multiple cancers. The expression of IGFBP1 has close relationship with ovarian cancer, breast cancer, liver cancer, gastric cancer, pancreatic cancer, prostate cancer and esophageal squamous cell carcinoma [31–36]. IGFBP2 is one of the most common and abundantly expressed IGFBPs in human cancers [7], which was found to be highly expressed in glioma, cervical cancer, prostate cancer, breast cancer, lung cancer, and other cancers [7, 37–40]. The expression of IGFBP3 in ovarian cancer, lung cancer, breast cancer, prostate cancer, and gastric cancer has also been reported [41–47]. IGFBP4 has been mainly studied in colon cancer, lung cancer, gastric cancer, ovarian cancer and breast cancer [47–51]. IGFBP6 expression was found to be lower in gastric cancer, nasopharyngeal cancer, colon cancer and breast cancer [52–55]. IGFBP7 is closely associated with bladder cancer, gastric cancer, breast cancer, lung cancer and acute myeloid leukemia [56–61]. However, there are relatively few studies on the expression of IGFBP5 and IGFBL1 in cancer. In addition, we found that previous studies concentrated more on the expression of IGFBP genes in gastric cancer, ovarian cancer, breast cancer, and prostate cancer, there are still some gaps to be filled for IGFBPs in other cancer types, for example, the present research is the first to reveal the expression levels of IGFBP family in renal clear cell carcinoma, head and neck squamous cell carcinoma, and endometrial carcinoma. Our study comprehensively and systematically describes the expression profile of IGFBPs at different levels in a variety of cancers, which suggesting that IGFBPs may act as cancer biomarkers and therapeutic targets.

Besides, we found that IGFBPs are related to age and gender in various cancers. Age has a great influence on the expression of IGFBPs in cancer patients. The expression of each IGFBPs is different in the age of at least five types of cancer, and the expression trend is different. For example, in CESC, the expression of IGFBP1, IGFBP2, IGFBP6, IGFBP7, and IGFBPL1 is lower in elder patients (> 60); while in LGG, IGFBP1, IGFBP2, IGFBP3, and IGFBP5 are all highly expressed in elder patients. Relatively speaking, gender has little effect on the expression of IGFBPs in cancer patients. IGFBP1 is highly expressed in male patients in GBM and HNSC, while IGFBP4 is highly expressed in female patients in BRCA, DLBC, KIRP, LIHC and SARC. This further illustrates the specificity of IGFBPs.

### IGFBPs expression was significantly associated with a variety of tumor prognosis through positive or negative two distinct styles, which makes IGFBPs available as biomarkers for prognosis

The results of this study's analysis of the connection between IGFBPs and pan-cancer prognosis showed that the expression of IGFBPs affects the prognosis of various cancers in distinct ways. IGFBP2 and IGFBP7 expression may impact the prognosis of more cancer types. Besides, most members of the IGFBP family could affect the prognosis of UVM and LGG.

It has been shown that ovarian cancer and breast cancer patients with high expression of IGFBP1 have a better prognosis [54, 62]. High mRNA expression of IGFBP2 is associated with better survival in patients with breast cancer [54] and poor survival in glioblastoma patients [63–65]. IGFBP3 expression is associated with the survival and proliferation of glioblastoma [66]. High IGF1/IGFBP3 ratio at diagnosis shows better prognosis in renal cell carcinoma [67]. Elevated IGFBP3 expression correlates with poor prognosis of pancreatic ductal adenocarcinoma, breast cancer, oral squamous cell carcinoma, papillary thyroid carcinoma and colorectal cancer [45, 54, 68–72]. Patients with high IGFBP4 mRNA expression levels have better overall survival and disease-free survival rate in breast cancer [51]. IGFBP5 is considered to be a positive indicator for the prognosis of breast cancer. For ER-positive breast cancer patients, low expression of IGFBP5 is associated with a better prognosis [51, 54, 73]. IGFBP5 is negatively related to patient survival in kidney renal papillary cell carcinoma [74]. Overexpression of IGFBP6 is associated with a better prognosis in breast cancer [54, 75]. IGFBP6 may serve as an independent prognostic factor for the prognosis of glioma patients, and overexpression of IGFBP6 can induce apoptosis of glioma cells [76, 77]. In colorectal cancer, low IGFBP6 expression is associated with poor survival [78]. Increased IGFBP7 expression is related to a better survival in breast cancer and CHOL [59, 79].

As secreted proteins, a number of studies have analyzed the correlation between circulating IGFBP concentrations and the risk or prognosis of various cancers. However, due to the complexity of the endocrine IGF–IGFBP system, many studies have produced inconclusive or even contradictory results. For example, circulating IGFBP1 and IGFBP2 appear to be involved in regulating acute bioavailability of IGFs, and when their expression is inhibited, they may increase the mitogenic activity, thereby increasing the risk of cancer [80]. However, studies of serum IGFBP1 and IGFBP2 levels associated with cancer risk or outcome have generally failed to show a consistent and definitive association [5]. Therefore, the expression of IGFBPs at mRNA or protein levels in tissues may be relatively more suitable as tumor risk or prognostic markers.

### IGFBPs expression was associated with multiple signal transduction pathways, which revealed relevant mechanism behind IGFBPs function

In order to elucidate the molecular significance of IGFBPs in the carcinogenic process, we explored the correlation between the expression of IGFBPs and signal transduction pathways. The results showed that the expression of IGFBPs is associated with a variety of cancer pathways, such as epithelial-mesenchymal transition, apoptosis, angiogenesis, TGF-β signaling, and IL2-STAT5 signaling pathway. In addition, by summarizing the number of cancer pathways related to the expression of IGFBPs, it is found that the pathways activated by IGFBPs are far more than the pathways inhibited. Therefore, IGFBPs are more likely to have carcinogenic effects. There have been previous researches indicating the associations between IGFBPs and multiple cancer-related pathways. For example, IGFBP2 can serve as a potential marker for PI3K/Akt pathway activation and PTEN status in prostate cancer as well as glioblastoma [81]. IGFBP3 promotes apoptosis and inhibits Hepg2 cell proliferation by inhibiting the signaling pathways including IGF-1R-mediated PI3K/AKT and Wnt/β-catenin, respectively [82]. In triple-negative breast cancer, IGFBP3 is involved in the PARP-dependent DNA damage repair pathway through interacting with SFPQ and NONO [83]. IGFBP4 activates the signaling pathway Wnt/β-catenin and induces M-CAM expression in human renal cell carcinoma [84]. In the present study, we found that the expression of IGFBP3, IGFBP4, IGFBP5, IGFBP6, IGFBP7 has a significant correlation with the activation of angiogenesis, epithelial-mesenchymal transition, apical junction, TGF-β signaling, KRAS signaling, and other signaling pathways. Previous studies have revealed that the above pathways are closely correlated with tumor progression [85–90].

As mentioned previously, IGFBPs regulate the activity of IGFs in a variety of ways, thereby affecting their binding to IGF receptors, and thus playing a complex regulatory role in the occurrence and development of tumors. Other studies have found that certain IGFBPs can affect tumor progression through pathways independent of IGFs, and the mechanisms are often complex and specific. For example, there are p53 response elements on the IGFBP gene which can be induced when DNA damage results in the activation of p53, and thus the IGFBP may act directly on DNA repair mechanisms [28, 91]. Currently, at least IGFBP2 and IGFBP3 have been shown to promote DNA repair [92, 93]. By regulating enzymes involved in sphingolipid metabolism, IGFBPs can affect the balance between growth-stimulating lipids (such as sphingosine-1-phosphate) and growth-inhibiting lipids (such as ceramides). This may be a key mechanism by which IGFBP3, IGFBP5, and possibly other IGFBPs may regulate the balance between cell death and survival in response to some cancer therapies [5]. Therefore, based on the role of IGFBPs in cancer and the complexity of its mechanism, our study demonstrated the relationship between IGFBP gene and cancer-related signaling pathways from the perspective of pan-cancer, providing new clues for exploring cancer-related pathways and helping to understand the specific effects on tumor cells.

The correlation and interaction between IGFBPs were also analyzed in pan-cancer. The results showed that, on the whole, the correlation between IGFBP3-6 was strong and mostly positive, while the correlation between IGFBP1, IGFBP2 or IGFBPL1 and other IGFBPs was poor. In addition, IGFBPs often showed positive correlation in different cancers. The interaction diagram established by GENEMANIA and STRING showed that there were extensive interactions between IGFBPs, especially IGFBP3 and IGFBP5, while the interaction between IGFBPL1 and other IGFBPs was relatively poor.

### IGFBPs expression was significantly correlated with multiple molecular features of tumor, which shed new light on the new strategies for tumor targeted therapy

TMB and MSI are currently clinically important tumor biomarkers for the sensitivity of immunotherapy [94–96]. Hence, we investigated the relationship between IGFBPs expression and TMB as well as MSI in 33 cancer types. The results demonstrated that the expression of IGFBP genes was significantly correlated with TMB and MSI in various cancers, and most of them were inversely correlated, suggesting that IGFBPs may indirectly affect the immunotherapeutic response in these cancers. Currently, the research literature on the relationship between IGFBPs expression and TMB or MSI is relatively less. This study reveals novel horizons for exploring the role of IGFBPs in cancer from the perspective of immunotherapy response.

As is well known, tumor stemness contributes to tumorigenesis, progression and drug resistance. The stemness index could be used to predict clinical features and outcomes for cancer patients. Stemness index was found to be increased in metastatic tumors, application of stemness scores shows potential target for cancer therapy [97, 98]. In the current study, we analyzed the correlation between IGFBPs expression and tumor stemness in 33 cancer types. DNAss and RNAss are novel stemness indices for evaluating the degree of oncogenic dedifferentiation proposed by Malta, T. M. et al. [21]. Tumor stemness score is related to multiple oncological processes. For example, higher stemness score is associated with increasing tumor dedifferentiation and biological activity of tumor stem cells. Stemness indices can be used for stratification of undifferentiated carcinoma. Stemness score is higher in metastatic cancers and correlated with intertumoral heterogeneity as well as tumor microenvironment [21, 97, 99]. The results of our study showed that the expression of most IGFBP genes was negatively correlated with the DNAss and RNAss in pan-caner, indicating that the higher expression of these IGFBPs, the less stemness features of tumor cells and the higher degree of tumor differentiation. Currently, there are few reports on the relationship between IGFBP genes and tumor stemness characteristics in human cancer. The present study provides new horizons for the IGFBP family in the development of new cancer therapies based on stem cells.

Multiple researches have revealed that tumor immune microenvironment is associated with the progression, recurrence and metastasis of tumor [100, 101], understanding the immune microenvironment of tumor patients will hopefully lead to improve the ability to predict and guide immunotherapy responses and to reveal new therapeutic targets [102, 103]. The current study analyzed the correlation between IGFBPs expression and tumor immune microenvironment. First, we used the ESTIMATE algorithm for immune and stromal score calculation. The results indicated that the expression of IGFBP genes has significantly negative or positive correlations with stromal, immune and ESTIMATE scores in various cancers. Indicating that IGFBPs expression is linked to the content of stromal and immune components in tumor tissue and purity of tumor. Previous studies have partly confirmed our findings. IGFBP2 has been reported to participate the macrophage based immunosuppressive microenvironment in pancreatic ductal adenocarcinoma [104, 105]. IGFBP3 can promote breast tumor growth by regulating tumor immune microenvironment [106]. IGFBP6 is involved in migration, immune escape and inflammation in glioblastoma [107]. Deletion of IGFBP7 presents a pro-inflammatory and immunosuppressive microenvironment and promotes the development of hepatocellular carcinoma [108].

Immune cell infiltration in tumor tissues plays important roles in tumor immune microenvironment. The present study indicated that the association between the expression of IGFBPs with immune cell infiltration was significant in 26 cancer types. Previous studies have found that IGFBP2 modulates the expression of PD-L1 through activating the EGFR-STAT3 signaling pathway, thereby exerting an anti-melanoma effect [109]. The effect of IGFBP3 in promoting the growth of breast cancer cell is related to accumulation of damaged T cells [106]. IGFBP6 protein has the function of inducing chemotaxis and enhancing the ability of neutrophils to burst and degranulate [110]. IGFBP7 can regulate the mechanism of antigen presentation, thereby generating a response of anti-tumor immune. Absence of IGFBP7 will reduce antigen presentation and reduce infiltration levels of CD4 + , CD8 + T cells, and NK cells [108]. Our research first found significant correlations between the expression of IGFBPs and various immune cell infiltration such as mast resting, macrophages M2, and naïve B cells in pan-cancer. All these findings indicate that IGFBPs can affect cancer progression by regulating immune function. Our research provides comprehensive foundation for future research on the molecular mechanisms of IGFBPs in tumor immune microenvironment.

### The mutation frequency and copy number variation of IGFBPs were generally low but had a greater impact on their expression, which revealed the potential regulation mechanism of IGFBPs expression based on their own genetic variation

The results of the mutation and CNV frequency analysis showed that the general average mutation rate of IGFBPs was 0–5.7%, of which IGFBP1 and IGFBP5 showed relatively high mutation frequency, and IGFBPL1 showed the lowest mutation frequency. The mutation frequency of IGFBPs was less than 2% in most tumors except for endometrial cancer, a type of cancer with high global mutation rates [111]. In addition, no IGFBP mutation was found in CHOL, THYM and PCPG. Studies have shown that each of IGFBP1, 3, 4, 5, and 6 is inserted or deleted in variable domains corresponding to the codon hotspot to increase mutation rates [112]. CNV frequency analysis showed that IGFBPs had a lower CNV degree in various cancers. Besides, relatively more IGFBPs mutations were found in gastric, lung, and ovarian cancer cell lines. All the results as mentioned demonstrated the variation features of IGFBPs among different cancers.

Furthermore, this study also analyzed the effects of IGFBPs genetic variation on IGFBPs expression. We found that only the mutation of IGFBP3 will affect its expression in endometrial cancer, and the mutation of IGFBP6 will affect its expression in skin melanoma. Compared with mutations, the CNV of IGFBPs has more influence on its expression in cancer, and the expression is mostly up-regulated with increased copy number. Existing studies have also proved the increase of copy number usually matches the up-regulation of expression [113]. To sum up, our findings indicate that genetic change can play crucial roles in regulating the expression of IGFBPs, and the regulation mechanism is worthy of our further research and exploration.

### IGFBP-related research has been paid more and more attention, and research hotspots mainly focus on the potential of IGFBPs as tumor diagnostic and prognostic markers, and develop related therapies accordingly

In the current study, a bibliometric and visual analysis was conducted to present the current research status and hot topics of IGFBP related studies in cancer. The results showed that the annual publication of relevant studies peaked in 2007 and then leveled off, while the number of citations maintained an increasing trend, indicating that the related studies have increasing academic value and are gaining attention. The co-occurrence network divided the keywords appearing in the top 104 into 3 clusters, and by analyzing the structure of the keywords and their intrinsic connections, we found the hot topics of related studies mainly focused on that the expression of IGFBPs was correlated with apoptosis, proliferation, differentiation, metastasis and invasion of tumor cells; IGFBPs were closely related to breast, prostate, colorectal, lung and ovarian cancers; and that IGFBPs were associated with cancer risk and prognosis and may serve as corresponding markers and therapeutic targets. The overlay visualization map of keywords showed the trends of relevant research hotspots over time. The results indicated that, with the passage of time, the keywords: invasion, poor-prognosis, migration, prognosis, biomarker, target, survival, and therapy have become increasingly popular and suggested that relative researches were more focused on the potential of IGFBPs as tumor diagnostic and prognostic markers, and develop related therapies accordingly.

In addition, through the review and quantitative analysis of the previous literature, we found that IGFBPs play an important role in the effects of cancer, including bioenergetics and metabolism, metastasis and apoptosis. Therefore, we have conducted a brief literature review on the influence of IGFBP family on the biological behavior of cancer, and compiled the relevant contents into Additional file 1: Table S7.

### Limitations

As a pan-cancer analysis of gene family, this study has not explored a single gene or a single cancer in depth and detail. However, it has comprehensively introduced the expression, prognosis, various molecular characteristics and mutations of IGFBP family in pan-cancer for the first time, which can help macroscopically understand the role of this family in pan-cancer. Furthermore, a PCA analysis may be useful to better explain the results indicate both positive and negative effects of different IGFBPs in the case of different cancer types. However, considering that the purpose of this study is to comprehensively elaborate the role of IGFBP gene family in pan-cancer, we did not conduct further PCA analysis from a specific point of view. In addition, most of the data used in this study were obtained from public databases, and there is lack of direct experimental evidence although we have carried out qRT-PCR and immunohistochemistry experiments to verify part of our results, which increases the reliability of the bioinformatics analysis.

## Conclusions and future perspectives

In conclusion, we conducted a comprehensive and systematic analysis on the expression and mutation profiles of IGFBP gene family, and its relationship with cancer-related pathways, prognosis, TMB, MSI, tumor stemness, tumor immune microenvironment in pan-cancer. At the same time, we conducted a bibliometric analysis and literature review of the latest research progress of IGFBP, and discussed the research results in detail. Our study expands the research ideas for exploring the potential of IGFBP family to be new biomarkers for cancer diagnosis or prognosis, and shed new light on relevant mechanism research.

Currently, although studies have found the multifunctional roles of IGFBPs in various cancers, there is still a long way to go before IGFBPs can be used as biomarkers for tumorigenesis or novel targets for tumor therapy, and the underlying mechanisms behind the complex functions of IGFBPs still need to be explored. Recently, numerous researches have confirmed that certain IGFBPs, particularly IGFBP2 and IGFBP3, are key tumorigenic factors and viable therapeutic targets in multiple cancers, but even so, no tumor therapies targeting IGFBP family genes are currently available clinically. IGFBP genes have been found to be involved in the oncogenic process in various cancer types, and there is evidence that IGFBPs play essential roles in tumorigenesis and suppression by regulating signal transduction pathways in tumor cells. On this basis, our study macroscopically demonstrated the relationship between IGFBP genes and cancer-related signaling pathways from a pan-cancer perspective, providing a foundation for subsequent in-depth exploration of the regulation mechanism in tumor progression affected by IGFBP genes. Recently, tumor microenvironment and immunotherapy has become research hotspot. As secreted proteins, the role of IGFBPs in immune regulation should be noted. Several studies have been conducted to develop the potential of IGFBPs as immune biomarkers and to explore its relationship with immune cells in cancer [114–118]. Our study further links the IGFBP family with tumor immune microenvironment and the levels of immune cell infiltration in 33 types of cancer, providing more clues for further studies on the mechanisms of immune regulation and the development of tumor immunotherapies. In addition, we analyzed the mutational profile of IGFBPs in pan-cancer and the effect of their mutations on their own expression. Based on previous studies, no cancers have been identified that can be attributed to IGFBP gene mutations, but alterations in IGFBP expression can be observed in many cancers. This may be explained by that the expression and biological activity of the IGFBP gene is regulated by post-translational modifications such as methylation, glycosylation and phosphorylation, as well as the differential localization of IGFBP in the pericellular and extracellular spaces [5, 30]. For example, hypermethylation of IGFBP3 has been observed in non-small cell lung cancer, prostate cancer, ovarian cancer, and hepatocellular carcinoma [119–122]. The regulation of IGFBP expression by epigenetic alterations and its mechanisms, as well as the possible changes in tumor biological behavior it may cause, still need to be investigated in depth.

Through our comprehensive analysis of the role of IGFBP gene family in pan-cancer, we found that the expression of different IGFBPs in different cancers has specificity, and its influence on prognosis, TMB, MSI and other molecular characteristics, as well as cancer-related pathways is also specific. We speculate that this specificity is mainly due to the structural specificity of different IGFBPs and the temporal-spatial specificity during development. For example, in terms of sequence structure, IGFBP1-6 and IGFBP7/IGFBPL1 are obviously in two different branches, which may be the reason why the expression patterns of IGFBP7 and IGFBPL1 are often different from other IGFBPs. In addition, in the process of ontogenesis, different IGFBPs appear in different tissues and organs at different times, which also leads to specificity. For example, STAD and COAD are both digestive system cancers, so they usually show the same expression trend. According to our study, IGFBP1 is low expressed in both two cancers, while IGFBP6 is high expressed. The same trend is also shown in KICH, KIRC and KIRP. A deep understanding of the commonality and specificity of IGFBPs genes is of great significance for a comprehensive understanding of the gene family.

## Supplementary Information

**Additional file 1: Table S1.** The 33 cancer types in TCGA pan-cancer project. **Table S2.** Primer Sequences. **Table S3.** IGFBP6 and IGFBP7 protein expression levels across 20 cancer types in the Human Protein Atlas (HPA). **Table S4.** Correlation between IGFBPs expression and signal transduction pathways. **Table S5.** Correlation between IGFBPs expression and immune cell infiltration in pan-cancer. **Table S6.** Correlation between IGFBPs CNV and IGFBPs expression. **Table S7.** The effects of IGFBPs on cancer.

**Additional file 2:** **Figure S1.** The kaplan-meier survival curve plots for the IGFBP expression on overall survival in pan-cancer. **Figure S2.** The correlation between IGFBPs expression in 24 types of cancers from TCGA. **Figure S3.** The correlation between IGFBPs expression and (A) TMB and (B) MSI in pan-cancer. **Figure S4.** The mutation details of IGFBPs in pan-cancer.

## Data Availability

The datasets supporting the conclusions of the current study are available on TCGA, (https://xenabrowser.net/datapages/); Oncomine database, (https://www.oncomine.org/); the HPA database, (https://www.proteinatlas.org/humanproteome/pathology); and the CCLE database (https://depmap.org/portal/download/all/). All the analyzed and generated data during the analysis is available from the corresponding author upon reasonable request.

## Abbreviations

ACC: Adrenocortical Carcinoma
BLCA: Bladder Urothelial Carcinoma
BRCA: Breast Invasive Carcinoma
CCLE: Cancer Cell Line Encyclopedia
CESC: Cervical Squamous Cell Carcinoma and Endocervical Adenocarcinoma
CHOL: Cholangiocarcinoma
CNV: Copy number variation
COAD: Colon Adenocarcinoma
CRC: Colorectal cancer
DLBC: Lymphoid Neoplasm Diffuse Large B-cell Lymphoma
ESCA: Esophageal Carcinoma
GBM: Glioblastoma Multiforme
GC: Gastric cancer
HNSC: Head and Neck Squamous Carcinoma
HPA: The Human Protein Atlas
IGF: Insulin-like growth factor
IGFBP: Insulin-like growth factor binding protein
KICH: Kidney Chromophobe
KIRC: Kidney Renal Clear Cell Carcinoma
KIRP: Kidney Renal Papillary Cell Carcinoma
LAML: Acute Myeloid Leukemia
LGG: Brain Lower Grade Glioma
LIHC: Liver Hepatocellular Carcinoma
LUAD: Lung Adenocarcinoma
LUSC: Lung Squamous Cell Carcinoma
MESO: Mesothelioma
MSI: Microsatellite Instability
OV: Ovarian Serous Cystadenocarcinoma
PAAD: Pancreatic Adenocarcinoma
PCPG: Pheochromocytoma and Paraganglioma
PRAD: Prostate Adenocarcinoma
READ: Rectum Adenocarcinoma
SARC: Sarcoma
SKCM: Skin Cutaneous Melanoma
STAD: Stomach Adenocarcinoma
TCGA: The Cancer Genome Atlas
TGCT: Testicular Germ Cell Tumors
THCA: Thyroid Carcinoma
THYM: Thymoma
TMB: Tumor Mutational Burden
UCEC: Uterine Corpus Endometrial Carcinoma
UCS: Uterine Carcinosarcoma
UVM: Uveal Melanoma

## References

[1] Barger CJ, Branick C, Chee L, Karpf AR (2019). Pan-Cancer Analyses Reveal Genomic Features of FOXM1 Overexpression in Cancer. Cancers (Basel).

[2] Pardini B, Cordero F, Naccarati A, Viberti C, Birolo G, Oderda M (2018). microRNA profiles in urine by next-generation sequencing can stratify bladder cancer subtypes. Oncotarget.

[3] Ma X, Liu Y, Liu Y, Alexandrov LB, Edmonson MN, Gawad C (2018). Pan-cancer genome and transcriptome analyses of 1,699 paediatric leukaemias and solid tumours. Nature.

[4] Bach LA (2018). IGF-binding proteins. J Mol Endocrinol.

[5] Baxter RC (2014). IGF binding proteins in cancer: mechanistic and clinical insights. Nat Rev Cancer.

[6] Haywood NJ, Slater TA, Matthews CJ, Wheatcroft SB (2019). The insulin like growth factor and binding protein family: novel therapeutic targets in obesity & diabetes. Mol Metab.

[7] Russo VC, Azar WJ, Yau SW, Sabin MA, Werther GA (2015). IGFBP-2: The dark horse in metabolism and cancer. Cytokine Growth Factor Rev.

[8] Price D, Muterspaugh R, Clegg B, Williams A, Stephens A, Guthrie J (2020). IGFBP-3 blocks hyaluronan-CD44 signaling, leading to increased acetylcholinesterase levels in A549 cell media and apoptosis in a p53-dependent manner. Sci Rep.

[9] Hjortebjerg R (2018). IGFBP-4 and PAPP-A in normal physiology and disease. Growth Horm IGF Res.

[10] Nguyen DV, Li Calzi S, Shaw LC, Kielczewski JL, Korah HE, Grant MB (2013). An ocular view of the IGF-IGFBP system. Growth Horm IGF Res.

[11] Bach LA, Fu P, Yang Z (2013). Insulin-like growth factor-binding protein-6 and cancer. Clin Sci (Lond).

[12] Akaogi K, Okabe Y, Funahashi K, Yoshitake Y, Nishikawa K, Yasumitsu H (1994). Cell adhesion activity of a 30-kDa major secreted protein from human bladder carcinoma cells. Biochem Biophys Res Commun.

[13] Oh Y, Nagalla SR, Yamanaka Y, Kim HS, Wilson E, Rosenfeld RG (1996). Synthesis and characterization of insulin-like growth factor-binding protein (IGFBP)-7 Recombinant human mac25 protein specifically binds IGF-I and -II. J Biol Chem.

[14] Smith P, Nicholson LJ, Syed N, Payne A, Hiller L, Garrone O (2007). Epigenetic inactivation implies independent functions for insulin-like growth factor binding protein (IGFBP)-related protein 1 and the related IGFBPL1 in inhibiting breast cancer phenotypes. Clin Cancer Res.

[15] Liu Y, Zhang M, He T, Yang W, Wang L, Zhang L (2020). Epigenetic silencing of IGFBPL1 promotes esophageal cancer growth by activating PI3K-AKT signaling. Clin Epigenetics.

[16] Love MI, Huber W, Anders S (2014). Moderated estimation of fold change and dispersion for RNA-seq data with DESeq2. Genome Biol.

[17] Hanzelmann S, Castelo R, Guinney J (2013). GSVA: gene set variation analysis for microarray and RNA-seq data. BMC Bioinformatics.

[18] Wickham H (2016). ggplot2: Elegant Graphics for Data Analysis: Springer-Verlag New York.

[19] Cohen R, Hain E, Buhard O, Guilloux A, Bardier A, Kaci R (2019). Association of primary resistance to immune checkpoint inhibitors in metastatic colorectal cancer with misdiagnosis of microsatellite instability or mismatch repair deficiency status. JAMA Oncol.

[20] Passaro A, Stenzinger A, Peters S (2020). Tumor mutational burden as a pan-cancer biomarker for immunotherapy: the limits and potential for convergence. Cancer Cell.

[21] Malta TM, Sokolov A, Gentles AJ, Burzykowski T, Poisson L, Weinstein JN (2018). Machine Learning Identifies Stemness Features Associated with Oncogenic Dedifferentiation. Cell..

[22] Yoshihara K, Shahmoradgoli M, Martinez E, Vegesna R, Kim H, Torres-Garcia W (2013). Inferring tumour purity and stromal and immune cell admixture from expression data. Nat Commun.

[23] Newman AM, Liu CL, Green MR, Gentles AJ, Feng W, Xu Y (2015). Robust enumeration of cell subsets from tissue expression profiles. Nat Methods.

[24] Gombos Z, Xu X, Chu CS, Zhang PJ, Acs G (2005). Peritumoral lymphatic vessel density and vascular endothelial growth factor C expression in early-stage squamous cell carcinoma of the uterine cervix. Clin Cancer Res.

[25] Liu D, Li L, Zhang XX, Wan DY, Xi BX, Hu Z (2014). SIX1 promotes tumor lymphangiogenesis by coordinating TGFbeta signals that increase expression of VEGF-C. Can Res.

[26] van Eck NJ, Waltman L (2010). Software survey: VOSviewer, a computer program for bibliometric mapping. Scientometrics.

[27] Jones JI, Clemmons DR (1995). Insulin-like growth factors and their binding proteins: biological actions. Endocr Rev.

[28] LeRoith D, Holly JMP, Forbes BE (2021). Insulin-like growth factors: Ligands, binding proteins, and receptors. Mol Metab.

[29] Baxter RC (1991). Insulin-like growth factor (IGF) binding proteins: the role of serum IGFBPs in regulating IGF availability. Acta Paediatr Scand Suppl.

[30] Firth SM, Baxter RC (2002). Cellular actions of the insulin-like growth factor binding proteins. Endocr Rev.

[31] Zheng Y, Sowers JY, Houston KD (2020). IGFBP-1 expression promotes tamoxifen resistance in breast cancer cells via Erk pathway activation. Front Endocrinol (Lausanne).

[32] Meng K, Cao J, Dong Y, Zhang M, Ji C, Wang X (2021). Application of bioinformatics analysis to identify important pathways and hub genes in ovarian cancer affected by WT1. Front Bioeng Biotechnol.

[33] Hwang DL, Huang SP, Lan WS, Lee PD (2003). Elevated insulin, proinsulin and insulin-like growth factor-binding protein-1 in liver disease. Growth Horm IGF Res.

[34] Xu YW, Chen H, Hong CQ, Chu LY, Yang SH, Huang LS (2020). Serum IGFBP-1 as a potential biomarker for diagnosis of early-stage upper gastrointestinal tumour. EBioMedicine.

[35] Wolpin BM, Michaud DS, Giovannucci EL, Schernhammer ES, Stampfer MJ, Manson JE (2007). Circulating insulin-like growth factor binding protein-1 and the risk of pancreatic cancer. Cancer Res.

[36] Cao Y, Nimptsch K, Shui IM, Platz EA, Wu K, Pollak MN (2015). Prediagnostic plasma IGFBP-1, IGF-1 and risk of prostate cancer. Int J Cancer.

[37] Li T, Forbes ME, Fuller GN, Li J, Yang X, Zhang W (2020). IGFBP2: integrative hub of developmental and oncogenic signaling network. Oncogene.

[38] Hu Q, Zhou Y, Ying K, Ruan W (2017). IGFBP, a novel target of lung cancer?. Clin Chim Acta.

[39] Kaur G, Balasubramaniam SD, Lee YJ (2020). IGFBP-2 in cervical cancer development. Exp Mol Pathol.

[40] Busund LT, Richardsen E, Busund R, Ukkonen T, Bjornsen T, Busch C (2005). Significant expression of IGFBP2 in breast cancer compared with benign lesions. J Clin Pathol.

[41] Bai R, Cui Z, Ma Y, Wu Y, Wang N, Huang L (2019). The NF-kappaB-modulated miR-19a-3p enhances malignancy of human ovarian cancer cells through inhibition of IGFBP-3 expression. Mol Carcinog.

[42] Shih HJ, Chang HF, Chen CL, Torng PL (2021). Differential expression of hypoxia-inducible factors related to the invasiveness of epithelial ovarian cancer. Sci Rep.

[43] Chen X, Shao Y, Wei W, Shen H, Li Y, Chen Y (2021). Downregulation of LOX promotes castration-resistant prostate cancer progression via IGFBP3. J Cancer.

[44] Igarashi K, Yui Y, Watanabe K, Kumai J, Nishizawa Y, Miyaura C (2020). Molecular evidence of IGFBP-3 dependent and independent VD3 action and its nonlinear response on IGFBP-3 induction in prostate cancer cells. BMC Cancer.

[45] Zielinska HA, Daly CS, Alghamdi A, Bahl A, Sohail M, White P (2020). Interaction between GRP78 and IGFBP-3 affects tumourigenesis and prognosis in breast cancer patients. Cancers (Basel)..

[46] Day TF, Kallakury BVS, Ross JS, Voronel O, Vaidya S, Sheehan CE (2019). Dual targeting of EGFR and IGF1R in the TNFAIP8 knockdown non-small cell lung cancer cells. Mol Cancer Res.

[47] Liu Q, Jiang J, Zhang X, Zhang M, Fu Y (2021). Comprehensive analysis of IGFBPs as biomarkers in gastric cancer. Front Oncol.

[48] Li W, Sun D, Lv Z, Wei Y, Zheng L, Zeng T (2017). Insulin-like growth factor binding protein-4 inhibits cell growth, migration and invasion, and downregulates COX-2 expression in A549 lung cancer cells. Cell Biol Int.

[49] Wegmann BR, Schoneberger HJ, Kiefer PE, Jaques G, Brandscheid D, Havemann K (1993). Molecular cloning of IGFBP-5 from SCLC cell lines and expression of IGFBP-4, IGFBP-5 and IGFBP-6 in lung cancer cell lines and primary tumours. Eur J Cancer.

[50] Durai R, Davies M, Yang W, Yang SY, Seifalian A, Goldspink G (2006). Biology of insulin-like growth factor binding protein-4 and its role in cancer (review). Int J Oncol.

[51] Mita K, Zhang Z, Ando Y, Toyama T, Hamaguchi M, Kobayashi S (2007). Prognostic significance of insulin-like growth factor binding protein (IGFBP)-4 and IGFBP-5 expression in breast cancer. Jpn J Clin Oncol.

[52] Zeng C, Feng X, Wang W, Lv L, Fang C, Chi L (2017). Decreased expression of insulin-like growth factor binding protein 6 is associated with gastric adenocarcinoma prognosis. Oncol Lett.

[53] Chen Q, Qin S, Liu Y, Hong M, Qian CN, Keller ET (2016). IGFBP6 is a novel nasopharyngeal carcinoma prognostic biomarker. Oncotarget.

[54] Wang J, Luo XX, Tang YL, Xu JX, Zeng ZG (2019). The prognostic values of insulin-like growth factor binding protein in breast cancer. Medicine (Baltimore).

[55] Oh YS, Kim EJ, Schaffer BS, Kang YH, Binderup L, MacDonald RG (2001). Synthetic low-calcaemic vitamin D(3) analogues inhibit secretion of insulin-like growth factor II and stimulate production of insulin-like growth factor-binding protein-6 in conjunction with growth suppression of HT-29 colon cancer cells. Mol Cell Endocrinol.

[56] Yi X, Zheng X, Xu H, Li J, Zhang T, Ge P (2022). IGFBP7 and the tumor immune landscape: a novel target for immunotherapy in bladder cancer. Front Immunol.

[57] Artico LL, Laranjeira ABA, Campos LW, Correa JR, Zenatti PP, Carvalheira JBC (2021). Physiologic IGFBP7 levels prolong IGF1R activation in acute lymphoblastic leukemia. Blood Adv.

[58] van Gils N, Verhagen H, Rutten A, Menezes RX, Tsui ML, Vermue E (2020). IGFBP7 activates retinoid acid-induced responses in acute myeloid leukemia stem and progenitor cells. Blood Adv.

[59] Godina C, Khazaei S, Tryggvadottir H, Visse E, Nodin B, Jirstrom K (2021). Prognostic impact of tumor-specific insulin-like growth factor binding protein 7 (IGFBP7) levels in breast cancer: a prospective cohort study. Carcinogenesis.

[60] Zhao Q, Zhao R, Song C, Wang H, Rong J, Wang F (2021). Increased IGFBP7 expression correlates with poor prognosis and immune infiltration in gastric cancer. J Cancer.

[61] Tang X, Mu J, Ma L, Tan Q, Wang J, Tan J (2021). IGFBP7 overexpression promotes acquired resistance to AZD9291 in non-small cell lung cancer. Biochem Biophys Res Commun.

[62] Zheng R, Chen W, Xia W, Zheng J, Zhou Q (2020). The prognostic values of the insulin-like growth factor binding protein family in ovarian cancer. Biomed Res Int.

[63] Zhang GH, Zhong QY, Gou XX, Fan EX, Shuai Y, Wu MN (2019). Seven genes for the prognostic prediction in patients with glioma. Clin Transl Oncol.

[64] Yuan Q, Cai HQ, Zhong Y, Zhang MJ, Cheng ZJ, Hao JJ (2019). Overexpression of IGFBP2 mRNA predicts poor survival in patients with glioblastoma. Biosci Rep.

[65] Lindstrom MS (2019). Expanding the scope of candidate prognostic marker IGFBP2 in glioblastoma. Biosci Rep.

[66] Yang J, Hu Y, Wu J, Kong S (2020). Effects of IGFBP-3 and GalNAc-T14 on proliferation and cell cycle of glioblastoma cells and its mechanism. J Pharm Pharmacol.

[67] Tsai CW, Chang WS, Xu Y, Huang M, Tamboli P, Wood CG (2022). Prognostic significance of circulating insulin growth-like factor 1 and insulin growth-like factor binding protein 3 in renal cell carcinoma patients. Am J Cancer Res.

[68] Gheysarzadeh A, Bakhtiari H, Ansari A, Emami Razavi A, Emami MH, Mofid MR (2019). The insulin-like growth factor binding protein-3 and its death receptor in pancreatic ductal adenocarcinoma poor prognosis. J Cell Physiol.

[69] Huang Y, Chang A, Zhou W, Zhao H, Zhuo X (2020). IGFBP3 as an indicator of lymph node metastasis and unfavorable prognosis for papillary thyroid carcinoma. Clin Exp Med.

[70] Sakata J, Hirosue A, Yoshida R, Matsuoka Y, Kawahara K, Arita H (2020). Enhanced expression of IGFBP-3 reduces radiosensitivity and is associated with poor prognosis in oral squamous cell carcinoma. Cancers (Basel).

[71] Yamamoto N, Oshima T, Yoshihara K, Aoyama T, Hayashi T, Yamada T (2017). Clinicopathological significance and impact on outcomes of the gene expression levels of IGF-1, IGF-2 and IGF-1R, IGFBP-3 in patients with colorectal cancer: overexpression of the IGFBP-3 gene is an effective predictor of outcomes in patients with colorectal cancer. Oncol Lett.

[72] Hou YL, Luo P, Ji GY, Chen H (2019). Clinical significance of serum IGFBP-3 in colorectal cancer. J Clin Lab Anal.

[73] Li X, Cao X, Li X, Zhang W, Feng Y (2007). Expression level of insulin-like growth factor binding protein 5 mRNA is a prognostic factor for breast cancer. Cancer Sci.

[74] Wang S, Hong Q, Geng X, Chi K, Cai G, Wu D (2019). Insulin-Like growth factor binding protein 5-A probable target of kidney renal papillary renal cell carcinoma. Biomed Res Int.

[75] Shkurnikov MY, Poloznikov AA, Nikulin SV, Schumacher U, Wicklein D, Sturken C (2019). Transcriptome guided drug combination suppresses proliferation of breast cancer cells. Bull Exp Biol Med.

[76] Bei Y, Huang Q, Shen J, Shi J, Shen C, Xu P (2017). IGFBP6 regulates cell apoptosis and migration in glioma. Cell Mol Neurobiol.

[77] Zong Z, Xin L, Tang X, Guo H (2022). The clinical characteristics and prognostic value of IGFBP6 in glioma. Neurol Res.

[78] Zhao C, Zhu X, Wang G, Wang W, Ju S, Wang X (2020). Decreased expression of IGFBP6 correlates with poor survival in colorectal cancer patients. Pathol Res Pract.

[79] Yue C, Yang M, Tian Q, Mo F, Peng J, Ma Y (2018). IGFBP7 is associated to prognosis and could suppress cell survival in cholangiocarcinoma. Artif Cells Nanomed Biotechnol.

[80] Renehan AG, Frystyk J, Flyvbjerg A (2006). Obesity and cancer risk: the role of the insulin-IGF axis. Trends Endocrinol Metab.

[81] Mehrian-Shai R, Chen CD, Shi T, Horvath S, Nelson SF, Reichardt JK (2007). Insulin growth factor-binding protein 2 is a candidate biomarker for PTEN status and PI3K/Akt pathway activation in glioblastoma and prostate cancer. Proc Natl Acad Sci U S A.

[82] Liu QW, Li JY, Zhang XC, Liu Y, Liu QY, Xiao L (2020). Human amniotic mesenchymal stem cells inhibit hepatocellular carcinoma in tumour-bearing mice. J Cell Mol Med.

[83] de Silva HC, Lin MZ, Phillips L, Martin JL, Baxter RC (2019). IGFBP-3 interacts with NONO and SFPQ in PARP-dependent DNA damage repair in triple-negative breast cancer. Cell Mol Life Sci.

[84] Ueno K, Hirata H, Majid S, Tabatabai ZL, Hinoda Y, Dahiya R (2011). IGFBP-4 activates the Wnt/beta-catenin signaling pathway and induces M-CAM expression in human renal cell carcinoma. Int J Cancer.

[85] Sajib S, Zahra FT, Lionakis MS, German NA, Mikelis CM (2018). Mechanisms of angiogenesis in microbe-regulated inflammatory and neoplastic conditions. Angiogenesis.

[86] Dongre A, Weinberg RA (2019). New insights into the mechanisms of epithelial-mesenchymal transition and implications for cancer. Nat Rev Mol Cell Biol.

[87] Pastushenko I, Blanpain C (2019). EMT transition states during tumor progression and metastasis. Trends Cell Biol.

[88] Gonzalez-Mariscal L, Miranda J, Gallego-Gutierrez H, Cano-Cortina M, Amaya E (2020). Relationship between apical junction proteins, gene expression and cancer. Biochim Biophys Acta Biomembr.

[89] Seoane J, Gomis RR (2017). TGF-beta Family signaling in tumor suppression and cancer progression. Cold Spring Harb Perspect Biol.

[90] Canon J, Rex K, Saiki AY, Mohr C, Cooke K, Bagal D (2019). The clinical KRAS(G12C) inhibitor AMG 510 drives anti-tumour immunity. Nature.

[91] Chua MW, Lin MZ, Martin JL, Baxter RC (2015). Involvement of the insulin-like growth factor binding proteins in the cancer cell response to DNA damage. J Cell Commun Signal.

[92] Hollowood AD, Lai T, Perks CM, Newcomb PV, Alderson D, Holly JM (2000). IGFBP-3 prolongs the p53 response and enhances apoptosis following UV irradiation. Int J Cancer.

[93] Zhou Z, Lu H, Zhu S, Gomaa A, Chen Z, Yan J (2019). Activation of EGFR-DNA-PKcs pathway by IGFBP2 protects esophageal adenocarcinoma cells from acidic bile salts-induced DNA damage. J Exp Clin Cancer Res.

[94] Salem ME, Bodor JN, Puccini A, Xiu J, Goldberg RM, Grothey A (2020). Relationship between MLH1, PMS2, MSH2 and MSH6 gene-specific alterations and tumor mutational burden in 1057 microsatellite instability-high solid tumors. Int J Cancer.

[95] Luchini C, Bibeau F, Ligtenberg MJL, Singh N, Nottegar A, Bosse T (2019). ESMO recommendations on microsatellite instability testing for immunotherapy in cancer, and its relationship with PD-1/PD-L1 expression and tumour mutational burden: a systematic review-based approach. Ann Oncol.

[96] Innocenti F, Ou FS, Qu X, Zemla TJ, Niedzwiecki D, Tam R (2019). Mutational Analysis of Patients With Colorectal Cancer in CALGB/SWOG 80405 Identifies New Roles of Microsatellite Instability and Tumor Mutational Burden for Patient Outcome. J Clin Oncol.

[97] Ben-Porath I, Thomson MW, Carey VJ, Ge R, Bell GW, Regev A (2008). An embryonic stem cell-like gene expression signature in poorly differentiated aggressive human tumors. Nat Genet.

[98] Kooreman NG, Kim Y, de Almeida PE, Termglinchan V, Diecke S, Shao NY (2018). Autologous iPSC-based vaccines elicit anti-tumor responses in vivo. Cell Stem Cell.

[99] Chen P, Hsu WH, Han J, Xia Y, DePinho RA (2021). Cancer Stemness meets immunity: from mechanism to therapy. Cell Rep.

[100] Pitt JM, Marabelle A, Eggermont A, Soria JC, Kroemer G, Zitvogel L (2016). Targeting the tumor microenvironment: removing obstruction to anticancer immune responses and immunotherapy. Ann Oncol.

[101] Croci DO, Zacarias Fluck MF, Rico MJ, Matar P, Rabinovich GA, Scharovsky OG (2007). Dynamic cross-talk between tumor and immune cells in orchestrating the immunosuppressive network at the tumor microenvironment. Cancer Immunol Immunother.

[102] Binnewies M, Roberts EW, Kersten K, Chan V, Fearon DF, Merad M (2018). Understanding the tumor immune microenvironment (TIME) for effective therapy. Nat Med.

[103] Fu T, Dai LJ, Wu SY, Xiao Y, Ma D, Jiang YZ (2021). Spatial architecture of the immune microenvironment orchestrates tumor immunity and therapeutic response. J Hematol Oncol.

[104] Sun L, Zhang X, Song Q, Liu L, Forbes E, Tian W (2021). IGFBP2 promotes tumor progression by inducing alternative polarization of macrophages in pancreatic ductal adenocarcinoma through the STAT3 pathway. Cancer Lett.

[105] Gao S, Sun Y, Zhang X, Hu L, Liu Y, Chua CY (2016). IGFBP2 activates the NF-kappaB pathway to drive epithelial-mesenchymal transition and invasive character in pancreatic ductal adenocarcinoma. Cancer Res.

[106] Scully T, Scott CD, Firth SM, Sedger LM, Pintar JE, Twigg SM (2018). Enhancement of mammary tumour growth by IGFBP-3 involves impaired T cell accumulation. Endocr Relat Cancer.

[107] Longhitano L, Vicario N, Forte S, Giallongo C, Broggi G, Caltabiano R, et al. Lactate modulates microglia polarization via IGFBP6 expression and remodels tumor microenvironment in glioblastoma. Cancer Immunol Immunother. 2023;72(1):1–20. 10.1007/s00262-022-03215-3 PMC9813126 35654889

[108] Akiel M, Guo C, Li X, Rajasekaran D, Mendoza RG, Robertson CL (2017). IGFBP7 deletion promotes hepatocellular carcinoma. Cancer Res.

[109] Li T, Zhang C, Zhao G, Zhang X, Hao M, Hassan S (2020). IGFBP2 regulates PD-L1 expression by activating the EGFR-STAT3 signaling pathway in malignant melanoma. Cancer Lett.

[110] Liso A, Capitanio N, Gerli R, Conese M (2018). From fever to immunity: a new role for IGFBP-6?. J Cell Mol Med.

[111] Rutgers J (2015). Update on pathology, staging and molecular pathol-ogy of endometrial (uterine corpus) adenocarcinoma. Future Oncol.

[112] Gordon PV, Marcinkiewicz M (2008). An analysis of IGFBP evolution. Growth Horm IGF Res.

[113] Lu TP, Lai LC, Tsai MH, Chen PC, Hsu CP, Lee JM (2011). Integrated analyses of copy number variations and gene expression in lung adenocarcinoma. PLoS One.

[114] Klemm F, Maas RR, Bowman RL, Kornete M, Soukup K, Nassiri S (2020). Interrogation of the Microenvironmental landscape in brain tumors reveals disease-specific alterations of immune cells. Cell.

[115] Quail DF, Joyce JA (2017). The Microenvironmental landscape of brain tumors. Cancer Cell.

[116] Yasuoka H, Yamaguchi Y, Feghali-Bostwick CA (2009). The pro-fibrotic factor IGFBP-5 induces lung fibroblast and mononuclear cell migration. Am J Respir Cell Mol Biol.

[117] Chesik D, De Keyser J, Wilczak N (2004). Involvement of insulin-like growth factor binding protein-2 in activated microglia as assessed in post mortem human brain. Neurosci Lett.

[118] Mantovani A, Allavena P, Sica A, Balkwill F (2008). Cancer-related inflammation. Nature.

[119] Hanafusa T, Yumoto Y, Nouso K, Nakatsukasa H, Onishi T, Fujikawa T (2002). Reduced expression of insulin-like growth factor binding protein-3 and its promoter hypermethylation in human hepatocellular carcinoma. Cancer Lett.

[120] Ibanez de Caceres I, Cortes-Sempere M, Moratilla C, Machado-Pinilla R, Rodriguez-Fanjul V, Manguan-Garcia C (2010). IGFBP-3 hypermethylation-derived deficiency mediates cisplatin resistance in non-small-cell lung cancer. Oncogene..

[121] Perry AS, Loftus B, Moroose R, Lynch TH, Hollywood D, Watson RW (2007). In silico mining identifies IGFBP3 as a novel target of methylation in prostate cancer. Br J Cancer.

[122] Wiley A, Katsaros D, Fracchioli S, Yu H (2006). Methylation of the insulin-like growth factor binding protein-3 gene and prognosis of epithelial ovarian cancer. Int J Gynecol Cancer.

