# Supplementary material for: Expression characteristics and their functional role of IGFBP gene family in pan-cancer
Source: BMC Cancer. 2023 Apr 24;23:371. doi: 10.1186/s12885-023-10832-3 (PMC10124011; doi:10.1186/s12885-023-10832-3)
Supplement: Supplementary file 1 — Additional file 1: Table S1. The 33 cancer types in TCGA pan-cancer project. Table S2. Primer Sequences. Table S3. IGFBP6 and IGFBP7 protein expression levels across 20 cancer types in the Human Protein Atlas (HPA). Table S4. Correlation between IGFBPs expression and signal transduction pathways. Table S5. Correlation between IGFBPs expression and immune cell infiltration in pan-cancer. Table S6. Correlation between IGFBPs CNV and IGFBPs expression. Table S7. The effects of IGFBPs on cancer. [file 12885_2023_10832_MOESM1_ESM.docx]

| **Table S1 The 33 cancer types in TCGA pan-cancer project** | | |
| --- | --- | --- |
| **Number** | **Cancers** | **Abbreviations** |
| 1 | Adrenocortical Carcinoma | ACC |
| 2 | Bladder Urothelial Carcinoma | BLCA |
| 3 | Breast Invasive Carcinoma | BRCA |
| 4 | Cervical Squamous Cell Carcinoma and Endocervical Adenocarcinoma | CESC |
| 5 | Cholangiocarcinoma | CHOL |
| 6 | Colon Adenocarcinoma | COAD |
| 7 | Lymphoid Neoplasm Diffuse Large B-cell Lymphoma | DLBC |
| 8 | Esophageal Carcinoma | ESCA |
| 9 | Glioblastoma Multiforme | GBM |
| 10 | Head and Neck Squamous Carcinoma | HNSC |
| 11 | Kidney Chromophobe | KICH |
| 12 | Kidney Renal Clear Cell Carcinoma | KIRC |
| 13 | Kidney Renal Papillary Cell Carcinoma | KIRP |
| 14 | Acute Myeloid Leukemia | LAML |
| 15 | Brain Lower Grade Glioma | LGG |
| 16 | Liver Hepatocellular Carcinoma | LIHC |
| 17 | Lung Adenocarcinoma | LUAD |
| 18 | Lung Squamous Cell Carcinoma | LUSC |
| 19 | Mesothelioma | MESO |
| 20 | Ovarian Serous Cystadenocarcinoma | OV |
| 21 | Pancreatic Adenocarcinoma | PAAD |
| 22 | Pheochromocytoma and Paraganglioma | PCPG |
| 23 | Prostate Adenocarcinoma | PRAD |
| 24 | Rectum Adenocarcinoma | READ |
| 25 | Sarcoma | SARC |
| 26 | Skin Cutaneous Melanoma | SKCM |
| 27 | Stomach Adenocarcinoma | STAD |
| 28 | Testicular Germ Cell Tumors | TGCT |
| 29 | Thyroid Carcinoma | THCA |
| 30 | Thymoma | THYM |
| 31 | Uterine Corpus Endometrial Carcinoma | UCEC |
| 32 | Uterine Carcinosarcoma | UCS |
| 33 | Uveal Melanoma | UVM |

**Table S2 Primer Sequences**

| **Primer name** | **Sequence** |
| --- | --- |
| IGFBP1-F | GTTTAGCCAAGGCACAGGAGA |
| IGFBP1-R | CATGGATGTCTCACACTGTCTGC |
| IGFBP2-F | CGAGGGCACTTGTGAGAAGC |
| IGFBP2-R | CCTTCTGAGTGGTCATCGCC |
| IGFBP6-F | ATTCTGCGGGTGTCCAAGAC |
| IGFBP6-R | TGTTTGAGCCCCTCGGTAGA |
| beta-actin-F | ATGTGGCCGAGGACTTTGATT |
| beta-actin-R | AGTGGGGTGGCTTTTAGGATG |

| **Table S3 IGFBP6 and IGFBP7 protein expression levels across 20 cancer types in the Human Protein Atlas (HPA)** | | | | | | |
| --- | --- | --- | --- | --- | --- | --- |
| **Gene** | **Cancer Type** | **Protein expression** | | **Gene** | **Protein expression** | |
|  |  | **High or Medium** | **Low or Not detected** |  | **High or Medium** | **Low or Not detected** |
| IGFBP6 | glioma | 84.62% | 15.38% | IGFBP7 | 63.64% | 36.36% |
| IGFBP6 | THCA | 50.00% | 50.00% | IGFBP7 | 25.00% | 75.00% |
| IGFBP6 | LUCA | 75.00% | 25.00% | IGFBP7 | 50.00% | 50.00% |
| IGFBP6 | COCA | 63.64% | 36.36% | IGFBP7 | 0.00% | 100.00% |
| IGFBP6 | HNSC | 33.33% | 66.67% | IGFBP7 | 25.00% | 75.00% |
| IGFBP6 | STCA | 25.00% | 75.00% | IGFBP7 | 0.00% | 100.00% |
| IGFBP6 | LIHC | 16.67% | 83.33% | IGFBP7 | 22.22% | 77.78% |
| IGFBP6 | carcinoid | 0.00% | 100.00% | IGFBP7 | 0.00% | 100.00% |
| IGFBP6 | PACA | 41.67% | 58.33% | IGFBP7 | 42.86% | 57.14% |
| IGFBP6 | RACA | 75.00% | 25.00% | IGFBP7 | 50.00% | 50.00% |
| IGFBP6 | URCA | 54.55% | 45.45% | IGFBP7 | 33.33% | 66.67% |
| IGFBP6 | PRCA | 16.67% | 83.33% | IGFBP7 | 10.00% | 90.00% |
| IGFBP6 | TECA | 83.33% | 16.67% | IGFBP7 | 37.50% | 62.50% |
| IGFBP6 | BRCA | 8.33% | 91.67% | IGFBP7 | 10.00% | 90.00% |
| IGFBP6 | CECA | 8.33% | 91.67% | IGFBP7 | 50.00% | 50.00% |
| IGFBP6 | ENCA | 81.82% | 18.18% | IGFBP7 | 72.73% | 27.27% |
| IGFBP6 | OV | 63.64% | 36.36% | IGFBP7 | 50.00% | 50.00% |
| IGFBP6 | melanoma | 66.67% | 33.33% | IGFBP7 | 50.00% | 50.00% |
| IGFBP6 | SKCA | 0.00% | 100.00% | IGFBP7 | 50.00% | 50.00% |
| IGFBP6 | lymphoma | 66.67% | 33.33% | IGFBP7 | 0.00% | 100.00% |

| **Table S4 Correlation between IGFBPs expression and signal transduction pathways** | | | | | | | | | | | | | | | | |
| --- | --- | --- | --- | --- | --- | --- | --- | --- | --- | --- | --- | --- | --- | --- | --- | --- |
| **Pathway** | **IGFBP1** | | **IGFBP2** | | **IGFBP3** | | **IGFBP4** | | **IGFBP5** | | **IGFBP6** | | **IGFBP7** | | **IGFBPL1** | |
|  | **r** | ***P*** | **r** | ***P*** | **r** | ***P*** | **r** | ***P*** | **r** | ***P*** | **r** | ***P*** | **r** | ***P*** | **r** | ***P*** |
| ADIPOGENESIS | 0.334 | <0.001 | -0.02 | 0.047 | 0.152 | <0.001 | 0.288 | <0.001 | 0.008 | 0.435 | -0.004 | 0.659 | 0.25 | <0.001 | -0.188 | <0.001 |
| ALLOGRAFT_REJECTION | -0.01 | 0.318 | -0.07 | <0.001 | 0.217 | <0.001 | 0.24 | <0.001 | 0.028 | 0.004 | 0.171 | <0.001 | 0.169 | <0.001 | -0.158 | <0.001 |
| ANDROGEN_RESPONSE | -0.039 | <0.001 | 0.169 | <0.001 | 0.21 | <0.001 | 0.277 | <0.001 | 0.315 | <0.001 | 0.104 | <0.001 | 0.155 | <0.001 | 0.031 | 0.002 |
| ANGIOGENESIS | 0.189 | <0.001 | 0.062 | <0.001 | 0.533 | <0.001 | 0.552 | <0.001 | 0.456 | <0.001 | 0.364 | <0.001 | 0.542 | <0.001 | 0 | 0.993 |
| APICAL_JUNCTION | 0 | 0.99 | 0.089 | <0.001 | 0.394 | <0.001 | 0.468 | <0.001 | 0.449 | <0.001 | 0.488 | <0.001 | 0.428 | <0.001 | 0.079 | <0.001 |
| APICAL_SURFACE | -0.082 | <0.001 | 0.096 | <0.001 | 0.373 | <0.001 | 0.403 | <0.001 | 0.258 | <0.001 | 0.312 | <0.001 | 0.239 | <0.001 | -0.042 | <0.001 |
| APOPTOSIS | 0.02 | 0.039 | 0.048 | <0.001 | 0.399 | <0.001 | 0.481 | <0.001 | 0.277 | <0.001 | 0.359 | <0.001 | 0.305 | <0.001 | -0.084 | <0.001 |
| BILE_ACID_METABOLISM | 0.445 | <0.001 | -0.082 | <0.001 | 0.044 | <0.001 | 0.259 | <0.001 | 0.07 | <0.001 | -0.042 | <0.001 | 0.236 | <0.001 | -0.135 | <0.001 |
| CHOLESTEROL_HOMEOSTASIS | 0.203 | <0.001 | 0.247 | <0.001 | 0.175 | <0.001 | 0.144 | <0.001 | -0.064 | <0.001 | -0.073 | <0.001 | -0.113 | <0.001 | -0.047 | <0.001 |
| COAGULATION | 0.376 | <0.001 | 0.047 | <0.001 | 0.41 | <0.001 | 0.58 | <0.001 | 0.279 | <0.001 | 0.337 | <0.001 | 0.446 | <0.001 | -0.091 | <0.001 |
| COMPLEMENT | 0.197 | <0.001 | -0.079 | <0.001 | 0.346 | <0.001 | 0.386 | <0.001 | 0.177 | <0.001 | 0.234 | <0.001 | 0.33 | <0.001 | -0.108 | <0.001 |
| DNA_REPAIR | -0.104 | <0.001 | 0.189 | <0.001 | -0.099 | <0.001 | -0.124 | <0.001 | -0.366 | <0.001 | -0.085 | <0.001 | -0.367 | <0.001 | -0.101 | <0.001 |
| E2F_TARGETS | -0.18 | <0.001 | 0.192 | <0.001 | -0.012 | 0.236 | -0.18 | <0.001 | -0.23 | <0.001 | -0.213 | <0.001 | -0.408 | <0.001 | 0.028 | 0.004 |
| EPITHELIAL_MESENCHYMAL_TRANSITION | 0.03 | 0.002 | 0.143 | <0.001 | 0.485 | <0.001 | 0.552 | <0.001 | 0.515 | <0.001 | 0.424 | <0.001 | 0.484 | <0.001 | 0.046 | <0.001 |
| ESTROGEN_RESPONSE_EARLY | -0.079 | <0.001 | 0.203 | <0.001 | 0.253 | <0.001 | 0.474 | <0.001 | 0.346 | <0.001 | 0.196 | <0.001 | 0.161 | <0.001 | -0.01 | 0.3 |
| ESTROGEN_RESPONSE_LATE | -0.063 | <0.001 | 0.288 | <0.001 | 0.238 | <0.001 | 0.434 | <0.001 | 0.198 | <0.001 | 0.176 | <0.001 | 0.037 | <0.001 | -0.015 | 0.121 |
| FATTY_ACID_METABOLISM | 0.31 | <0.001 | 0.03 | 0.003 | 0.093 | <0.001 | 0.188 | <0.001 | -0.142 | <0.001 | -0.087 | <0.001 | 0.075 | <0.001 | -0.235 | <0.001 |
| G2M_CHECKPOINT | -0.229 | <0.001 | 0.193 | <0.001 | -0.001 | 0.951 | -0.163 | <0.001 | -0.154 | <0.001 | -0.19 | <0.001 | -0.391 | <0.001 | 0.054 | <0.001 |
| GLYCOLYSIS | 0.126 | <0.001 | 0.239 | <0.001 | 0.435 | <0.001 | 0.286 | <0.001 | -0.024 | 0.013 | 0.042 | <0.001 | -0.023 | 0.02 | -0.105 | <0.001 |
| HEDGEHOG_SIGNALING | 0.094 | <0.001 | 0.004 | 0.711 | 0.235 | <0.001 | 0.11 | <0.001 | 0.483 | <0.001 | 0.204 | <0.001 | 0.414 | <0.001 | 0.269 | <0.001 |
| HEME_METABOLISM | 0.24 | <0.001 | -0.208 | <0.001 | 0.083 | <0.001 | 0.107 | <0.001 | 0.15 | <0.001 | 0.08 | <0.001 | 0.313 | <0.001 | 0.001 | 0.934 |
| HYPOXIA | 0.207 | <0.001 | 0.059 | <0.001 | 0.594 | <0.001 | 0.421 | <0.001 | 0.283 | <0.001 | 0.301 | <0.001 | 0.359 | <0.001 | -0.084 | <0.001 |
| IL2_STAT5_SIGNALING | 0.073 | <0.001 | -0.044 | <0.001 | 0.387 | <0.001 | 0.433 | <0.001 | 0.201 | <0.001 | 0.311 | <0.001 | 0.312 | <0.001 | -0.128 | <0.001 |
| IL6_JAK_STAT3_SIGNALING | 0.121 | <0.001 | -0.046 | <0.001 | 0.332 | <0.001 | 0.335 | <0.001 | 0.092 | <0.001 | 0.231 | <0.001 | 0.248 | <0.001 | -0.159 | <0.001 |
| INFLAMMATORY_RESPONSE | 0.062 | <0.001 | -0.043 | <0.001 | 0.348 | <0.001 | 0.328 | <0.001 | 0.154 | <0.001 | 0.249 | <0.001 | 0.254 | <0.001 | -0.118 | <0.001 |
| INTERFERON_ALPHA_RESPONSE | 0.033 | 0.001 | -0.005 | 0.645 | 0.217 | <0.001 | 0.21 | <0.001 | -0.025 | 0.01 | 0.122 | <0.001 | 0.089 | <0.001 | -0.168 | <0.001 |
| INTERFERON_GAMMA_RESPONSE | 0.047 | <0.001 | -0.043 | <0.001 | 0.286 | <0.001 | 0.267 | <0.001 | 0.024 | 0.014 | 0.168 | <0.001 | 0.166 | <0.001 | -0.167 | <0.001 |
| KRAS_SIGNALING_DN | -0.023 | 0.019 | 0.318 | <0.001 | 0.119 | <0.001 | 0.074 | <0.001 | 0.177 | <0.001 | 0.228 | <0.001 | 0.021 | 0.033 | 0.228 | <0.001 |
| KRAS_SIGNALING_UP | 0.192 | <0.001 | -0.04 | <0.001 | 0.369 | <0.001 | 0.407 | <0.001 | 0.329 | <0.001 | 0.303 | <0.001 | 0.429 | <0.001 | -0.016 | 0.095 |
| MITOTIC_SPINDLE | -0.133 | <0.001 | 0.051 | <0.001 | 0.104 | <0.001 | -0.041 | <0.001 | 0.148 | <0.001 | -0.024 | 0.014 | -0.108 | <0.001 | 0.193 | <0.001 |
| MTORC1_SIGNALING | 0.022 | 0.023 | 0.198 | <0.001 | 0.238 | <0.001 | 0.04 | <0.001 | -0.133 | <0.001 | -0.116 | <0.001 | -0.216 | <0.001 | -0.052 | <0.001 |
| MYC_TARGETS_V1 | -0.204 | <0.001 | 0.26 | <0.001 | -0.014 | 0.154 | -0.148 | <0.001 | -0.223 | <0.001 | -0.144 | <0.001 | -0.374 | <0.001 | -0.002 | 0.862 |
| MYC_TARGETS_V2 | -0.126 | <0.001 | 0.221 | <0.001 | -0.068 | <0.001 | -0.163 | <0.001 | -0.366 | <0.001 | -0.159 | <0.001 | -0.45 | <0.001 | -0.134 | <0.001 |
| MYOGENESIS | 0.094 | <0.001 | 0.038 | <0.001 | 0.287 | <0.001 | 0.387 | <0.001 | 0.454 | <0.001 | 0.467 | <0.001 | 0.511 | <0.001 | 0.091 | <0.001 |
| NOTCH_SIGNALING | 0.06 | <0.001 | 0.14 | <0.001 | 0.311 | <0.001 | 0.257 | <0.001 | 0.384 | <0.001 | 0.247 | <0.001 | 0.299 | <0.001 | 0.087 | <0.001 |
| OXIDATIVE_PHOSPHORYLATION | 0.168 | <0.001 | 0.034 | <0.001 | -0.046 | <0.001 | -0.016 | 0.098 | -0.285 | <0.001 | -0.107 | <0.001 | -0.042 | <0.001 | -0.169 | <0.001 |
| P53_PATHWAY | 0.002 | 0.818 | 0.046 | <0.001 | 0.361 | <0.001 | 0.381 | <0.001 | 0.043 | <0.001 | 0.395 | <0.001 | 0.141 | <0.001 | -0.19 | <0.001 |
| PANCREAS_BETA_CELLS | 0.333 | <0.001 | 0.028 | 0.004 | 0.038 | <0.001 | 0.019 | 0.059 | 0.216 | <0.001 | -0.01 | 0.292 | 0.293 | <0.001 | 0.182 | <0.001 |
| PEROXISOME | 0.238 | <0.001 | 0.115 | <0.001 | 0.08 | <0.001 | 0.211 | <0.001 | -0.088 | <0.001 | -0.129 | <0.001 | -0.009 | 0.358 | -0.155 | <0.001 |
| PI3K_AKT_MTOR_SIGNALING | -0.185 | <0.001 | 0.074 | <0.001 | 0.286 | <0.001 | 0.182 | <0.001 | 0.076 | <0.001 | 0.117 | <0.001 | 0.034 | 0.001 | -0.06 | <0.001 |
| PROTEIN_SECRETION | -0.063 | <0.001 | -0.047 | <0.001 | 0.207 | <0.001 | 0.261 | <0.001 | 0.402 | <0.001 | 0.099 | <0.001 | 0.254 | <0.001 | 0.111 | <0.001 |
| REACTIVE_OXYGEN_SPECIES_PATHWAY | 0.193 | <0.001 | 0.051 | <0.001 | 0.178 | <0.001 | 0.199 | <0.001 | -0.182 | <0.001 | 0.11 | <0.001 | 0.026 | 0.008 | -0.174 | <0.001 |
| SPERMATOGENESIS | -0.119 | <0.001 | 0.103 | <0.001 | -0.217 | <0.001 | -0.426 | <0.001 | -0.207 | <0.001 | -0.343 | <0.001 | -0.354 | <0.001 | 0.282 | <0.001 |
| TGF_BETA_SIGNALING | -0.006 | 0.532 | 0.028 | 0.005 | 0.391 | <0.001 | 0.4 | <0.001 | 0.487 | <0.001 | 0.383 | <0.001 | 0.417 | <0.001 | 0.121 | <0.001 |
| TNFA_SIGNALING_VIA_NFKB | 0.09 | <0.001 | -0.018 | 0.074 | 0.399 | <0.001 | 0.372 | <0.001 | 0.16 | <0.001 | 0.319 | <0.001 | 0.258 | <0.001 | -0.137 | <0.001 |
| UNFOLDED_PROTEIN_RESPONSE | -0.155 | <0.001 | 0.286 | <0.001 | 0.113 | <0.001 | 0.051 | <0.001 | -0.062 | <0.001 | -0.112 | <0.001 | -0.299 | <0.001 | 0.004 | 0.652 |
| UV_RESPONSE_DN | 0.051 | <0.001 | -0.018 | 0.073 | 0.345 | <0.001 | 0.417 | <0.001 | 0.601 | <0.001 | 0.296 | <0.001 | 0.492 | <0.001 | 0.143 | <0.001 |
| UV_RESPONSE_UP | 0.142 | <0.001 | 0.25 | <0.001 | 0.257 | <0.001 | 0.136 | <0.001 | -0.057 | <0.001 | 0.042 | <0.001 | -0.041 | <0.001 | 0.033 | 0.001 |
| WNT_BETA_CATENIN_SIGNALING | -0.104 | <0.001 | 0.195 | <0.001 | 0.098 | <0.001 | -0.009 | 0.382 | 0.193 | <0.001 | 0.119 | <0.001 | 0.029 | 0.003 | 0.194 | <0.001 |
| XENOBIOTIC_METABOLISM | 0.498 | <0.001 | 0.025 | 0.012 | 0.261 | <0.001 | 0.373 | <0.001 | -0.039 | <0.001 | 0.065 | <0.001 | 0.197 | <0.001 | -0.187 | <0.001 |

| **Table S5 Correlation between IGFBPs expression and immune cell infiltration in pan-cancer** | | | | |
| --- | --- | --- | --- | --- |
| **Gene** | **Cancer Type** | **Cell Type** | **Correlation Coefficient** | ***P* value** |
| IGFBP1 | UVM | NK cells resting | 0.39 | 0.026 |
| IGFBP1 | ACC | Eosinophils | 0.38 | 0.017 |
| IGFBP1 | CHOL | T cells CD4 memory resting | 0.37 | 0.037 |
| IGFBP1 | CHOL | B cells naive | 0.35 | 0.043 |
| IGFBP1 | PCPG | NK cells resting | 0.35 | 0.003 |
| IGFBP1 | UCS | T cells CD4 memory activated | 0.34 | 0.034 |
| IGFBP1 | DLBC | T cells CD4 memory activated | 0.31 | 0.031 |
| IGFBP1 | TGCT | Macrophages M2 | 0.3 | 0.000 |
| IGFBP1 | PCPG | T cells follicular helper | -0.33 | 0.005 |
| IGFBP1 | DLBC | Dendritic cells resting | -0.37 | 0.010 |
| IGFBP1 | KICH | T cells CD8 | -0.38 | 0.023 |
| IGFBP1 | ACC | Mast cells resting | -0.39 | 0.012 |
| IGFBP2 | LGG | Macrophages M0 | 0.35 | <0.001 |
| IGFBP2 | DLBC | B cells memory | 0.34 | 0.017 |
| IGFBP2 | LAML | Mast cells resting | 0.34 | <0.001 |
| IGFBP2 | UCS | T cells CD4 memory activated | -0.32 | 0.046 |
| IGFBP2 | DLBC | T cells follicular helper | -0.32 | 0.026 |
| IGFBP2 | KIRP | Macrophages M2 | -0.34 | <0.001 |
| IGFBP2 | KICH | Monocytes | -0.34 | 0.044 |
| IGFBP2 | PCPG | NK cells activated | -0.35 | 0.003 |
| IGFBP2 | LGG | Monocytes | -0.39 | <0.001 |
| IGFBP3 | PCPG | Dendritic cells activated | 0.39 | <0.001 |
| IGFBP3 | TGCT | Dendritic cells activated | 0.39 | <0.001 |
| IGFBP3 | LAML | Plasma cells | 0.39 | <0.001 |
| IGFBP3 | ACC | T cells follicular helper | 0.37 | 0.018 |
| IGFBP3 | KICH | Dendritic cells activated | 0.36 | 0.031 |
| IGFBP3 | PCPG | NK cells resting | 0.36 | 0.002 |
| IGFBP3 | THYM | Macrophages M2 | 0.35 | <0.001 |
| IGFBP3 | THYM | NK cells activated | 0.34 | <0.001 |
| IGFBP3 | SARC | Macrophages M2 | 0.32 | <0.001 |
| IGFBP3 | TGCT | Mast cells resting | 0.32 | <0.001 |
| IGFBP3 | THYM | Mast cells resting | 0.32 | <0.001 |
| IGFBP3 | DLBC | Macrophages M0 | 0.31 | 0.033 |
| IGFBP3 | KIRP | Macrophages M2 | -0.31 | <0.001 |
| IGFBP3 | THYM | NK cells resting | -0.31 | <0.001 |
| IGFBP3 | DLBC | T cells regulatory (Tregs) | -0.31 | 0.035 |
| IGFBP3 | UCS | B cells naive | -0.33 | 0.041 |
| IGFBP3 | KICH | T cells regulatory (Tregs) | -0.33 | 0.050 |
| IGFBP3 | THYM | Dendritic cells resting | -0.36 | <0.001 |
| IGFBP3 | PCPG | NK cells activated | -0.39 | <0.001 |
| IGFBP4 | LGG | Macrophages M0 | 0.39 | <0.001 |
| IGFBP4 | KICH | T cells CD8 | 0.37 | 0.026 |
| IGFBP4 | KICH | Macrophages M2 | 0.36 | 0.031 |
| IGFBP4 | ACC | Plasma cells | 0.36 | 0.022 |
| IGFBP4 | KICH | Plasma cells | 0.36 | 0.033 |
| IGFBP4 | STAD | Mast cells resting | 0.35 | <0.001 |
| IGFBP4 | ACC | T cells regulatory (Tregs) | 0.34 | 0.029 |
| IGFBP4 | PAAD | B cells naive | 0.33 | <0.001 |
| IGFBP4 | GBM | Neutrophils | 0.31 | <0.001 |
| IGFBP4 | MESO | T cells CD4 memory activated | 0.31 | 0.004 |
| IGFBP4 | LAML | Plasma cells | 0.3 | <0.001 |
| IGFBP4 | KICH | Dendritic cells activated | -0.33 | 0.050 |
| IGFBP4 | PCPG | T cells CD4 memory resting | -0.33 | 0.006 |
| IGFBP4 | KICH | Mast cells resting | -0.34 | 0.043 |
| IGFBP4 | CHOL | Neutrophils | -0.36 | 0.039 |
| IGFBP4 | PRAD | Macrophages M1 | -0.38 | <0.001 |
| IGFBP4 | ACC | Monocytes | -0.39 | 0.013 |
| IGFBP5 | STAD | Mast cells resting | 0.38 | <0.001 |
| IGFBP5 | PRAD | T cells CD4 memory resting | 0.38 | <0.001 |
| IGFBP5 | KIRP | Macrophages M1 | 0.37 | <0.001 |
| IGFBP5 | CESC | B cells naive | 0.36 | <0.001 |
| IGFBP5 | PCPG | B cells naive | 0.36 | 0.002 |
| IGFBP5 | ESCA | Mast cells resting | 0.36 | <0.001 |
| IGFBP5 | HNSC | B cells naive | 0.34 | <0.001 |
| IGFBP5 | BLCA | B cells naive | 0.33 | <0.001 |
| IGFBP5 | UCS | Macrophages M2 | 0.32 | 0.045 |
| IGFBP5 | SARC | Mast cells resting | 0.32 | <0.001 |
| IGFBP5 | KIRP | Macrophages M2 | -0.3 | <0.001 |
| IGFBP5 | KIRP | B cells memory | -0.31 | <0.001 |
| IGFBP5 | UCS | Mast cells activated | -0.32 | 0.045 |
| IGFBP5 | ACC | Monocytes | -0.32 | 0.043 |
| IGFBP5 | PCPG | Dendritic cells activated | -0.36 | 0.002 |
| IGFBP5 | UCS | T cells CD8 | -0.36 | 0.025 |
| IGFBP6 | UCS | Mast cells resting | 0.39 | 0.015 |
| IGFBP6 | LGG | T cells CD8 | 0.39 | <0.001 |
| IGFBP6 | UCS | NK cells activated | 0.37 | 0.021 |
| IGFBP6 | STAD | Mast cells resting | 0.36 | <0.001 |
| IGFBP6 | ACC | T cells CD4 memory activated | 0.36 | 0.024 |
| IGFBP6 | TGCT | T cells CD4 memory resting | 0.36 | <0.001 |
| IGFBP6 | THCA | Dendritic cells resting | 0.35 | <0.001 |
| IGFBP6 | UVM | Monocytes | 0.35 | 0.046 |
| IGFBP6 | ACC | T cells follicular helper | 0.34 | 0.032 |
| IGFBP6 | KIRC | T cells CD8 | 0.33 | <0.001 |
| IGFBP6 | DLBC | Macrophages M0 | 0.32 | 0.028 |
| IGFBP6 | STAD | Monocytes | 0.32 | <0.001 |
| IGFBP6 | KIRP | Mast cells resting | 0.31 | <0.001 |
| IGFBP6 | LAML | T cells CD8 | 0.3 | 0.000 |
| IGFBP6 | DLBC | Dendritic cells activated | -0.3 | 0.037 |
| IGFBP6 | GBM | Macrophages M2 | -0.3 | <0.001 |
| IGFBP6 | KIRP | T cells follicular helper | -0.3 | <0.001 |
| IGFBP6 | LGG | Monocytes | -0.31 | <0.001 |
| IGFBP6 | GBM | T cells follicular helper | -0.33 | <0.001 |
| IGFBP6 | THYM | T cells follicular helper | -0.33 | <0.001 |
| IGFBP6 | ESCA | T cells regulatory (Tregs) | -0.34 | <0.001 |
| IGFBP6 | UVM | Dendritic cells resting | -0.35 | 0.049 |
| IGFBP6 | ACC | T cells CD4 memory resting | -0.35 | 0.028 |
| IGFBP6 | THYM | B cells naive | -0.36 | <0.001 |
| IGFBP6 | PRAD | Macrophages M1 | -0.38 | <0.001 |
| IGFBP7 | ACC | Plasma cells | 0.38 | 0.016 |
| IGFBP7 | STAD | Mast cells resting | 0.37 | <0.001 |
| IGFBP7 | KICH | T cells CD4 memory resting | 0.37 | 0.028 |
| IGFBP7 | READ | Eosinophils | 0.36 | <0.001 |
| IGFBP7 | READ | Macrophages M2 | 0.36 | <0.001 |
| IGFBP7 | THYM | Mast cells resting | 0.36 | <0.001 |
| IGFBP7 | KICH | T cells CD8 | 0.36 | 0.032 |
| IGFBP7 | READ | Dendritic cells resting | 0.35 | <0.001 |
| IGFBP7 | ACC | T cells regulatory (Tregs) | 0.35 | 0.028 |
| IGFBP7 | COAD | Macrophages M2 | 0.34 | <0.001 |
| IGFBP7 | STAD | Monocytes | 0.34 | <0.001 |
| IGFBP7 | ESCA | Macrophages M2 | 0.33 | <0.001 |
| IGFBP7 | LAML | B cells memory | 0.31 | <0.001 |
| IGFBP7 | DLBC | Mast cells resting | -0.3 | 0.038 |
| IGFBP7 | MESO | NK cells activated | -0.31 | 0.004 |
| IGFBP7 | LGG | Mast cells activated | -0.32 | <0.001 |
| IGFBP7 | THYM | T cells follicular helper | -0.34 | <0.001 |
| IGFBP7 | ACC | Dendritic cells activated | -0.39 | 0.012 |
| IGFBPL1 | ACC | Eosinophils | 0.35 | 0.026 |
| IGFBPL1 | ACC | T cells follicular helper | 0.32 | 0.041 |
| IGFBPL1 | KIRP | Mast cells resting | -0.3 | <0.001 |

**Table S6 Correlation between IGFBPs CNV and IGFBPs expression**

| gene | CancerType | CNV | n | Expression median value | p value |
| --- | --- | --- | --- | --- | --- |
| IGFBP3 | CESC | DEL | 3 | 11.075(10.406-11.513) | 0.018 |
|  | CESC | GAIN | 1 | 16.022(16.022-16.022) |  |
|  | CESC | No Change | 290 | 13.43(12.194-14.806) |  |
|  | LIHC | DEL | 3 | 10.321(10.248-10.562) | 0.03 |
|  | LIHC | GAIN | 4 | 12.877(12.386-13.385) |  |
|  | LIHC | No Change | 365 | 12.327(11.431-13.291) |  |
|  | OV | DEL | 18 | 12.624(11.846-13.494) | 0.017 |
|  | OV | GAIN | 30 | 13.876(12.85-14.581) |  |
|  | OV | No Change | 329 | 13.262(12.544-13.963) |  |
|  | UCEC | DEL | 5 | 12.422(11.82-12.849) | 0.043 |
|  | UCEC | GAIN | 10 | 14.204(13.37-14.616) |  |
|  | UCEC | No Change | 525 | 13.126(12.38-14.11) |  |
| IGFBP4 | GBM | DEL | 3 | 11.418(11.18-11.51) | 0.046 |
|  | GBM | GAIN | 1 | 13.289(13.289-13.289) |  |
|  | GBM | No Change | 160 | 12.243(11.733-12.829) |  |
|  | SARC | DEL | 25 | 14.856(13.966-15.342) | 0.041 |
|  | SARC | GAIN | 11 | 16.056(15.192-16.547) |  |
|  | SARC | No Change | 226 | 15.203(14.458-15.909) |  |
|  | UCEC | DEL | 32 | 11.977(11.148-12.702) | 0.003 |
|  | UCEC | GAIN | 20 | 12.831(12.323-13.473) |  |
|  | UCEC | No Change | 488 | 12.831(11.914-13.733) |  |
| IGFBP5 | BRCA | DEL | 48 | 14.403(13.48-15.27) | 0.008 |
|  | BRCA | GAIN | 35 | 15.346(13.404-16.448) |  |
|  | BRCA | No Change | 1007 | 15.131(14.049-16.368) |  |
|  | CESC | DEL | 71 | 10.937(10-12.484) | 0.01 |
|  | CESC | GAIN | 3 | 9.83(8.898-12.294) |  |
|  | CESC | No Change | 220 | 12.067(10.569-13.635) |  |
|  | MESO | DEL | 8 | 13.843(12.879-14.361) | 0.036717862 |
|  | MESO | No Change | 78 | 14.611(13.646-15.817) |  |
|  | OV | DEL | 55 | 13.682(12.513-14.604) | 0.02 |
|  | OV | GAIN | 51 | 14.341(13.799-15.197) |  |
|  | OV | No Change | 271 | 14.081(13.061-15.082) |  |
|  | PRAD | DEL | 9 | 13.362(12.759-13.915) | 0.027 |
|  | PRAD | GAIN | 5 | 14.803(14.232-15.226) |  |
|  | PRAD | No Change | 482 | 14.429(13.718-15.106) |  |
|  | SARC | DEL | 32 | 15.129(13.932-16.086) | 0.042 |
|  | SARC | GAIN | 11 | 16.712(15.601-17.173) |  |
|  | SARC | No Change | 219 | 15.63(13.96-16.757) |  |
|  | THCA | DEL | 2 | 13.796(13.598-13.994) | 0.05 |
|  | THCA | GAIN | 1 | 13.114(13.114-13.114) |  |
|  | THCA | No Change | 504 | 16.41(15.11-17.63) |  |
| IGFBP6 | BRCA | DEL | 50 | 7.997(7.26-8.766) | < 0.001 |
|  | BRCA | GAIN | 31 | 8.2(7.986-9.236) |  |
|  | BRCA | No Change | 1009 | 8.738(7.919-9.527) |  |
|  | LIHC | DEL | 3 | 8(7.489-8.884) | 0.02 |
|  | LIHC | GAIN | 8 | 5.807(5.034-6.535) |  |
|  | LIHC | No Change | 361 | 6.615(5.807-7.658) |  |
|  | LUSC | DEL | 15 | 7.864(6.927-8.556) | 0.014 |
|  | LUSC | GAIN | 16 | 8.939(8.082-10.479) |  |
|  | LUSC | No Change | 469 | 8.728(7.807-9.687) |  |
|  | MESO | DEL | 3 | 10.603(9.519-10.854) | 0.018592412 |
|  | MESO | No Change | 83 | 12.432(11.419-13.203) |  |
|  | SARC | DEL | 20 | 9.469(8.282-10.309) | 0.006 |
|  | SARC | GAIN | 10 | 11.428(10.587-12.72) |  |
|  | SARC | No Change | 232 | 11.217(9.394-12.723) |  |
|  | UCEC | DEL | 4 | 8.876(8.202-9.246) | < 0.001 |
|  | UCEC | GAIN | 32 | 9.03(7.615-10.697) |  |
|  | UCEC | No Change | 504 | 7.687(6.883-8.568) |  |
| IGFBP7 | BRCA | DEL | 10 | 13.872(13.302-14.354) | 0.014 |
|  | BRCA | GAIN | 75 | 13.748(13.056-14.131) |  |
|  | BRCA | No Change | 1005 | 14.028(13.453-14.527) |  |
|  | LUAD | DEL | 12 | 13.015(11.901-13.43) | 0.016 |
|  | LUAD | GAIN | 29 | 13.111(12.027-13.708) |  |
|  | LUAD | No Change | 483 | 13.392(12.733-14.117) |  |
| IGFBPL1 | BRCA | DEL | 33 | 3.459(1.585-5.524) | < 0.001 |
|  | BRCA | GAIN | 48 | 5.066(3.284-6.45) |  |
|  | BRCA | No Change | 1009 | 3.585(2-5.044) |  |
|  | HNSC | DEL | 26 | 2.161(0-3.284) | 0.026 |
|  | HNSC | GAIN | 20 | 4.085(1.896-6.13) |  |
|  | HNSC | No Change | 450 | 2.322(1-3.907) |  |
|  | LUAD | DEL | 22 | 4.614(3.459-5.785) | < 0.001 |
|  | LUAD | GAIN | 31 | 7.276(5.243-8.751) |  |
|  | LUAD | No Change | 471 | 5.977(4.524-7.317) |  |
|  | LUSC | DEL | 19 | 4.392(3.7-5.489) | 0.018 |
|  | LUSC | GAIN | 56 | 5.875(4.508-7.165) |  |
|  | LUSC | No Change | 425 | 5.044(3.585-6.267) |  |
|  | SARC | DEL | 11 | 3.322(0-5.978) | < 0.001 |
|  | SARC | GAIN | 20 | 0(0-1.769) |  |
|  | SARC | No Change | 231 | 4(1-7.96) |  |
|  | STAD | DEL | 10 | 4.751(3.227-6.422) | < 0.001 |
|  | STAD | GAIN | 23 | 7.931(6.363-9.341) |  |
|  | STAD | No Change | 340 | 4.907(3-6.839) |  |

| **Table S7 The effects of IGFBPs on cancer** | | | | | |
| --- | --- | --- | --- | --- | --- |
| **Gene** | **Effect** | **Cancer** | **Influence** | **Title** | **Ref.** |
| IGFBP1 | apoptosis | hepatocarcinoma | Resist apoptosis | Effects of natural 24-epibrassinolide on inducing apoptosis and restricting metabolism in hepatocarcinoma cells. | (1) |
| IGFBP1 | bioenergetics and metabolism | colorectal cancer | Excess energy balance leads to depressed levels of circulating IGFBP-1, and promotes cancer cell growth | Insulin, the insulin-like growth factor axis, and mortality in patients with nonmetastatic colorectal cancer. | (2) |
| IGFBP1 | bioenergetics and metabolism | hepatocarcinoma | Secreted IGFBP1 affected cell energy metabolism by inhibiting the phosphorylation of Akt | Effects of natural 24-epibrassinolide on inducing apoptosis and restricting metabolism in hepatocarcinoma cells. | (1) |
| IGFBP1 | metastasis | Colorectal Cancer | Promote cancer metastasis | Complex Behavior of ALDH1A1 and IGFBP1 in Liver Metastasis from a Colorectal Cancer | (3) |
| IGFBP1 | metastasis | Gastric Cancer | High IGFBP1 expression was associated with haematogenous metastasis | IGFBP1 Is a Predictive Factor for Haematogenous Metastasis in Patients With Gastric Cancer. | (4) |
| IGFBP2 | apoptosis | malignant melanoma | Downregulation of CD147 induces malignant melanoma cell apoptosis via the regulation of IGFBP2 expression | Downregulation of CD147 induces malignant melanoma cell apoptosis via the regulation of IGFBP2 expression. | (5) |
| IGFBP2 | bioenergetics and metabolism | Cancer | IGFBP-2 administration can be beneficial in improving metabolic responses | IGFBP-2 - taking the lead in growth, metabolism and cancer | (6) |
| IGFBP2 | metastasis | Breast Cancer | Promote cancer metastasis | Astrocytic IGFBP2 and CHI3L1 in cerebrospinal fluid drive cortical metastasis of HER2+breast cancer. | (7) |
| IGFBP2 | metastasis | Oral Cancer | IGFBP-2 participated in oral cancer metastasis | Role of IGFBP-2 in oral cancer metastasis. | (8) |
| IGFBP3 | apoptosis | Gastric Cancer | IGFBP-3 exhibits anti-apoptotic effects | Expression and underlying roles of IGFBP-3 in paclitaxel-treated gastric cancer SGC-7901 cells. | (9) |
| IGFBP3 | apoptosis | Hepatocellular Carcinoma | Promote apoptosis | IGFBP-3 Is the Key Target of Sanguinarine in Promoting Apoptosis in Hepatocellular Carcinoma. | (10) |
| IGFBP3 | apoptosis | Gallbladder cancer | The miR-197-IGFBP3 axis regulates the apoptosis of GBC cells. | MicroRNA-197 promotes proliferation and inhibits apoptosis of gallbladder cancer cells by targeting insulin-like growth factor-binding protein 3. | (11) |
| IGFBP3 | bioenergetics and metabolism | Breast Cancer | Energy homeostasis genes interact with IGFBP-3 on the risk of breast cancer | Energy homeostasis genes modify the association between serum concentrations of IGF-1 and IGFBP-3 and breast cancer risk | (12) |
| IGFBP3 | metastasis | Colorectal Cancer | IGFBP3 and 7 play a special role in cancer metastasis | The insulin-like growth factor binding proteins 3 and 7 are associated with colorectal cancer and liver metastasis. | (13) |
| IGFBP3 | metastasis | Cancer | IGFBP-3/IGFBP-3 Receiver System plays an anti-Metastatic role | IGFBP-3/IGFBP-3 Receptor System as an Anti-Tumor and Anti-Metastatic Signaling in Cancer | (14) |
| IGFBP4 | apoptosis | Breast Cancer | Promote apoptosis | IGFBP-3: a cell fate pivot in cancer and disease | (15) |
| IGFBP4 | apoptosis | Skin Cutaneous Melanoma | Promote apoptosis | PSMC2 knockdown suppressed tumor progression of skin cutaneous melanoma. | (16) |
| IGFBP5 | apoptosis | Breast Cancer | IGFBP-3 could enhance induced apoptosis | Interaction between GRP78 and IGFBP-3 Affects Tumourigenesis and Prognosis in Breast Cancer Patients. | (17) |
| IGFBP5 | bioenergetics and metabolism | Breast Cancer | Osteopontin-a upregulates the levels of glucose in breast cancer cells through IGFBP5 | Osteopontin-a alters glucose homeostasis in anchorage-independent breast cancer cells. | (18) |
| IGFBP5 | bioenergetics and metabolism | Melanoma | Affects glycolysis | Enhancer Reprogramming Confers Dependence on Glycolysis and IGF Signaling in KMT2D Mutant Melanoma. | (19) |
| IGFBP5 | metastasis | hepatocellular carcinoma | IGFBP-1 inhibits the invasion and metastasis of HCC cells | Insulin-like growth factor binding protein-1 inhibits cancer cell invasion and is associated with poor prognosis in hepatocellular carcinoma | (20) |
| IGFBP6 | apoptosis | Glioma | IGFBP6 Regulates Cell Apoptosis and Migration in Glioma | IGFBP6 Regulates Cell Apoptosis and Migration in Glioma. | (21) |
| IGFBP6 | apoptosis | Colorectal Cancer | Promote apoptosis | Correlation of IGFBP-6 expression with apoptosis and migration of colorectal carcinoma cells. | (22) |
| IGFBP6 | bioenergetics and metabolism | Colorectal Cancer | IGFBP-6 may be a potential biomarker associated with lymphogenous metastasis in CRC | Association of IGFBP-6 Expression with Metabolic Syndrome and Adiponectin and IGF-IR Receptor Levels in Colorectal Cancer | (23) |
| IGFBP6 | bioenergetics and metabolism | Breast Cancer | GPR81/IGFBP6 Promotes Breast Cancer Progression by Modulating Lactate Metabolism and Oxidative Stress | The Crosstalk between GPR81/IGFBP6 Promotes Breast Cancer Progression by Modulating Lactate Metabolism and Oxidative Stress. | (24) |
| IGFBP6 | metastasis | Colorectal Cancer | IGFBP-6 may be a potential biomarker associated with the metabolic syndrome in CRC | Association of IGFBP-6 Expression with Metabolic Syndrome and Adiponectin and IGF-IR Receptor Levels in Colorectal Cancer | (23) |
| IGFBP6 | metastasis | head and neck squamous cell carcinoma | IGFBP6 is down-regulated in metastatic HNSCC | Differential gene expression signature between primary and metastatic head and neck squamous cell carcinoma. | (25) |
| IGFBP6 | metastasis | Breast Cancer | IGFBP6 is differentially expressed in bone metastasis in breast cancer (BC) patients | Nomogram Models Based on the Gene Expression in Prediction of Breast Cancer Bone Metastasis. | (26) |
| IGFBP7 | apoptosis | Hepatocellular Carcinoma | Promote apoptosis | IGFBP7 Deletion Promotes Hepatocellular Carcinoma. | (27) |
| IGFBP7 | apoptosis | Gastric Cancer | IGFBP7 overexpression promotes apoptosis | Epigenetic Downregulation and Growth Inhibition of IGFBP7 in Gastric Cancer. | (28) |
| IGFBP7 | metastasis | Colorectal Cancer | IGFBP3 and 7 play a special role in cancer metastasis | The insulin-like growth factor binding proteins 3 and 7 are associated with colorectal cancer and liver metastasis. | (13) |
| IGFBP7 | metastasis | Soft-tissue sarcomas | Overexpression of IGFBP7 increases the risk of metastasis | Tissue and serum IGFBP7 protein as biomarker in high-grade soft tissue sarcoma. | (29) |

**Reference**

1. Zhou H, Zhuang W, Huang H, Ma N, Lei J, Jin G, et al. Effects of natural 24-epibrassinolide on inducing apoptosis and restricting metabolism in hepatocarcinoma cells. Phytomedicine. 2022;107:154428.

2. Wolpin BM, Meyerhardt JA, Chan AT, Ng K, Chan JA, Wu K, et al. Insulin, the insulin-like growth factor axis, and mortality in patients with nonmetastatic colorectal cancer. J Clin Oncol. 2009;27(2):176-85.

3. Kim JC, Ha YJ, Tak KH, Roh SA, Kim CW, Kim TW, et al. Complex Behavior of ALDH1A1 and IGFBP1 in Liver Metastasis from a Colorectal Cancer. PLoS One. 2016;11(5):e0155160.

4. Sato Y, Inokuchi M, Takagi Y, Kojima K. IGFBP1 Is a Predictive Factor for Haematogenous Metastasis in Patients With Gastric Cancer. Anticancer Res. 2019;39(6):2829-37.

5. Zhao S, Wu L, Kuang Y, Su J, Luo Z, Wang Y, et al. Downregulation of CD147 induces malignant melanoma cell apoptosis via the regulation of IGFBP2 expression. Int J Oncol. 2018;53(6):2397-408.

6. Yau SW, Azar WJ, Sabin MA, Werther GA, Russo VC. IGFBP-2 - taking the lead in growth, metabolism and cancer. J Cell Commun Signal. 2015;9(2):125-42.

7. Ansari KI, Bhan A, Liu X, Chen MY, Jandial R. Astrocytic IGFBP2 and CHI3L1 in cerebrospinal fluid drive cortical metastasis of HER2+breast cancer. Clin Exp Metastasis. 2020;37(3):401-12.

8. Tsai YF, Chou HC, Liou MH, Liao EC, Cheng CT, Chang SJ, et al. Role of IGFBP-2 in oral cancer metastasis. Biochim Biophys Acta Mol Basis Dis. 2021;1867(7):166143.

9. Huang G, Dang ZF, Dang YM, Cai W, Li Y, Chen YR, et al. Expression and underlying roles of IGFBP-3 in paclitaxel-treated gastric cancer SGC-7901 cells. Asian Pac J Cancer Prev. 2014;15(14):5741-5.

10. Wang H, Wang H, Li K, Li S, Sun B. IGFBP-3 Is the Key Target of Sanguinarine in Promoting Apoptosis in Hepatocellular Carcinoma. Cancer Manag Res. 2020;12:1007-15.

11. Tong L, Cheng J, Zuo H, Li J. MicroRNA-197 promotes proliferation and inhibits apoptosis of gallbladder cancer cells by targeting insulin-like growth factor-binding protein 3. Adv Clin Exp Med. 2021;30(7):661-72.

12. Rodriguez-Valentin R, Torres-Mejia G, Martinez-Matsushita L, Angeles-Llerenas A, Gomez-Flores-Ramos L, Wolff RK, et al. Energy homeostasis genes modify the association between serum concentrations of IGF-1 and IGFBP-3 and breast cancer risk. Sci Rep. 2022;12(1):1837.

13. Georges RB, Adwan H, Hamdi H, Hielscher T, Linnemann U, Berger MR. The insulin-like growth factor binding proteins 3 and 7 are associated with colorectal cancer and liver metastasis. Cancer Biol Ther. 2011;12(1):69-79.

14. Cai Q, Dozmorov M, Oh Y. IGFBP-3/IGFBP-3 Receptor System as an Anti-Tumor and Anti-Metastatic Signaling in Cancer. Cells. 2020;9(5).

15. Johnson MA, Firth SM. IGFBP-3: a cell fate pivot in cancer and disease. Growth Horm IGF Res. 2014;24(5):164-73.

16. Yang Y, Qi F, Wei C, Liu J, Zhang Y, Luan W, et al. PSMC2 knockdown suppressed tumor progression of skin cutaneous melanoma. Cell Death Discov. 2021;7(1):323.

17. Zielinska HA, Daly CS, Alghamdi A, Bahl A, Sohail M, White P, et al. Interaction between GRP78 and IGFBP-3 Affects Tumourigenesis and Prognosis in Breast Cancer Patients. Cancers (Basel). 2020;12(12).

18. Shi Z, Mirza M, Wang B, Kennedy MA, Weber GF. Osteopontin-a alters glucose homeostasis in anchorage-independent breast cancer cells. Cancer Lett. 2014;344(1):47-53.

19. Maitituoheti M, Keung EZ, Tang M, Yan L, Alam H, Han G, et al. Enhancer Reprogramming Confers Dependence on Glycolysis and IGF Signaling in KMT2D Mutant Melanoma. Cell Rep. 2020;33(3):108293.

20. Dai B, Ruan B, Wu J, Wang J, Shang R, Sun W, et al. Insulin-like growth factor binding protein-1 inhibits cancer cell invasion and is associated with poor prognosis in hepatocellular carcinoma. Int J Clin Exp Pathol. 2014;7(9):5645-54.

21. Bei Y, Huang Q, Shen J, Shi J, Shen C, Xu P, et al. IGFBP6 Regulates Cell Apoptosis and Migration in Glioma. Cell Mol Neurobiol. 2017;37(5):889-98.

22. Qiu F, Gao W, Wang B. Correlation of IGFBP-6 expression with apoptosis and migration of colorectal carcinoma cells. Cancer Biomark. 2018;21(4):893-8.

23. Yunusova NV, Spirina LV, Frolova AE, Afanas'ev SG, Kolegova ES, Kondakova IV. Association of IGFBP-6 Expression with Metabolic Syndrome and Adiponectin and IGF-IR Receptor Levels in Colorectal Cancer. Asian Pac J Cancer Prev. 2016;17(8):3963-9.

24. Longhitano L, Forte S, Orlando L, Grasso S, Barbato A, Vicario N, et al. The Crosstalk between GPR81/IGFBP6 Promotes Breast Cancer Progression by Modulating Lactate Metabolism and Oxidative Stress. Antioxidants (Basel). 2022;11(2).

25. Liu CJ, Liu TY, Kuo LT, Cheng HW, Chu TH, Chang KW, et al. Differential gene expression signature between primary and metastatic head and neck squamous cell carcinoma. J Pathol. 2008;214(4):489-97.

26. Fan TD, Bei DK, Li SW. Nomogram Models Based on the Gene Expression in Prediction of Breast Cancer Bone Metastasis. J Healthc Eng. 2022;2022:8431946.

27. Akiel M, Guo C, Li X, Rajasekaran D, Mendoza RG, Robertson CL, et al. IGFBP7 Deletion Promotes Hepatocellular Carcinoma. Cancer Res. 2017;77(15):4014-25.

28. Kim J, Kim WH, Byeon SJ, Lee BL, Kim MA. Epigenetic Downregulation and Growth Inhibition of IGFBP7 in Gastric Cancer. Asian Pac J Cancer Prev. 2018;19(3):667-75.

29. Benassi MS, Pazzaglia L, Novello C, Quattrini I, Pollino S, Magagnoli G, et al. Tissue and serum IGFBP7 protein as biomarker in high-grade soft tissue sarcoma. Am J Cancer Res. 2015;5(11):3446-54.
