# Supplementary material for: Expression characteristics and their functional role of IGFBP gene family in pan-cancer
Source: BMC Cancer. 2023 Apr 24;23:371. doi: 10.1186/s12885-023-10832-3 (PMC10124011; doi:10.1186/s12885-023-10832-3)
Supplement: Supplementary file 2 — Additional file 2: Figure S1. The kaplan-meier survival curve plots for the IGFBP expression on overall survival in pan-cancer. Figure S2. The correlation between IGFBPs expression in 24 types of cancers from TCGA. Figure S3. The correlation between IGFBPs expression and (A) TMB and (B) MSI in pan-cancer. Figure S4. The mutation details of IGFBPs in pan-cancer. [file 12885_2023_10832_MOESM2_ESM.docx]

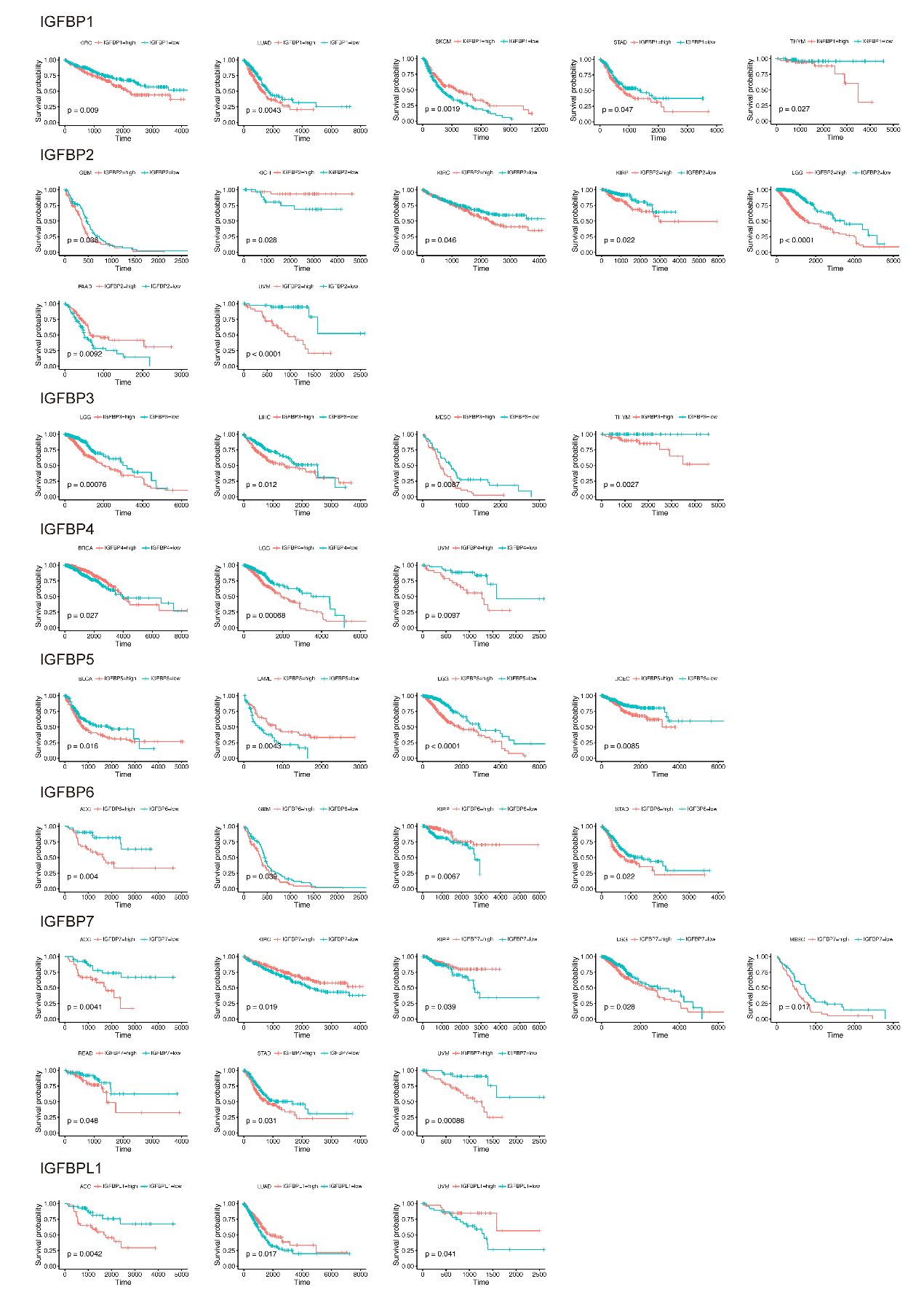


Figure S1. The kaplan-meier survival curve plots for the IGFBP expression on overall survival in pan-cancer.


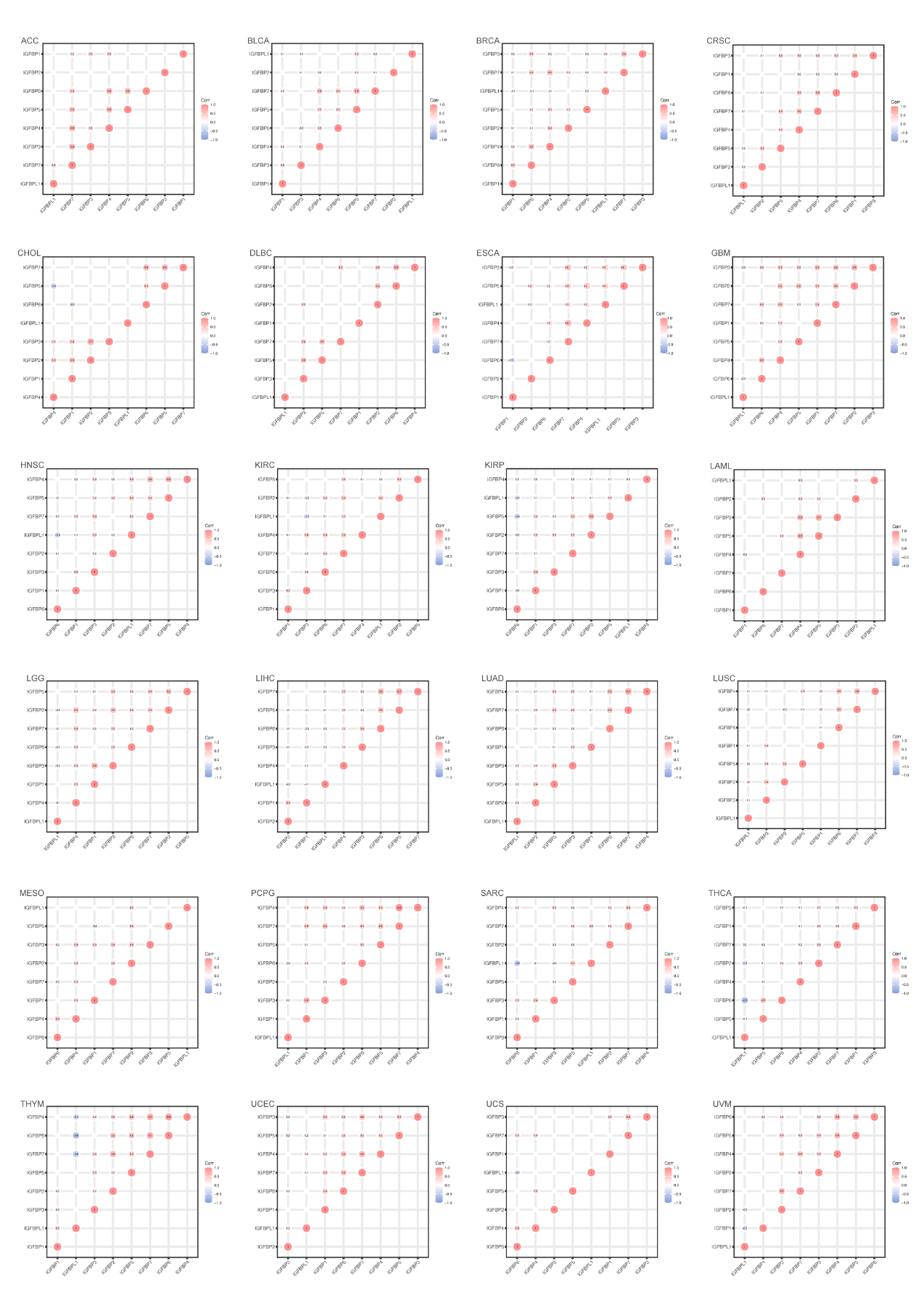


Figure S2. The correlation between IGFBPs expression in 24 types of cancers from TCGA.


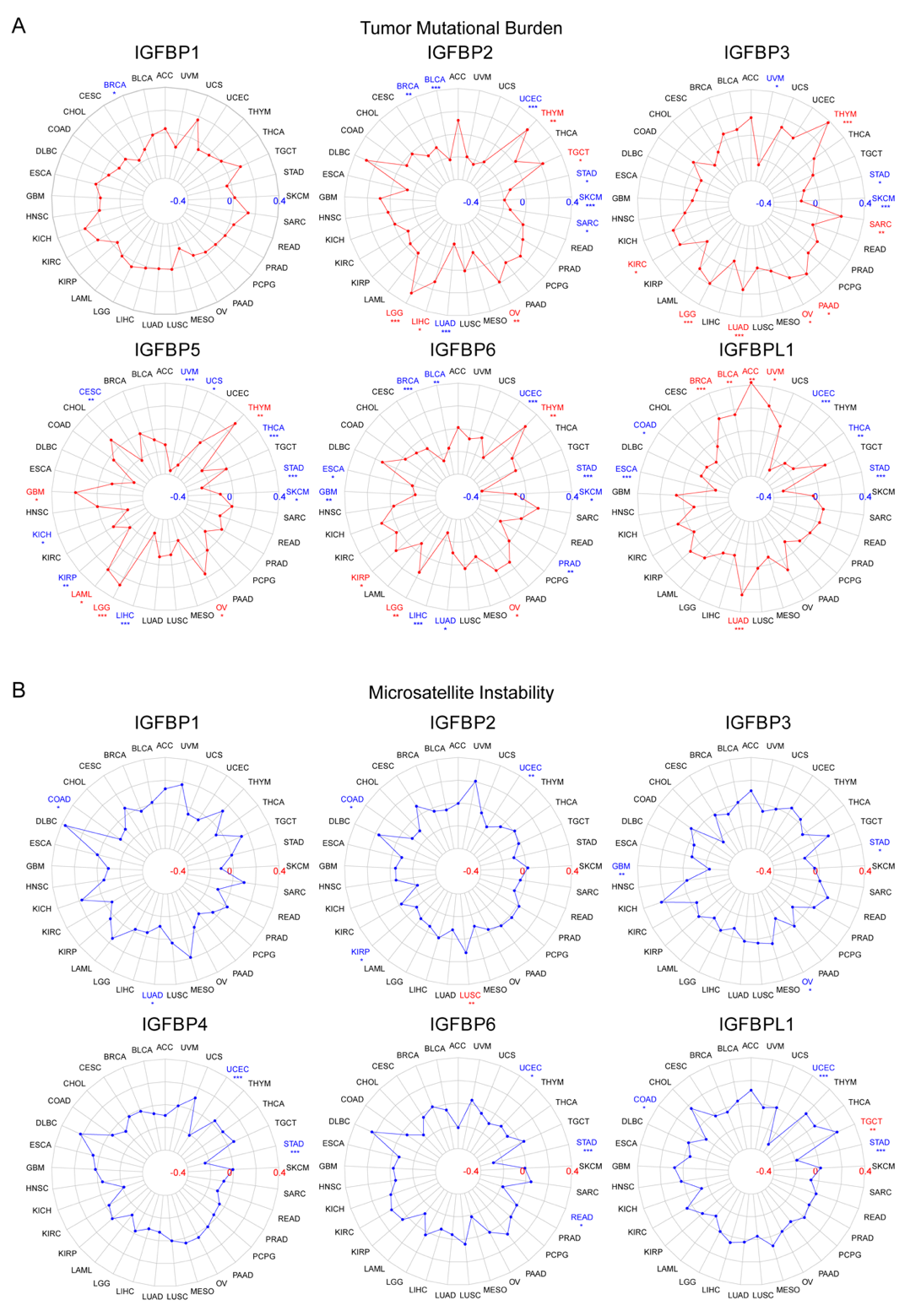


Figure S3. The correlation between IGFBPs expression and (A) TMB and (B) MSI in pan-cancer.


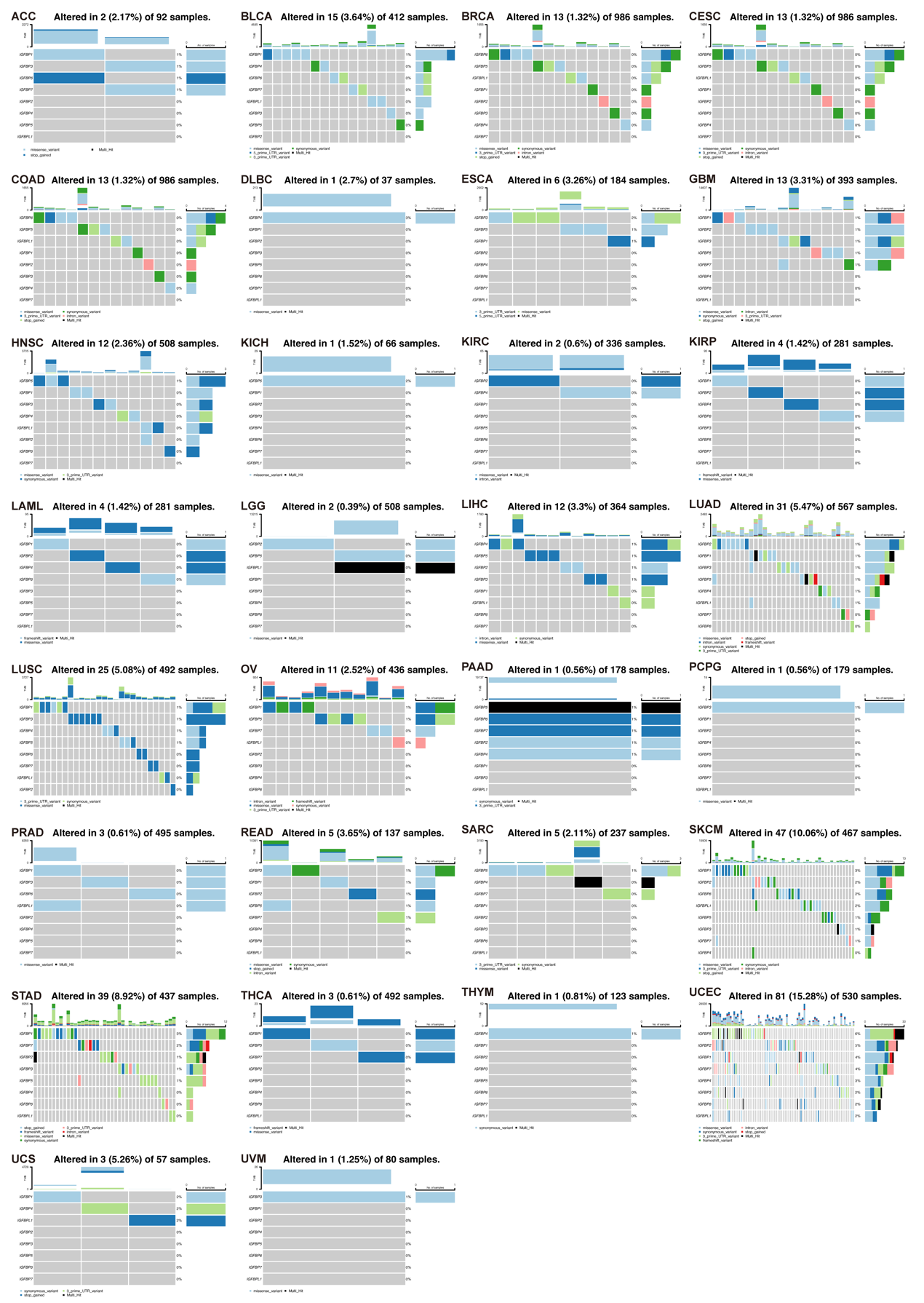


Figure S4. The mutation details of IGFBPs in pan-cancer.
